# Supplementary material for: Prognostic Factors Associated with Breast Cancer-Specific Survival from 1995 to 2022: A Systematic Review and Meta-Analysis of 1,386,663 Cases from 30 Countries
Source: Diseases. 2024 May 23;12(6):111. doi: 10.3390/diseases12060111 (PMC11203054; doi:10.3390/diseases12060111)

Description of Supplementary File S2

This file contain visualiza1ons of Funnel plots generated for each factor to illustrate the distribu1on of publica1on bias, corresponding to the Egger's test results, which indicated that publica1on bias is present in age 35 to 60, stage 3, undifferen1ated cancer cell, tumour size, overweight/obese, chemotherapy, and radiotherapy.

Funnel plot (Age below 35)

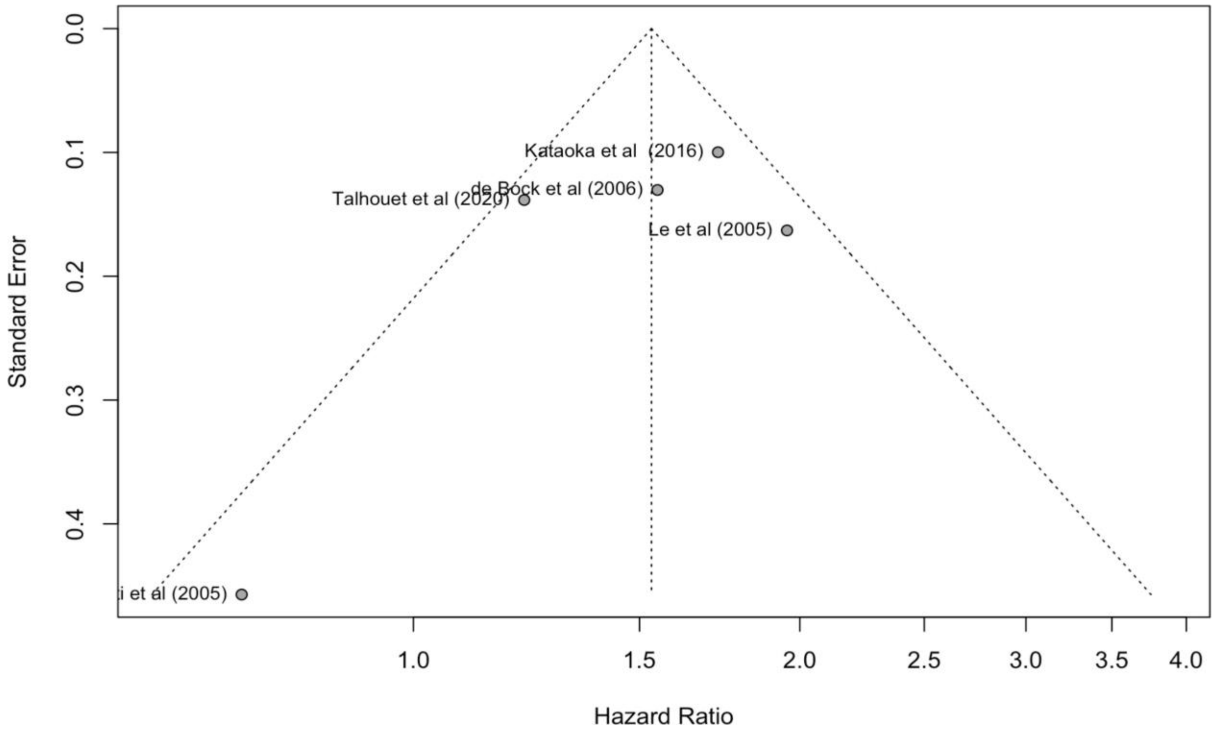

Funnel plot (Age 35 to 60)

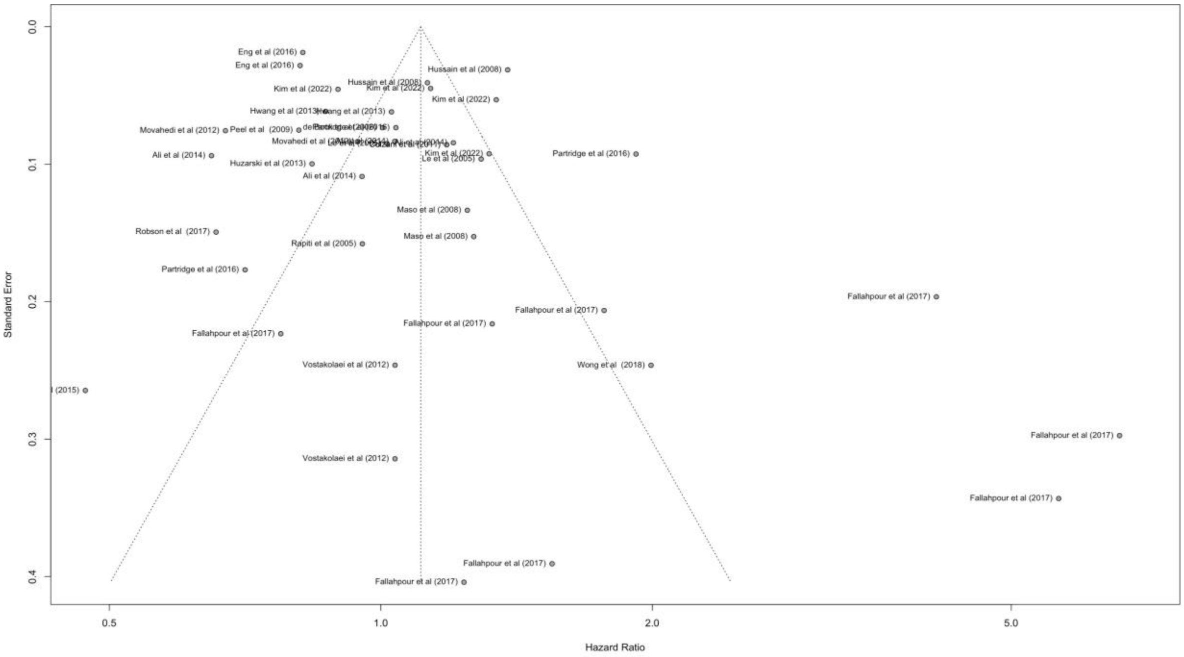

Funnel plot (Above 60)

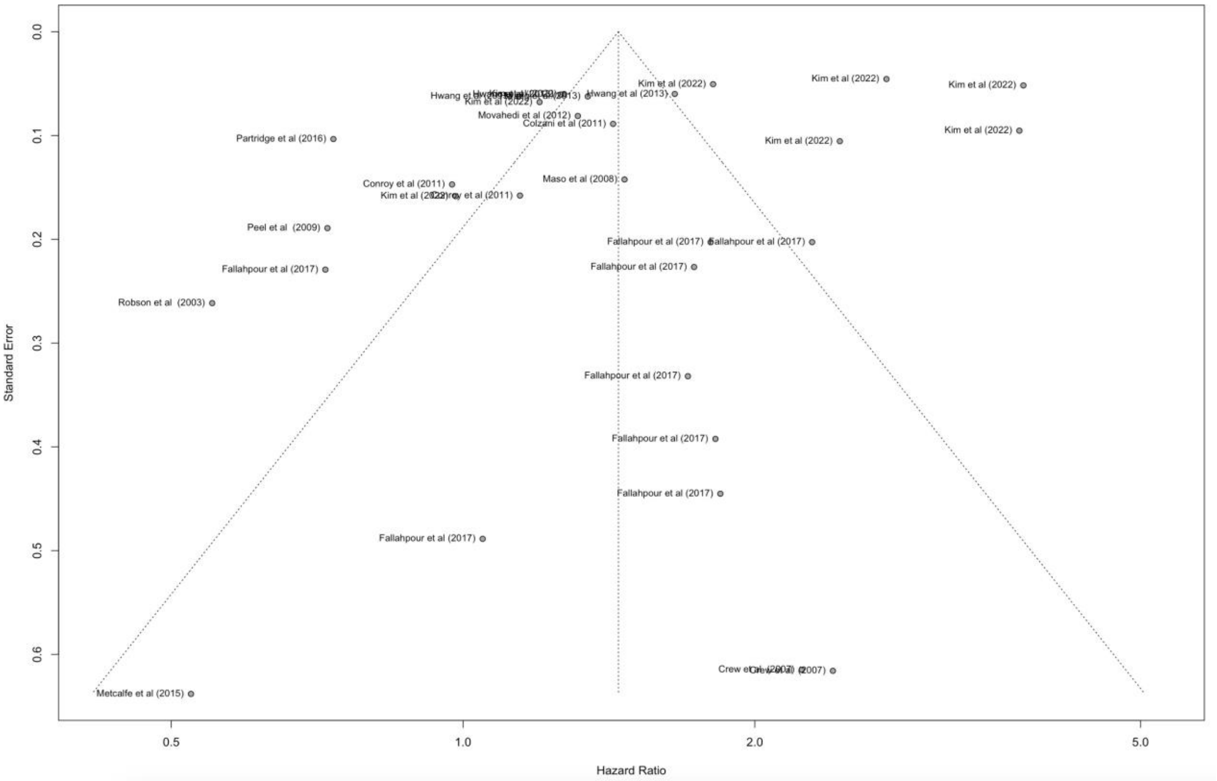

Funnel plot (Educa(on - Secondary)

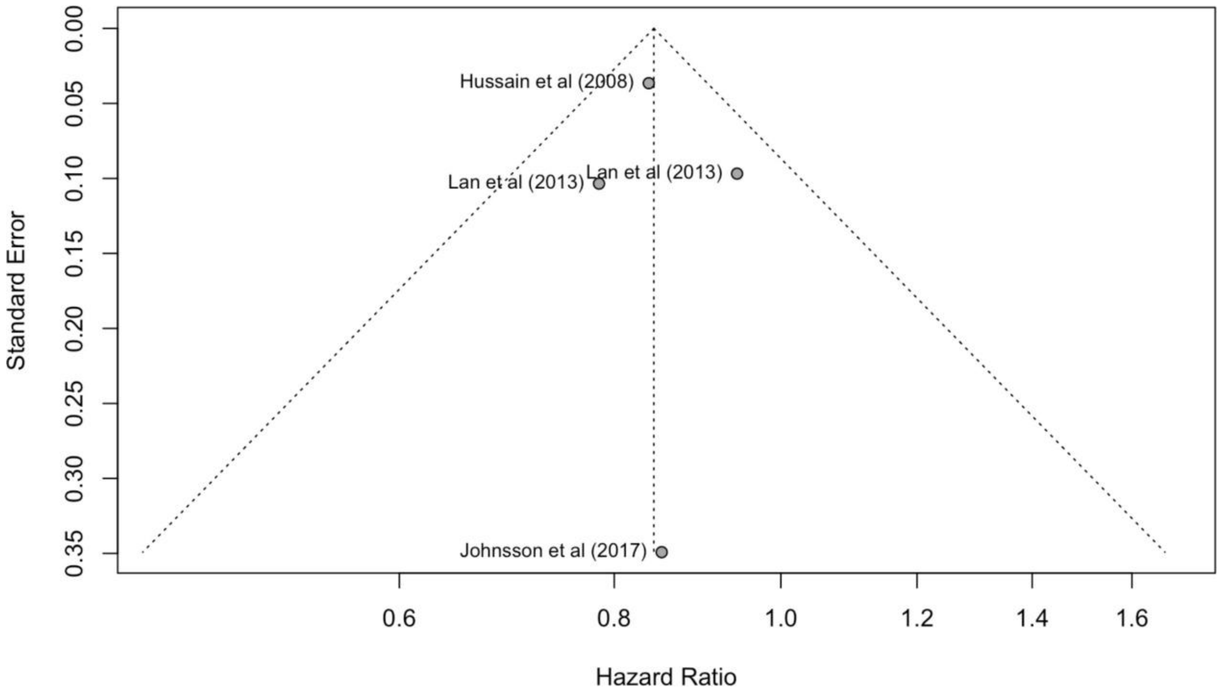

Funnel plot (Educa(on - Higher)

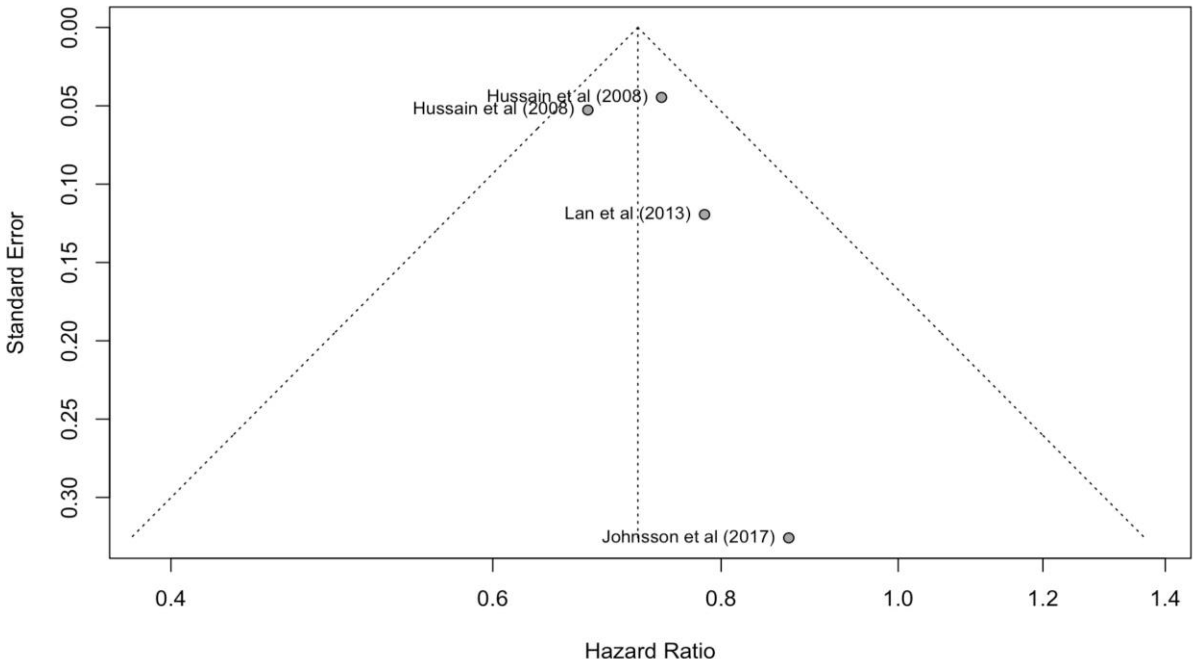

Funnel plot (Race - Black)

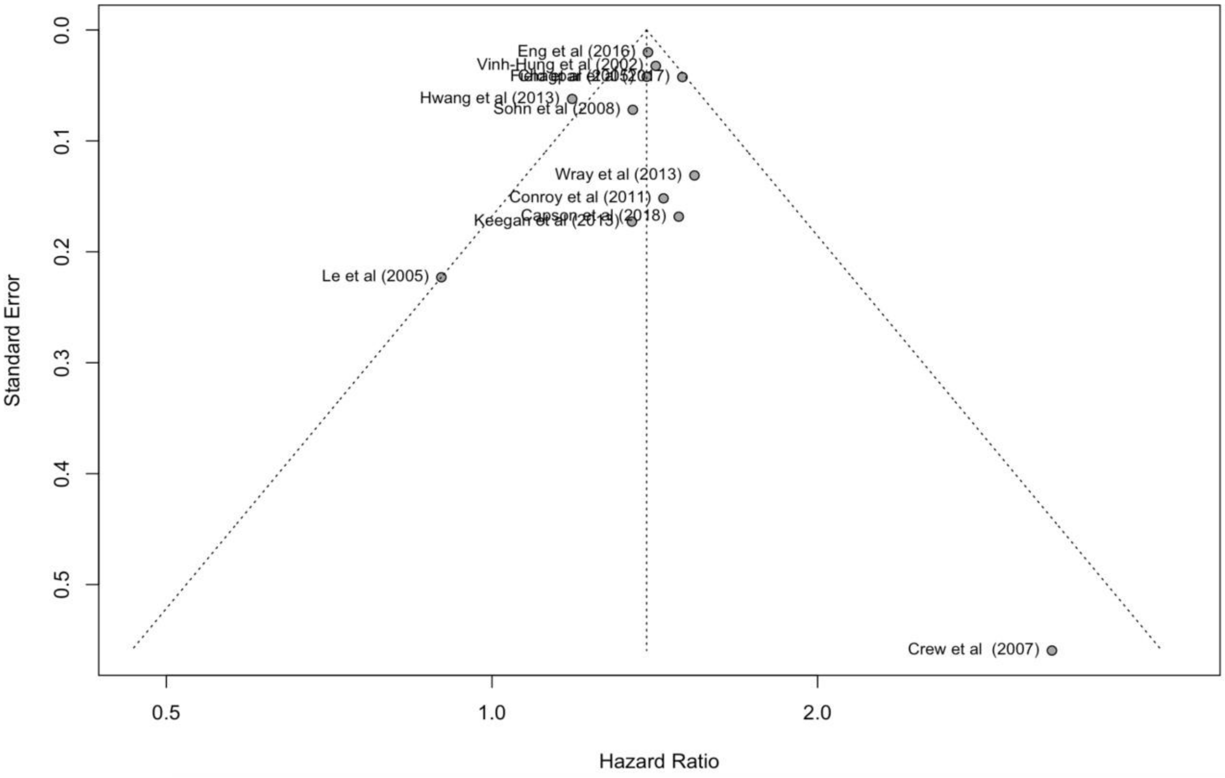

Funnel plot (Race - Asian)

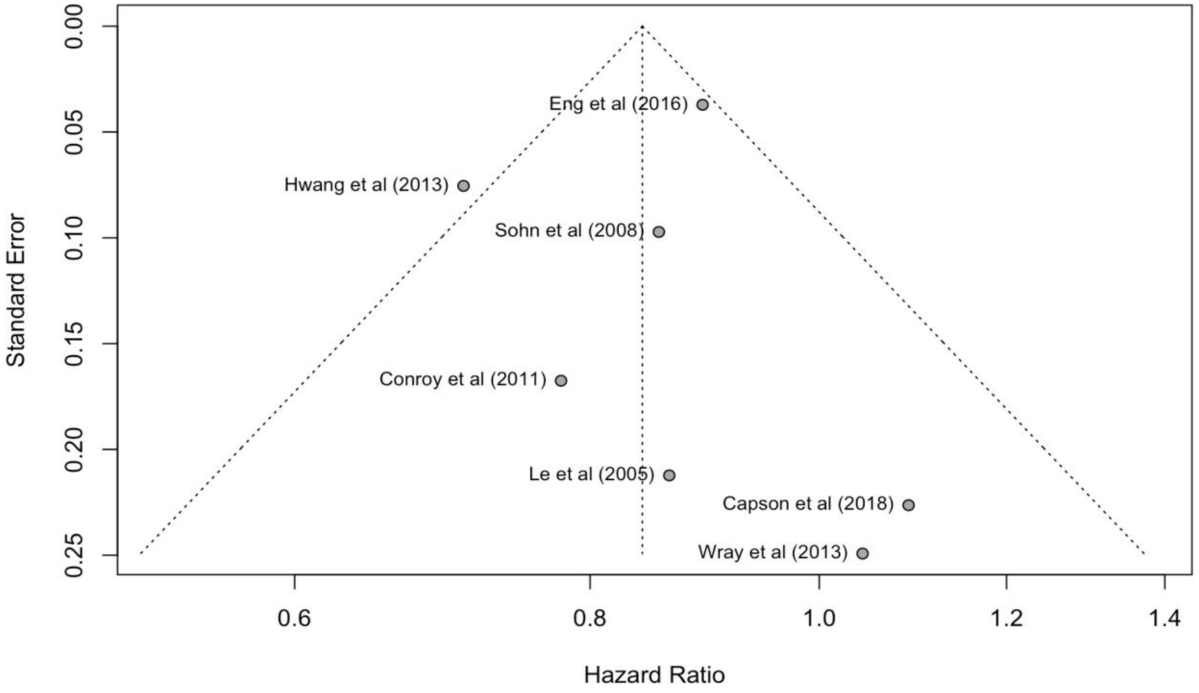

Funnel plot (Race - Hispanic)

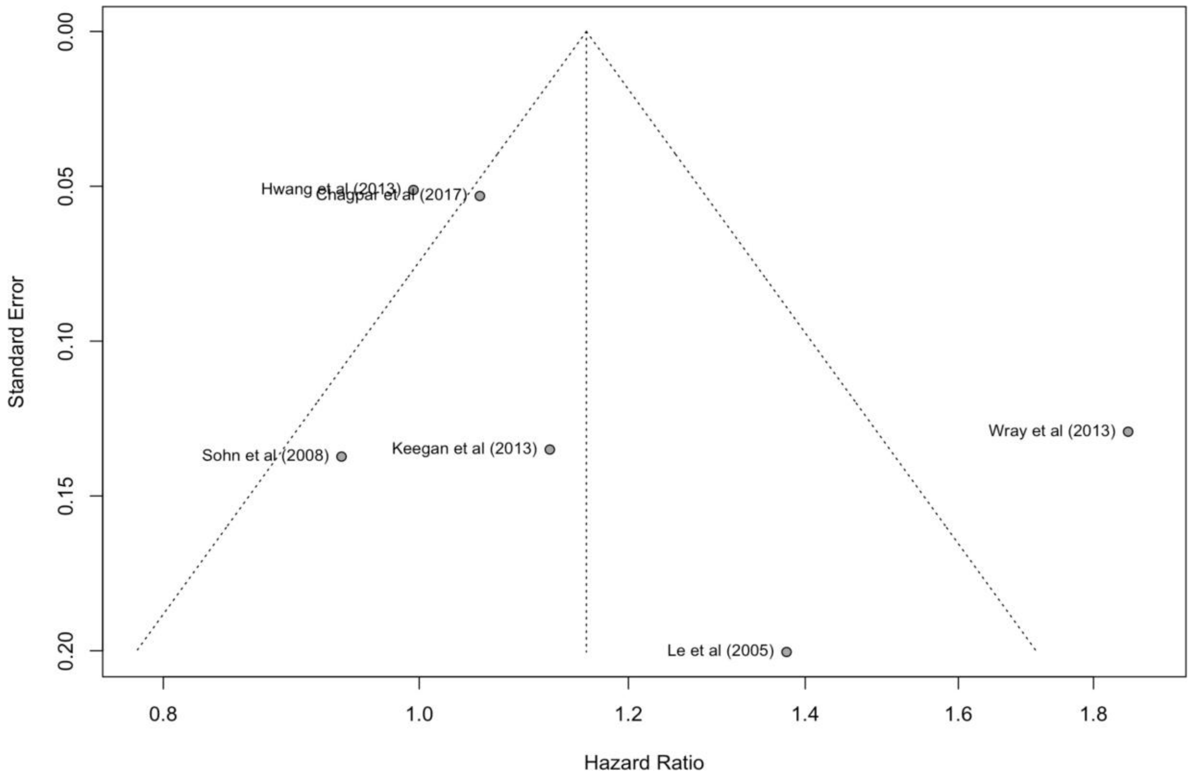

Funnel plot (Grade 2)

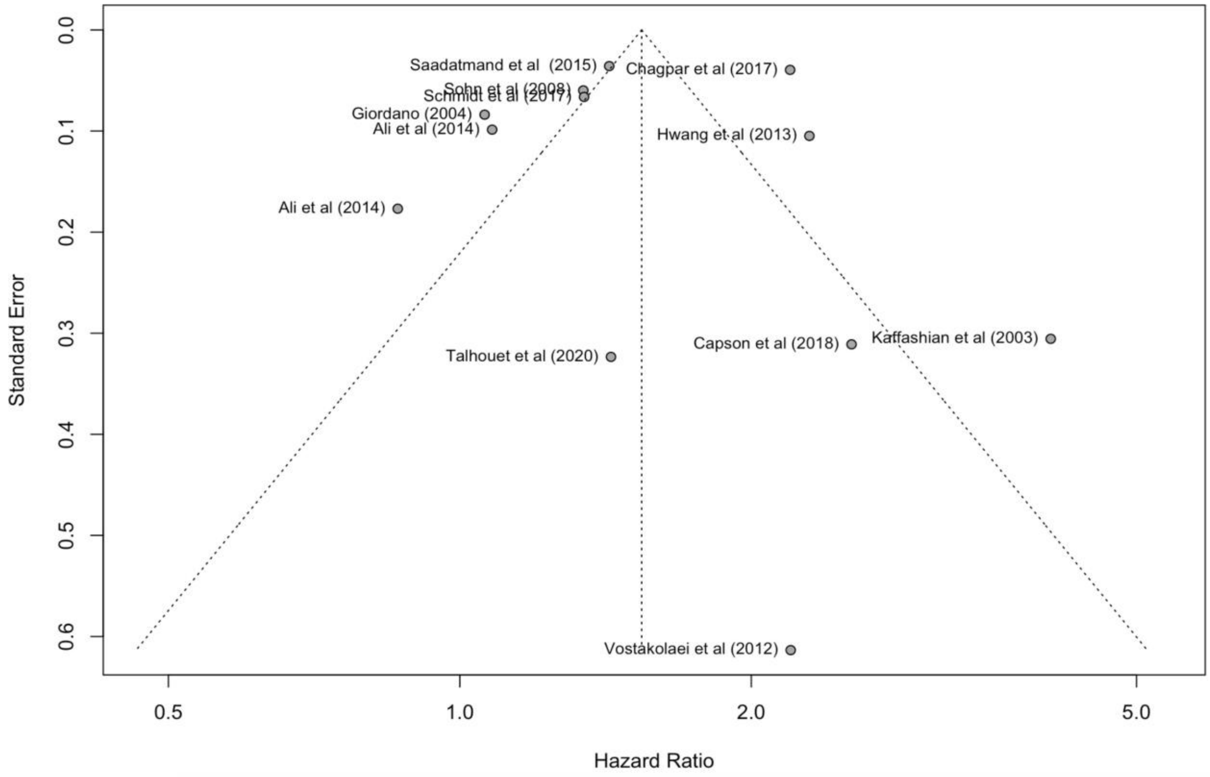

Funnel plot (Grade 3)

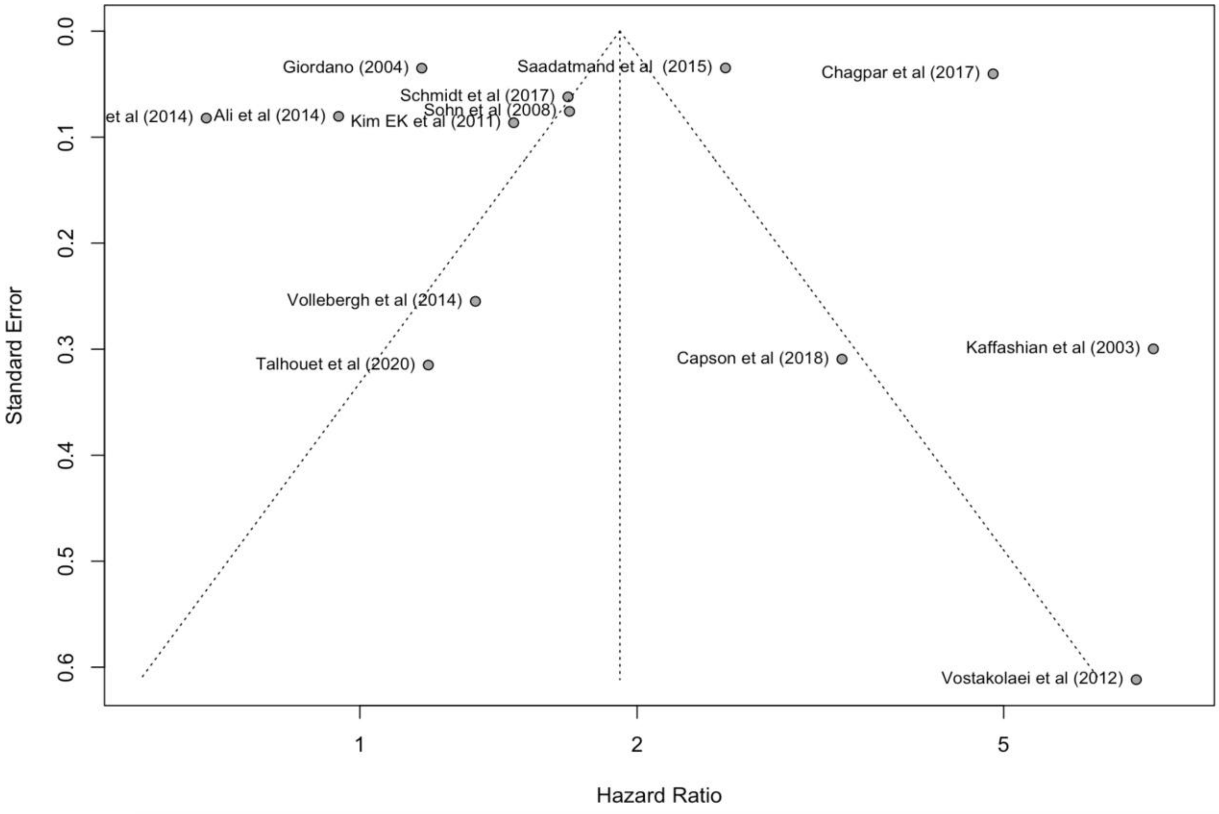

Funnel plot (Stage 2)

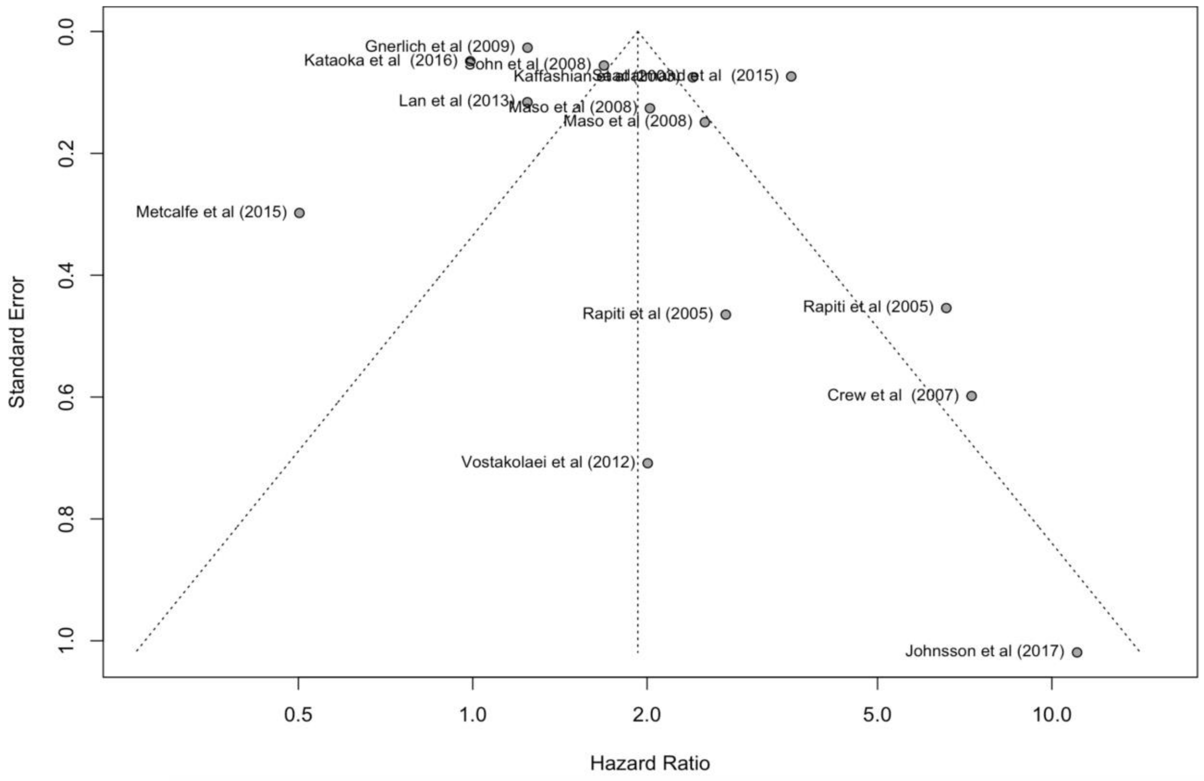

Funnel plot (Stage 3)

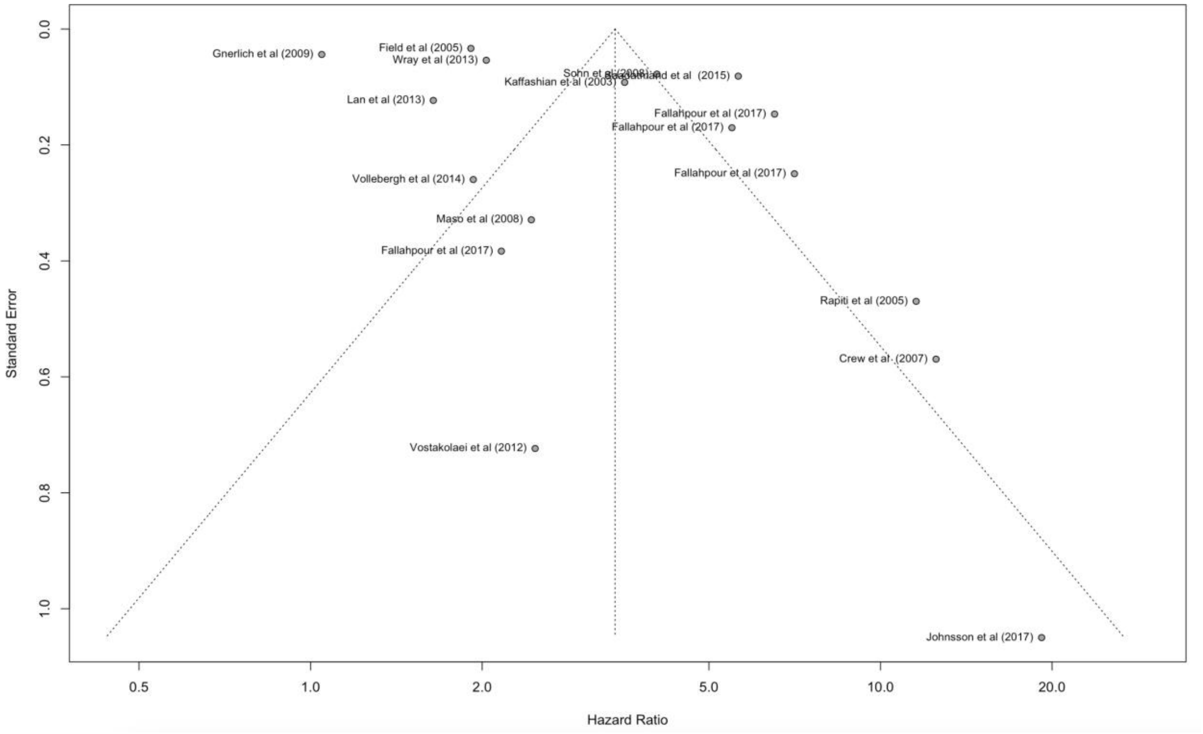

Funnel plot (Stage 4)

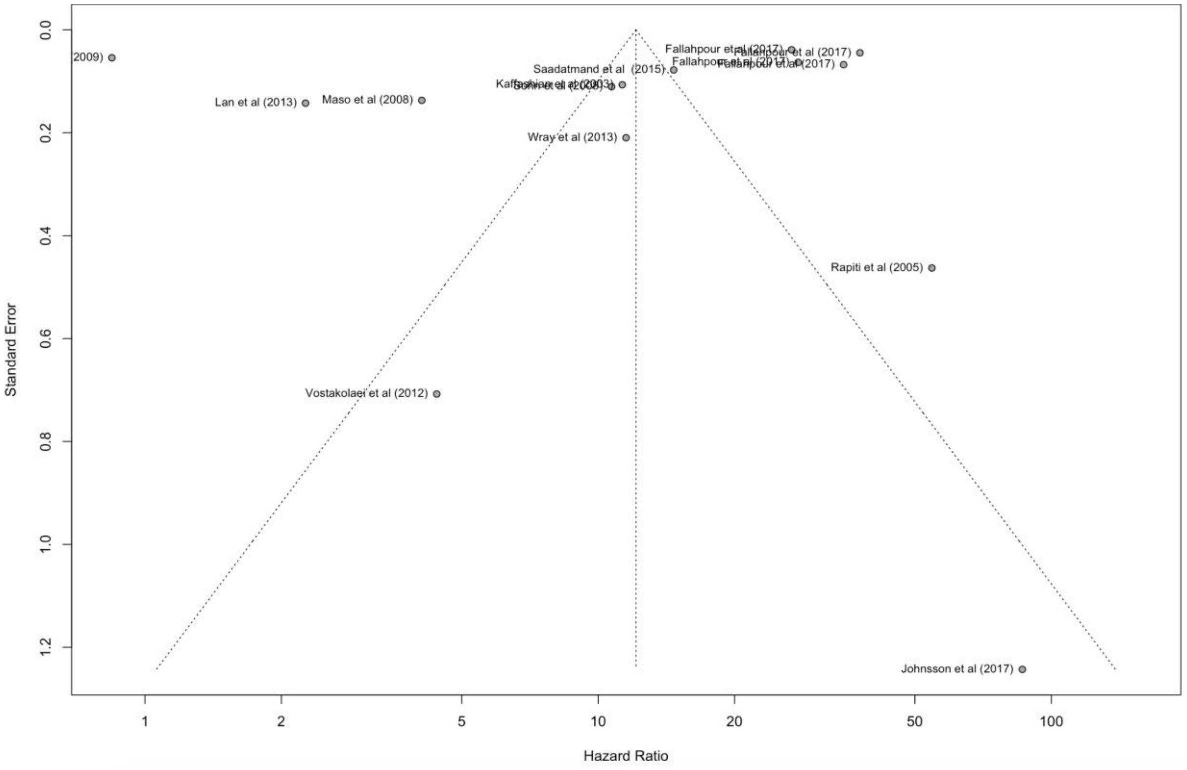

Funnel plot (Differen(a(on - Moderate)

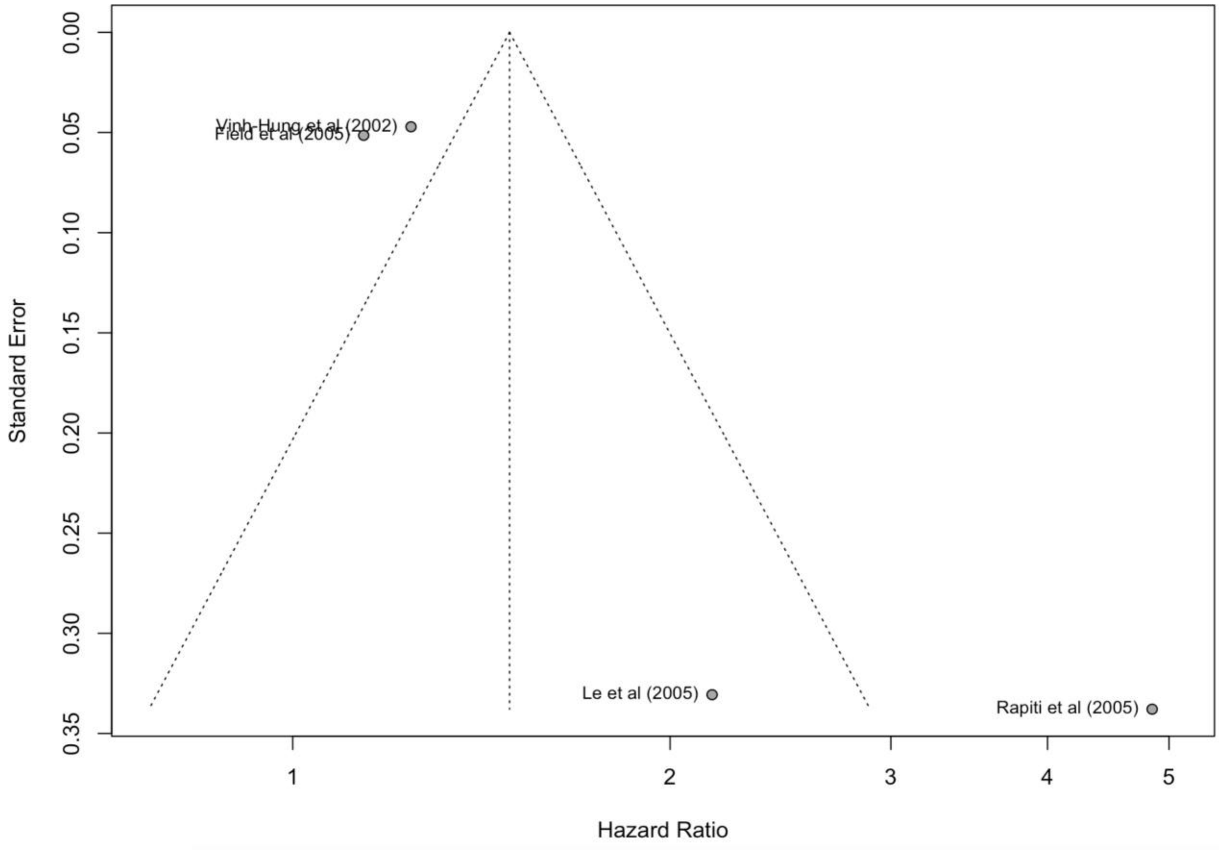

Funnel plot (Differen(a(on - Poor)

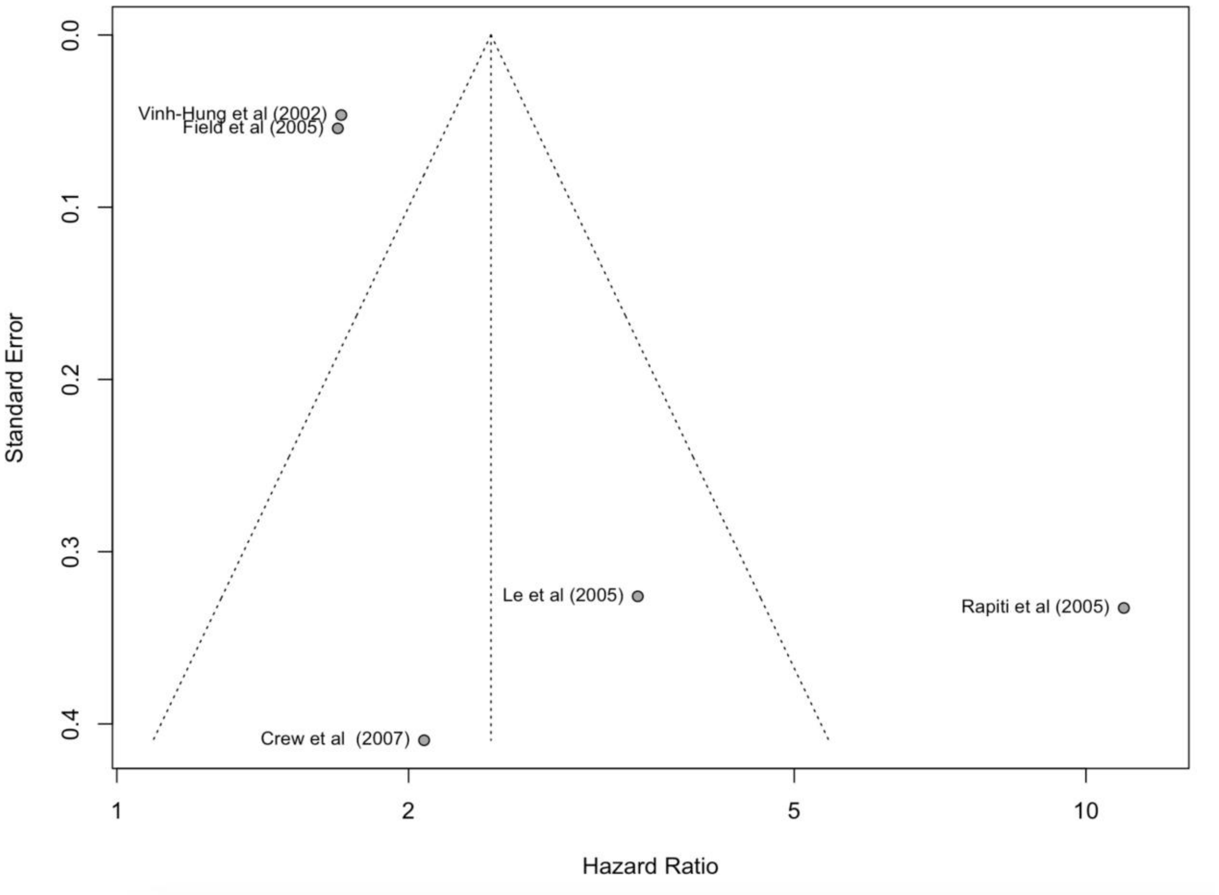

Funnel plot (Differen(a(on - Undifferen(ated)

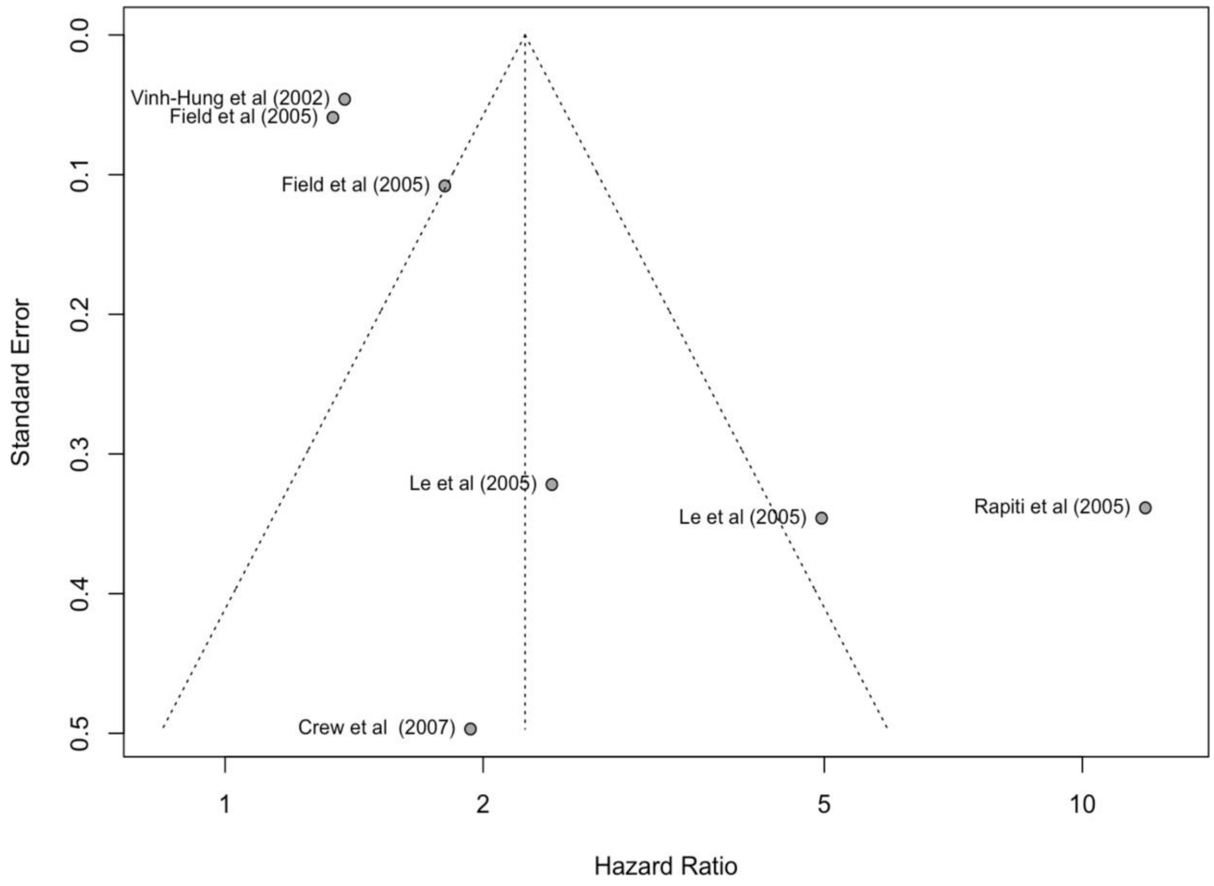

Funnel plot (Nodes - Posi(ve)

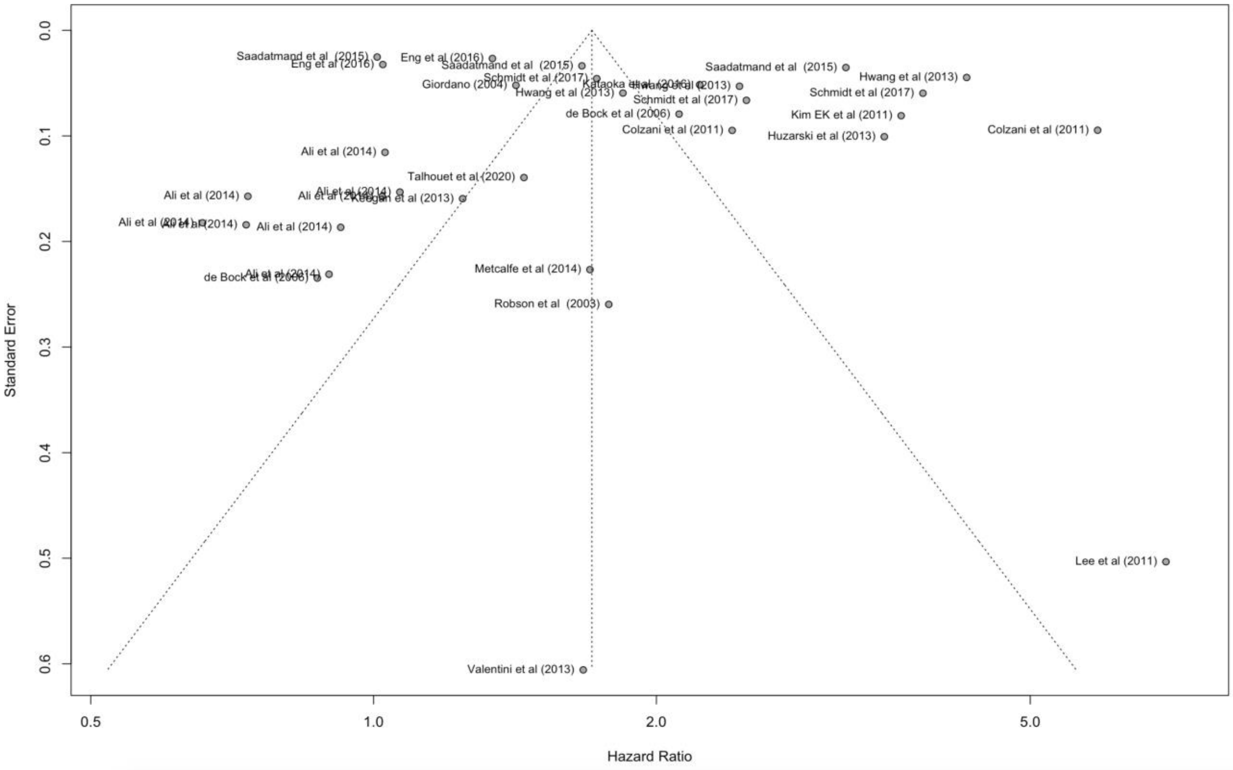

Funnel plot (Surgery - Yes)

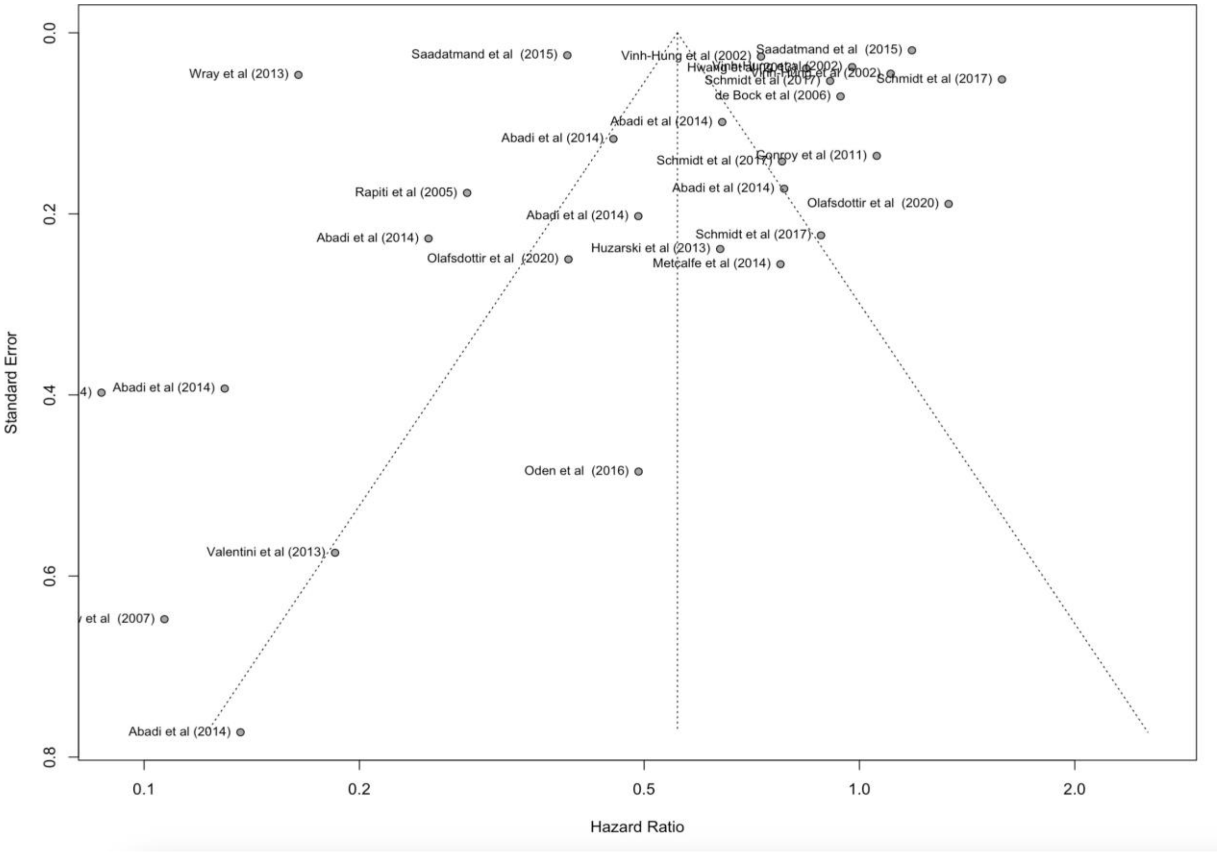

Funnel plot (Tumour size ≥ 2 cm)

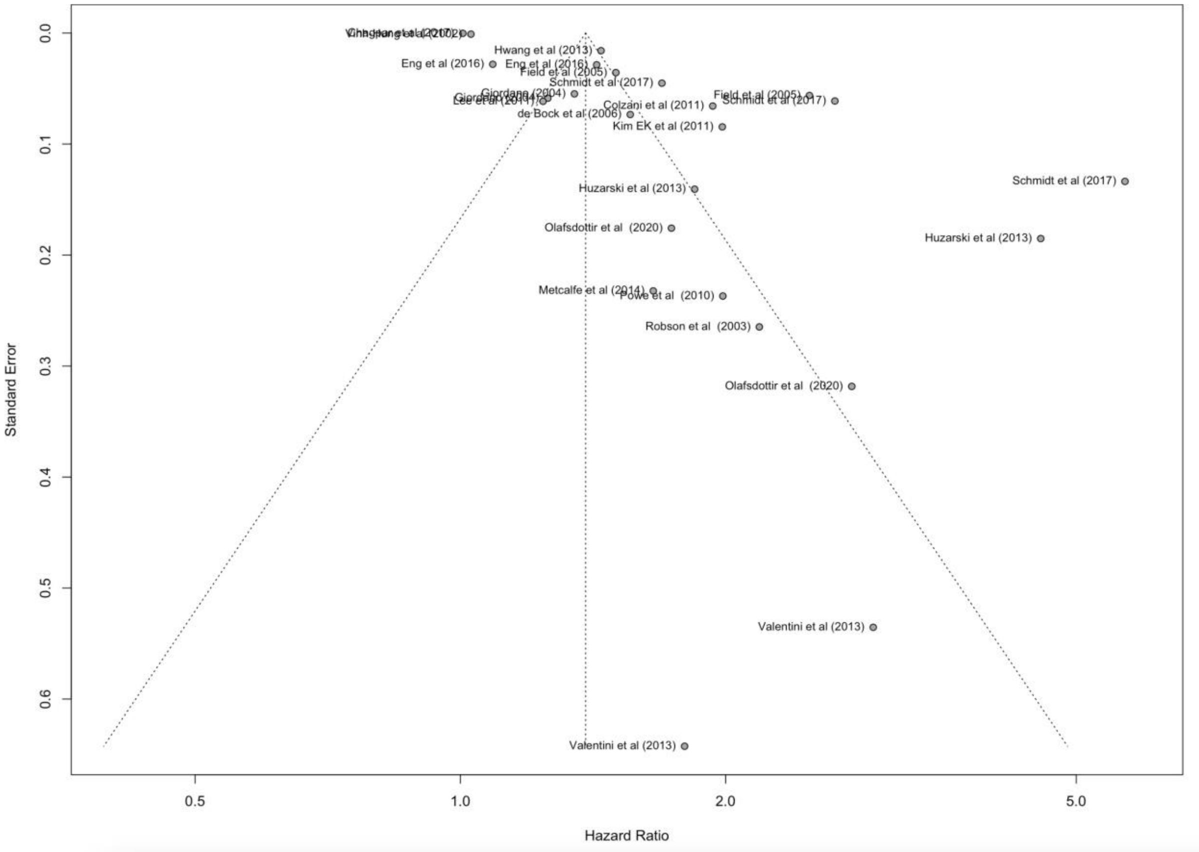

Funnel plot (Hormone Therapy - Yes)

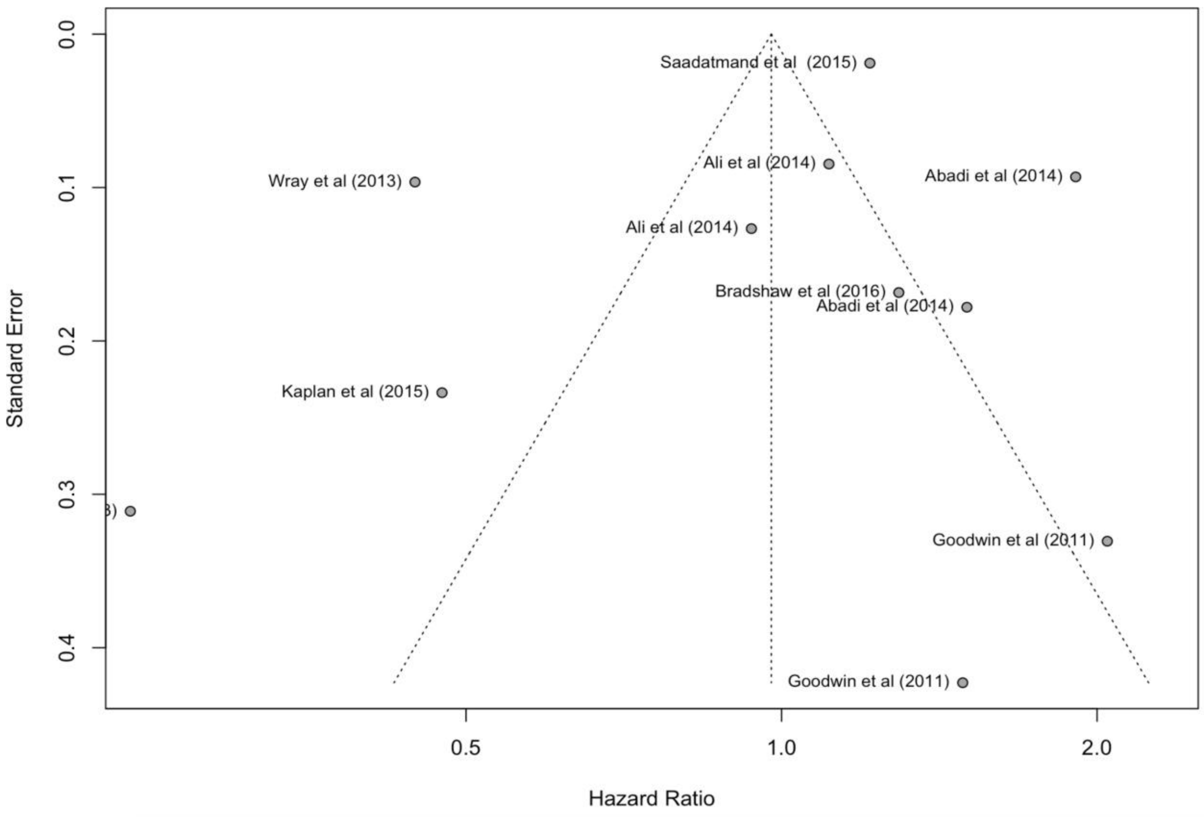

Funnel plot (Chemotherapy - Yes)

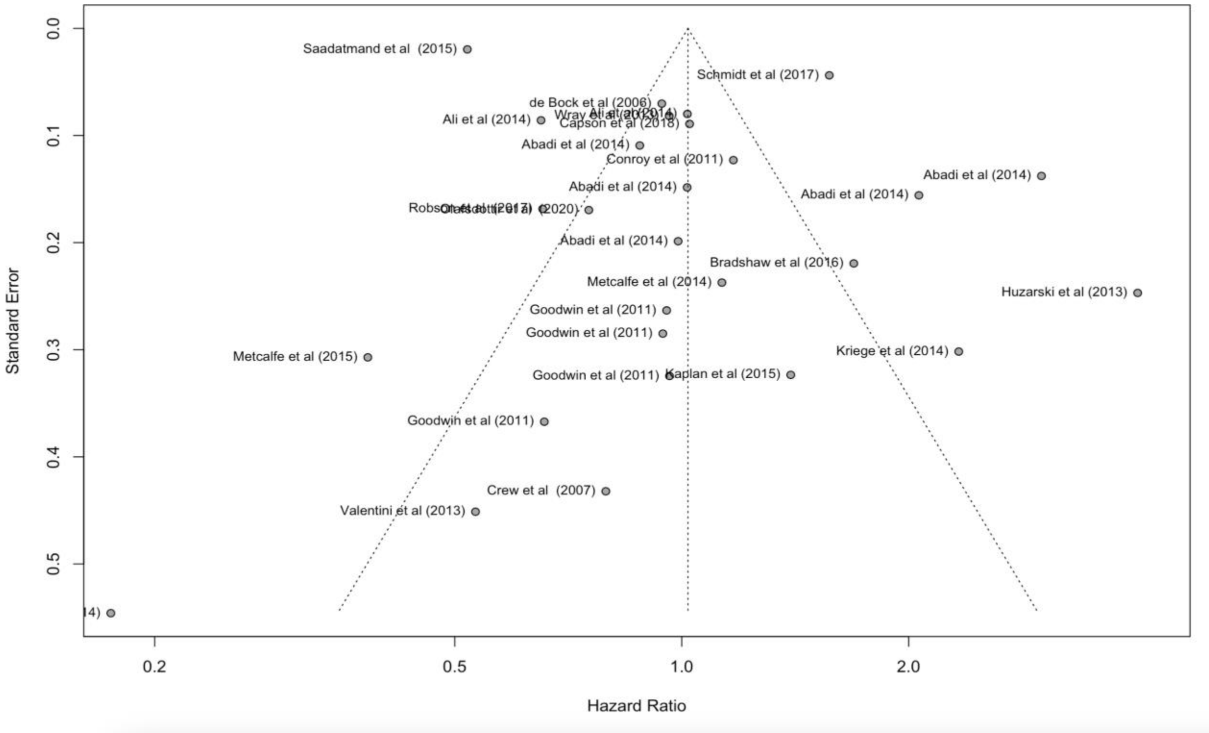

Funnel plot (Radiotherapy - Yes)

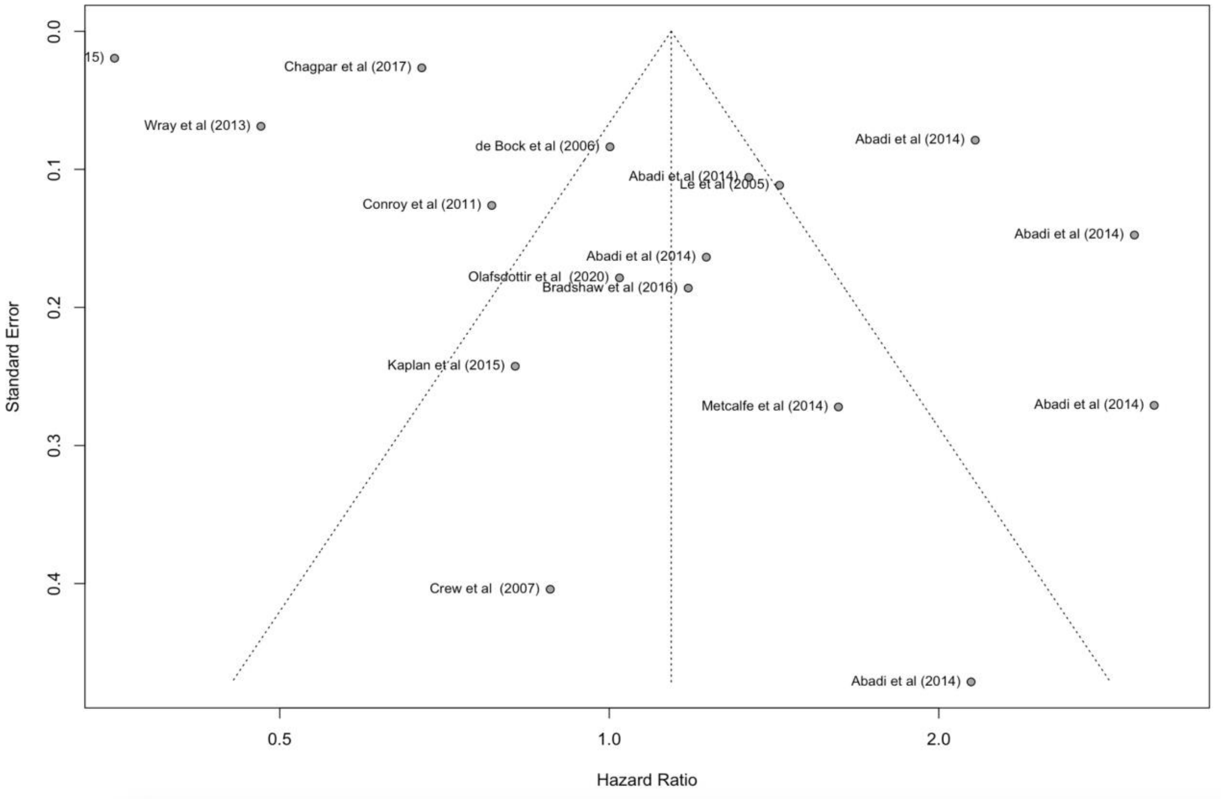

Funnel plot (Tamoxifen)

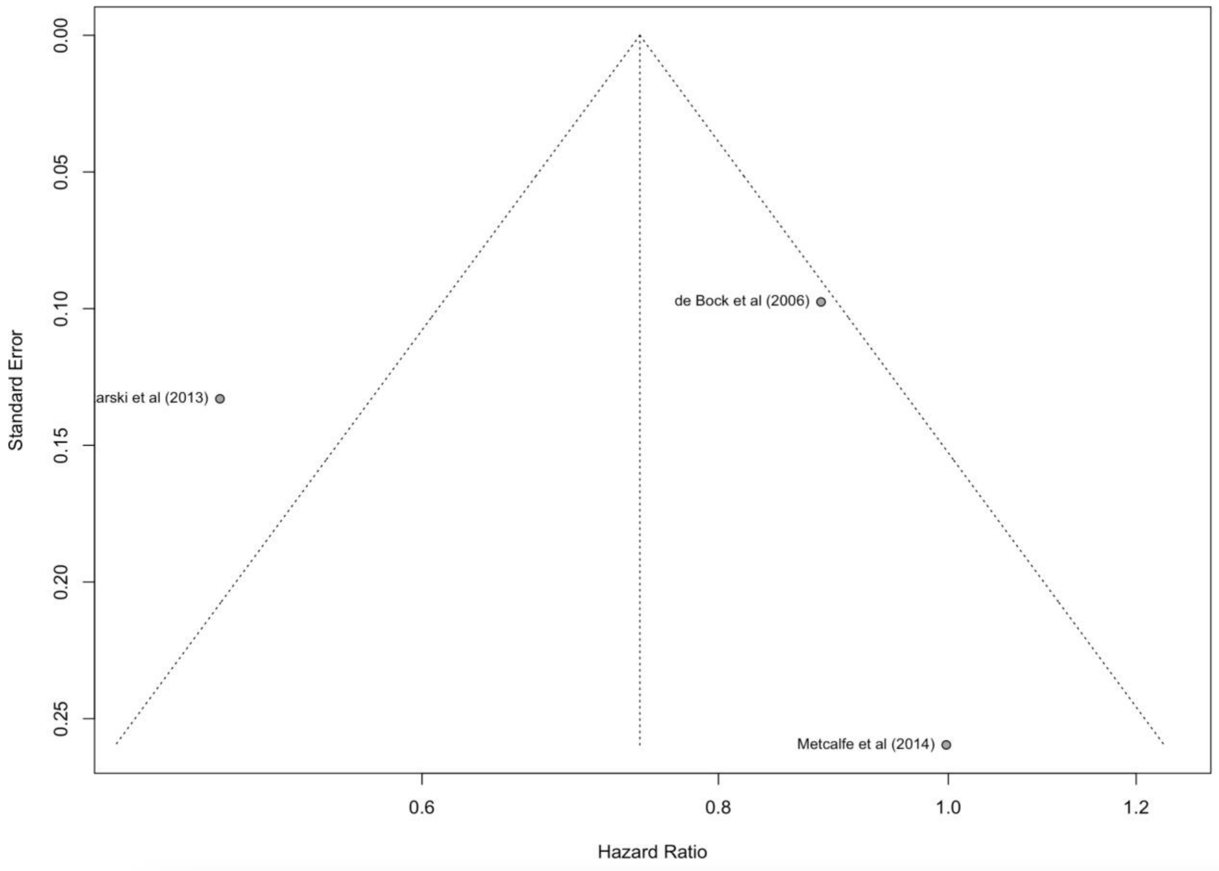

Funnel plot (Histology - lobular)

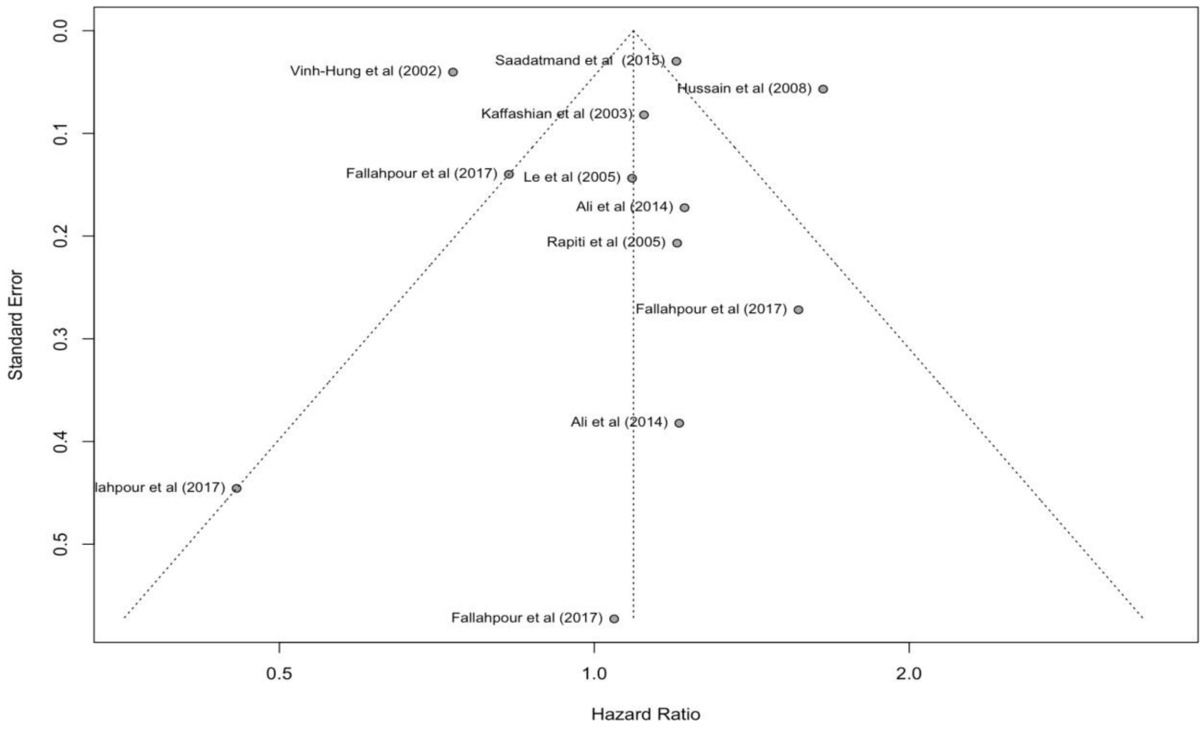

Funnel plot (Histology - Medullary)

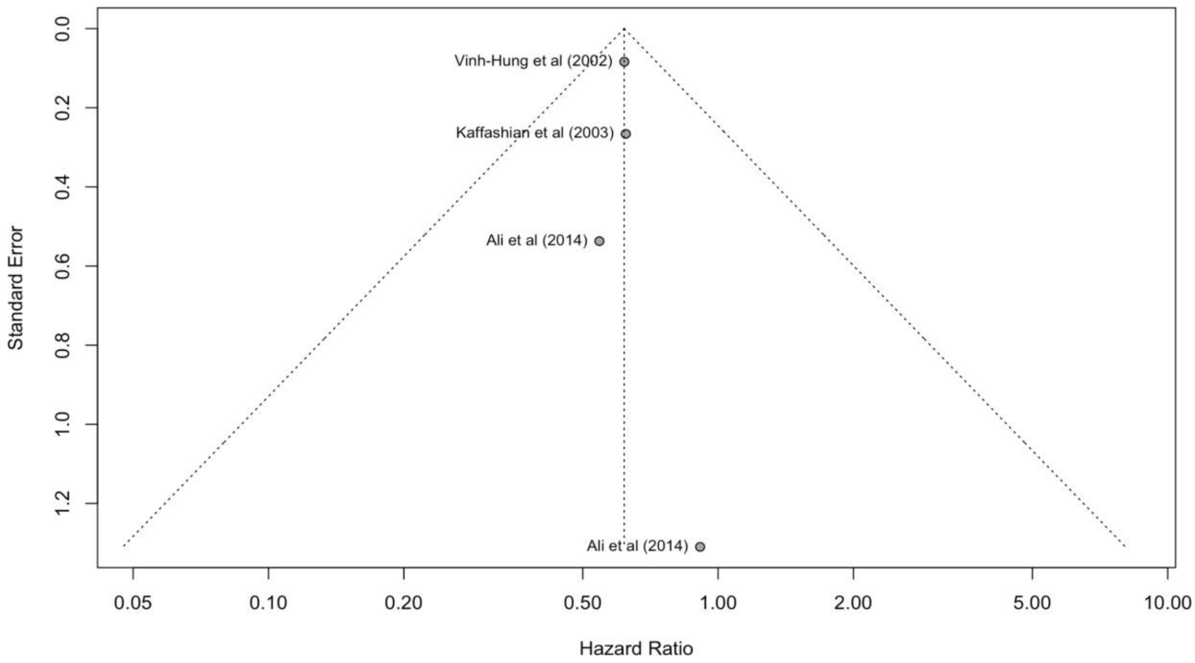

Funnel plot (Histology - Others)

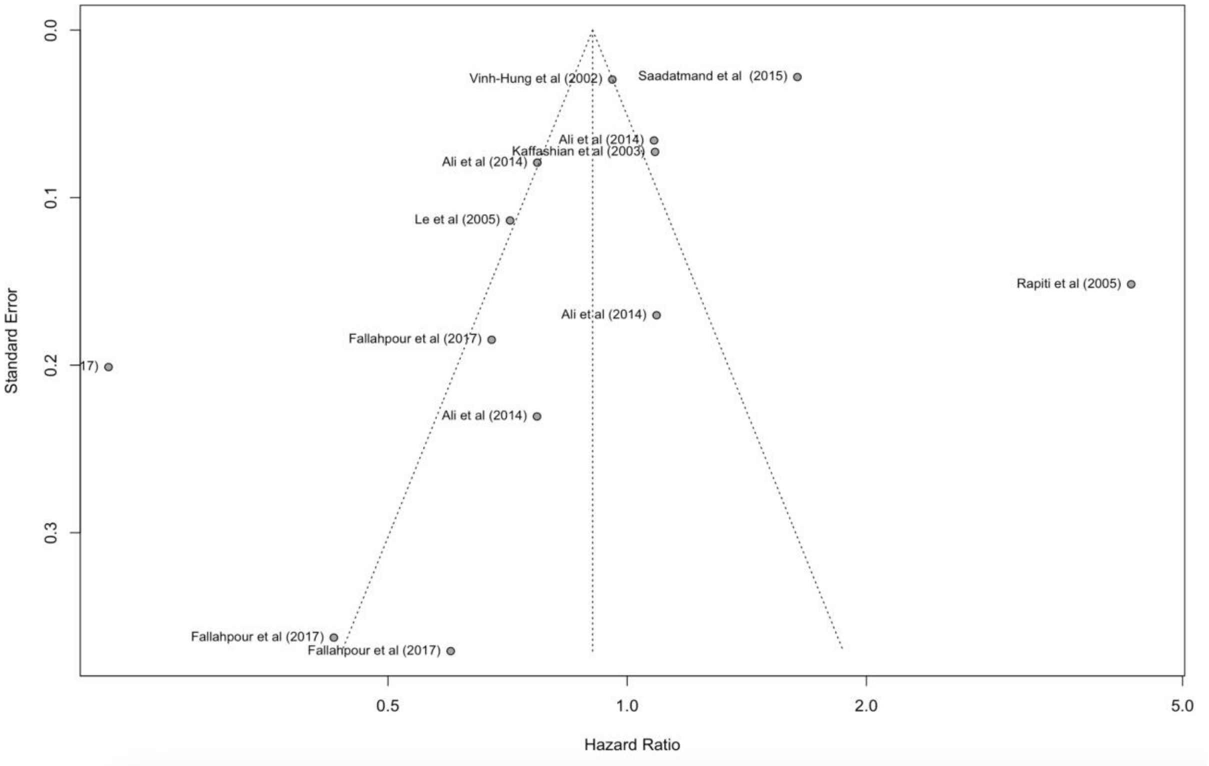

Funnel plot (Progesterone receptor - posi(ve)

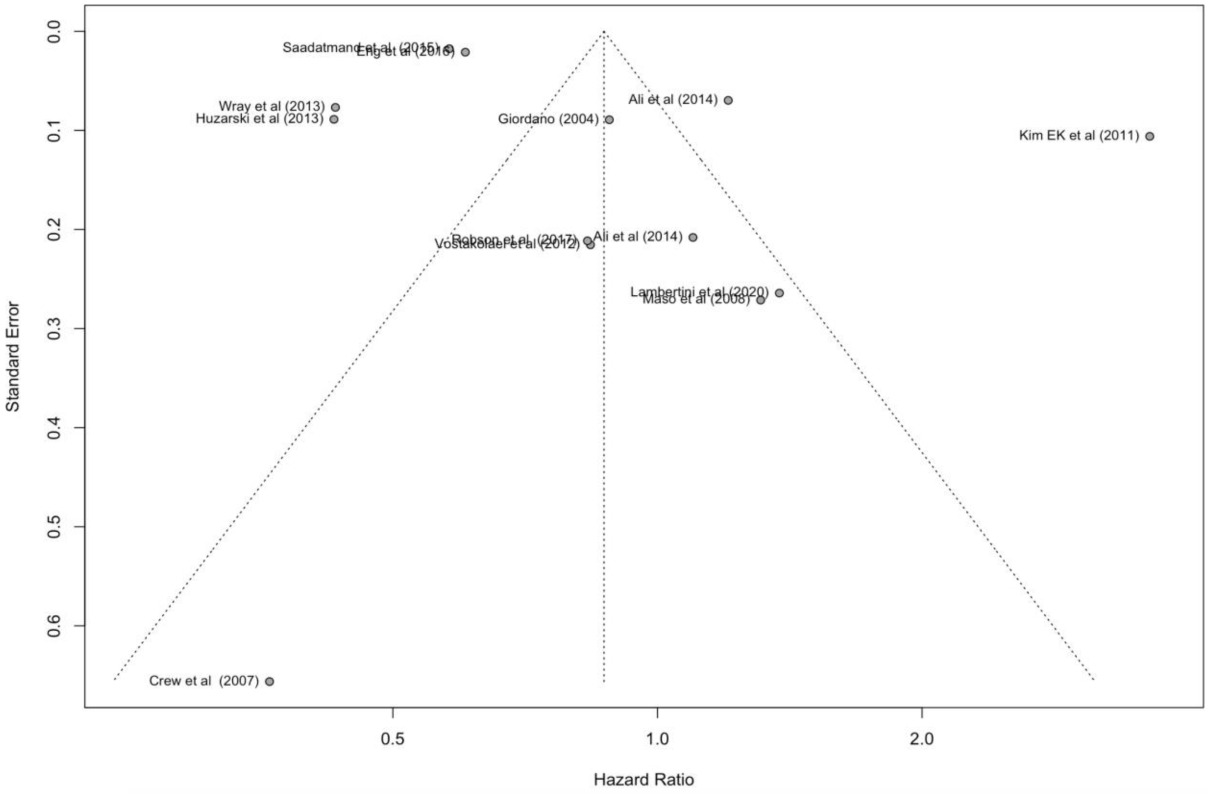

Funnel plot (Progesterone receptor - nega(ve)

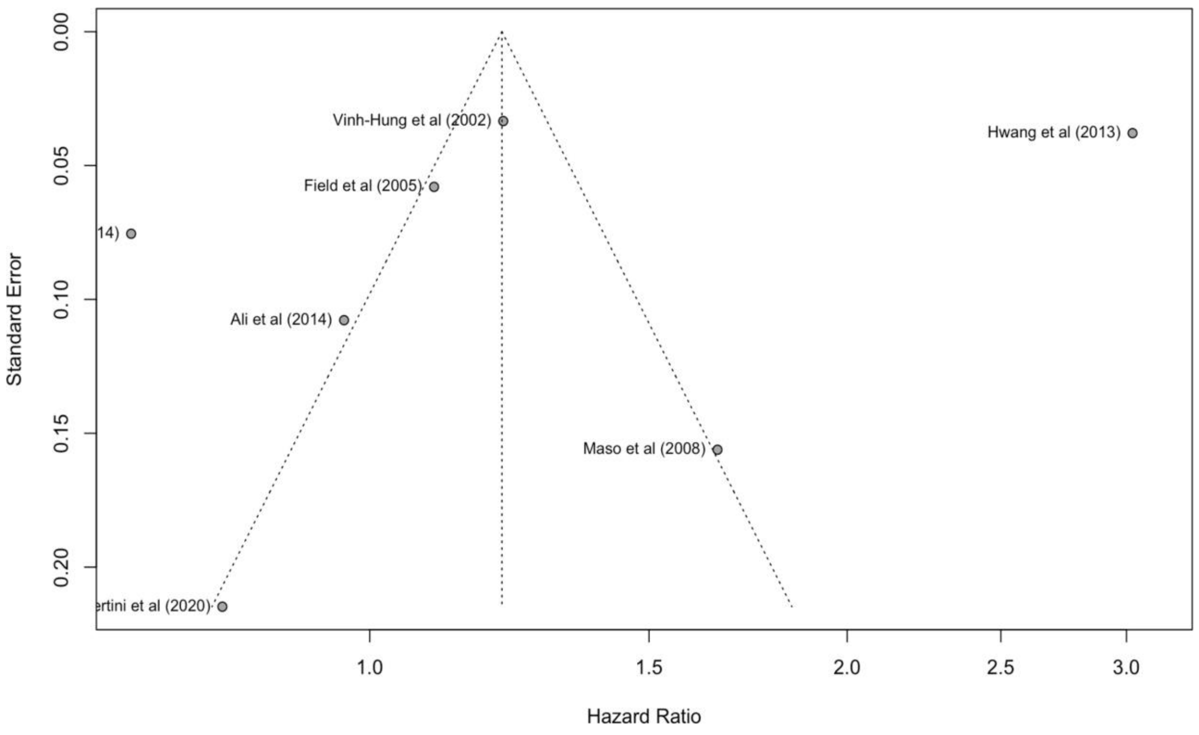

Funnel plot (Estrogen receptor - posi(ve)

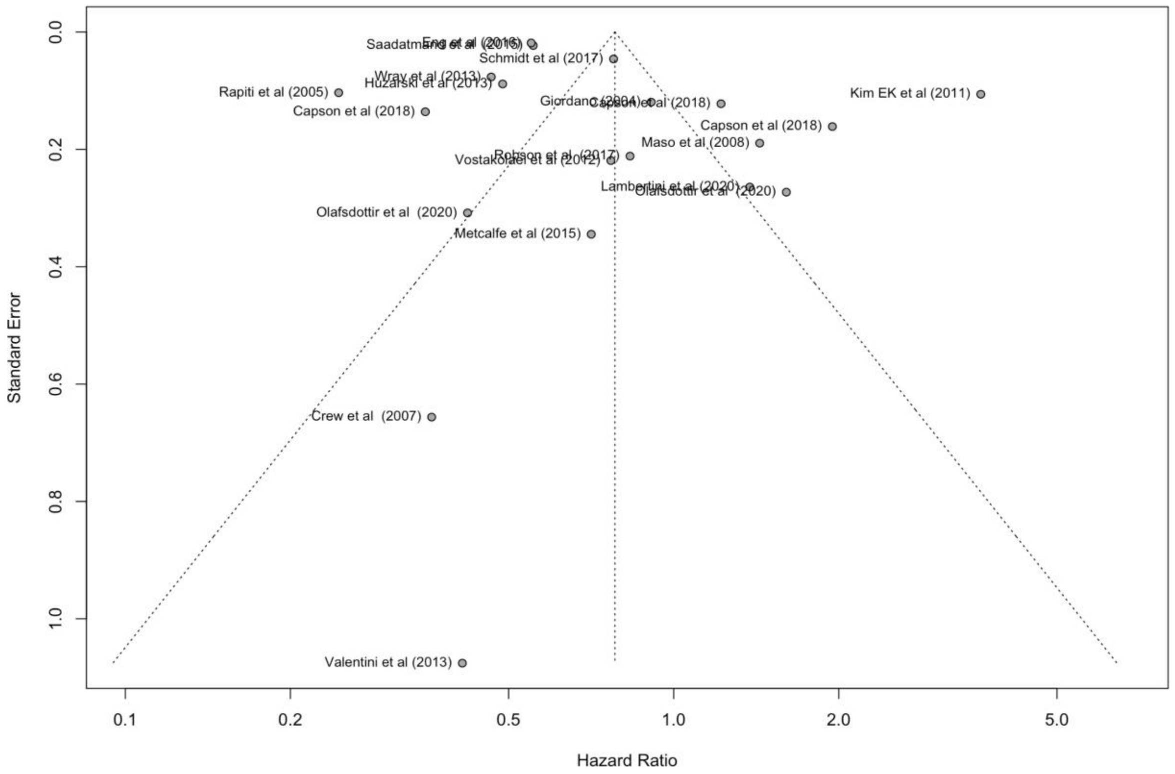

Funnel plot (Estrogen receptor - nega(ve)

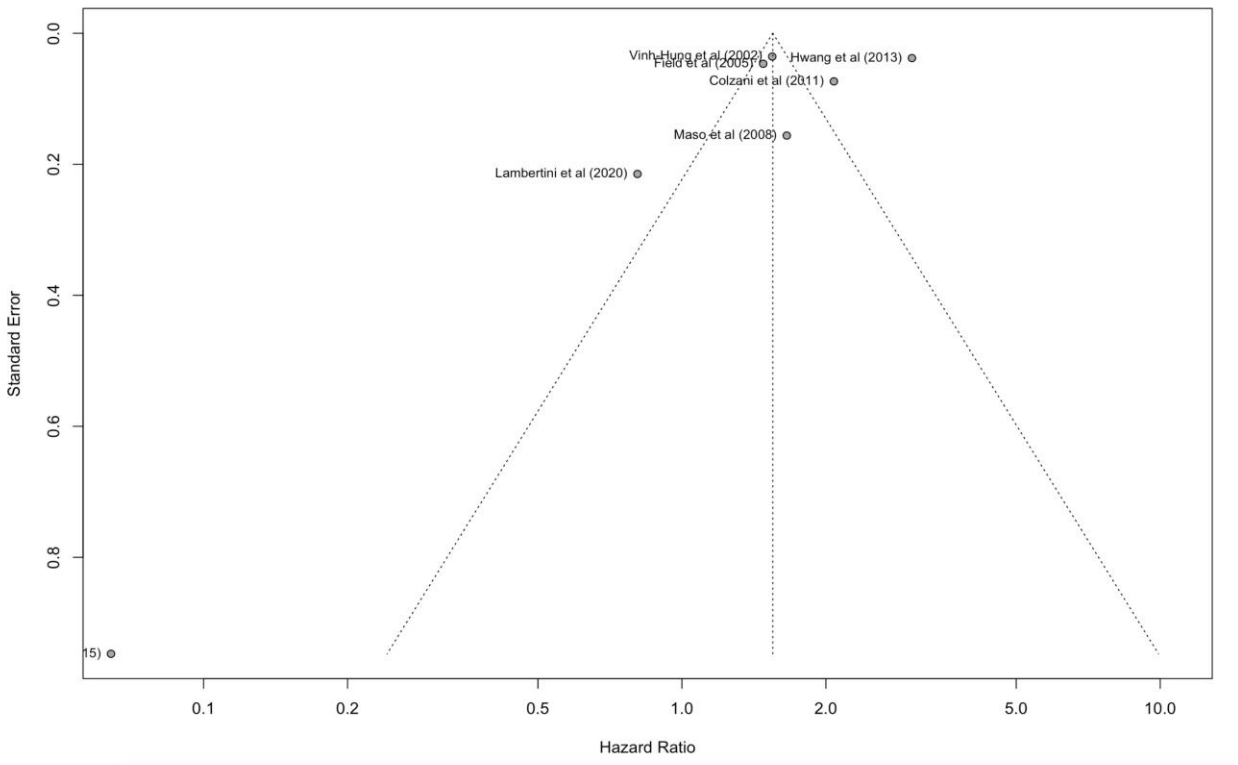

Funnel plot (HER2 receptor - posi(ve)

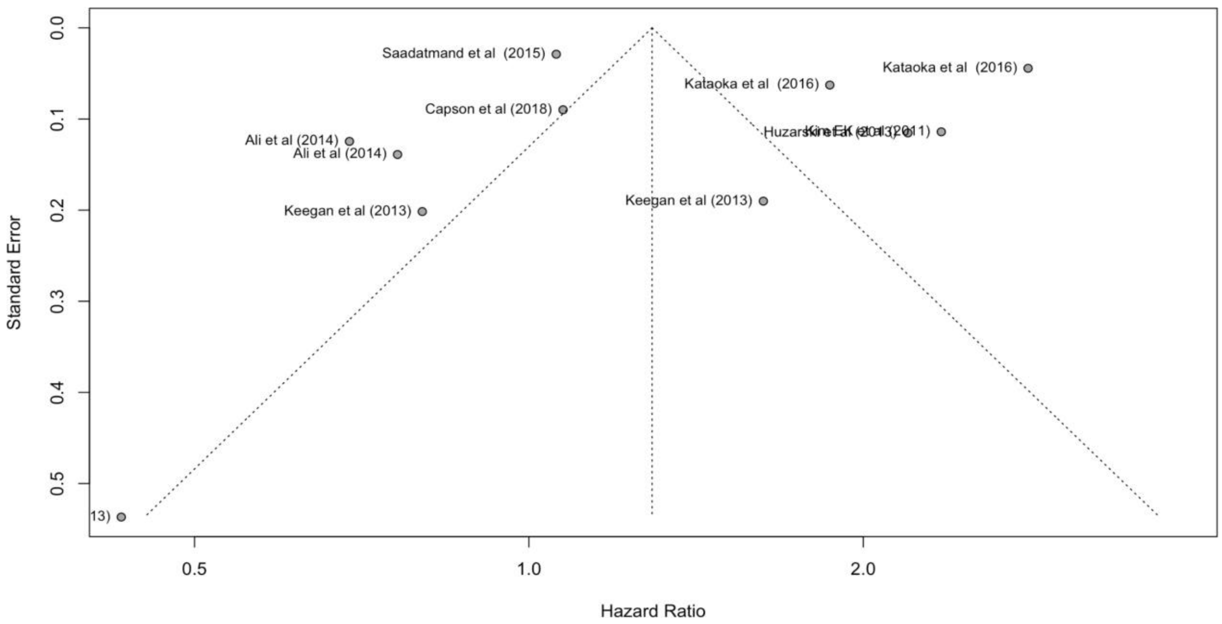

Funnel plot (HER2 receptor - nega(ve)

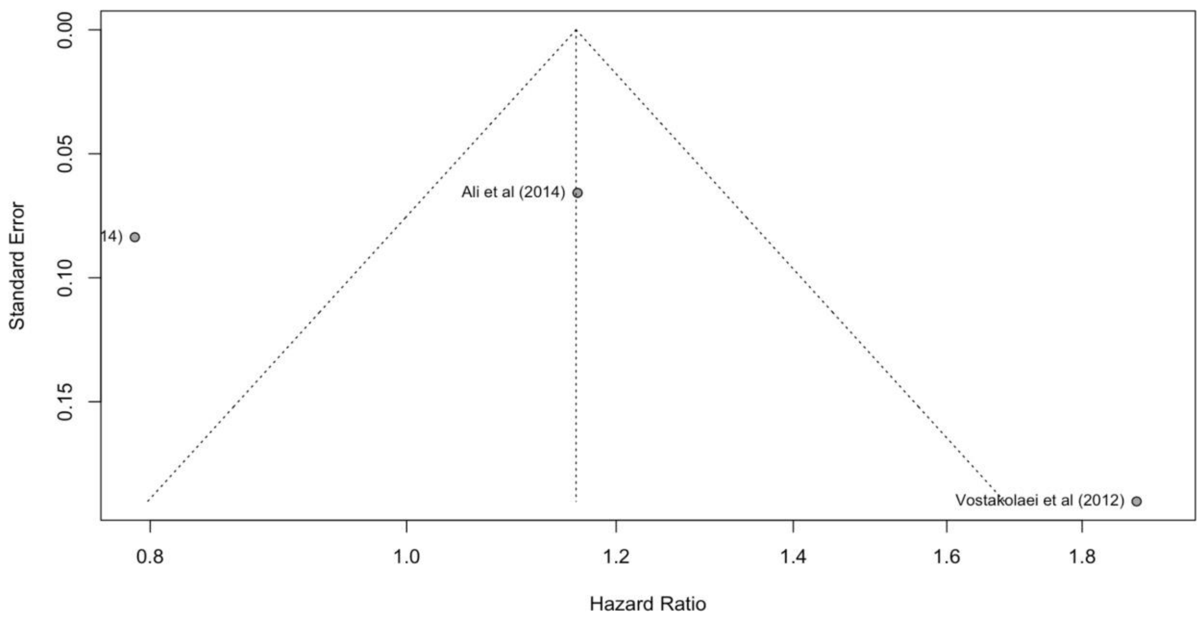

Funnel plot (Physical ac(vity – light to moderate)

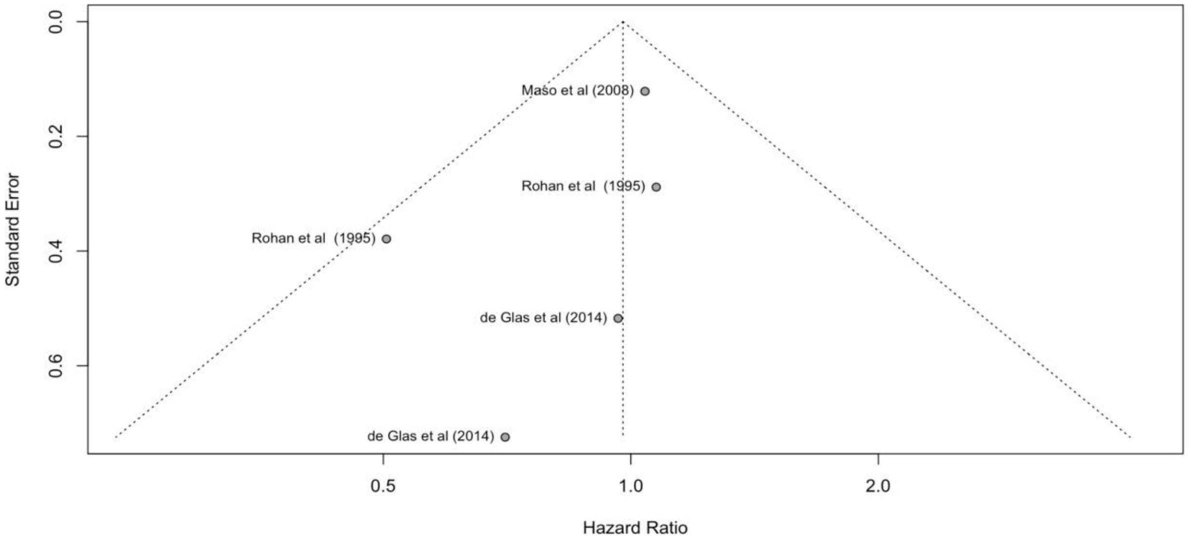

Funnel plot (Physical ac(vity – high to vigorous)

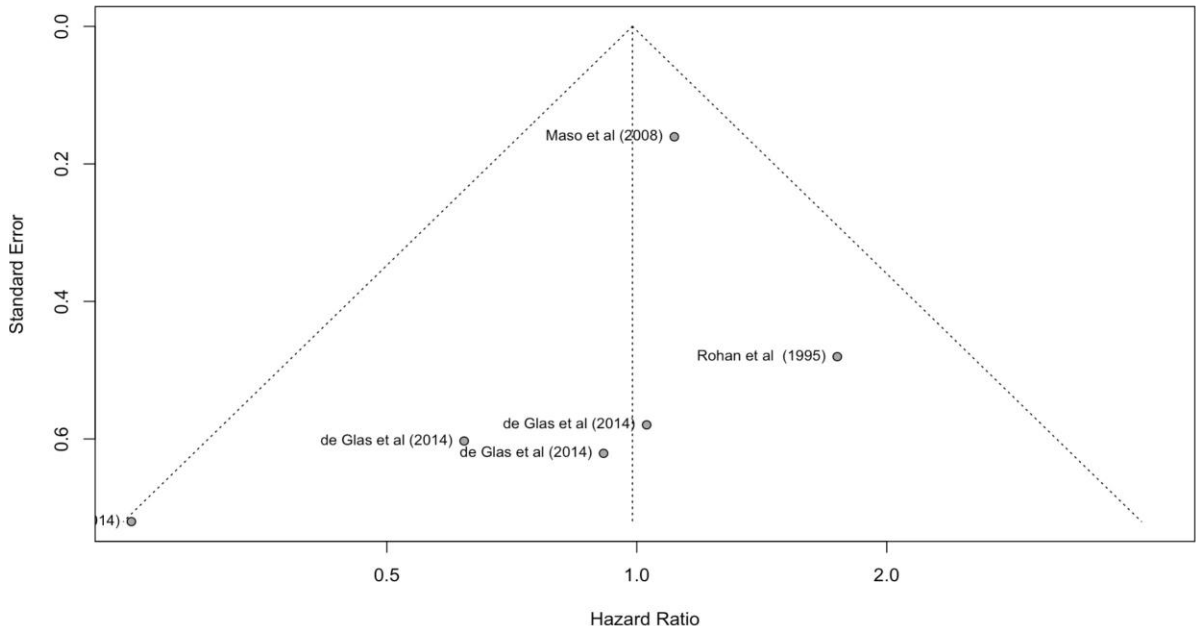

Funnel plot (Body Mass Index – Overweight to Obese)

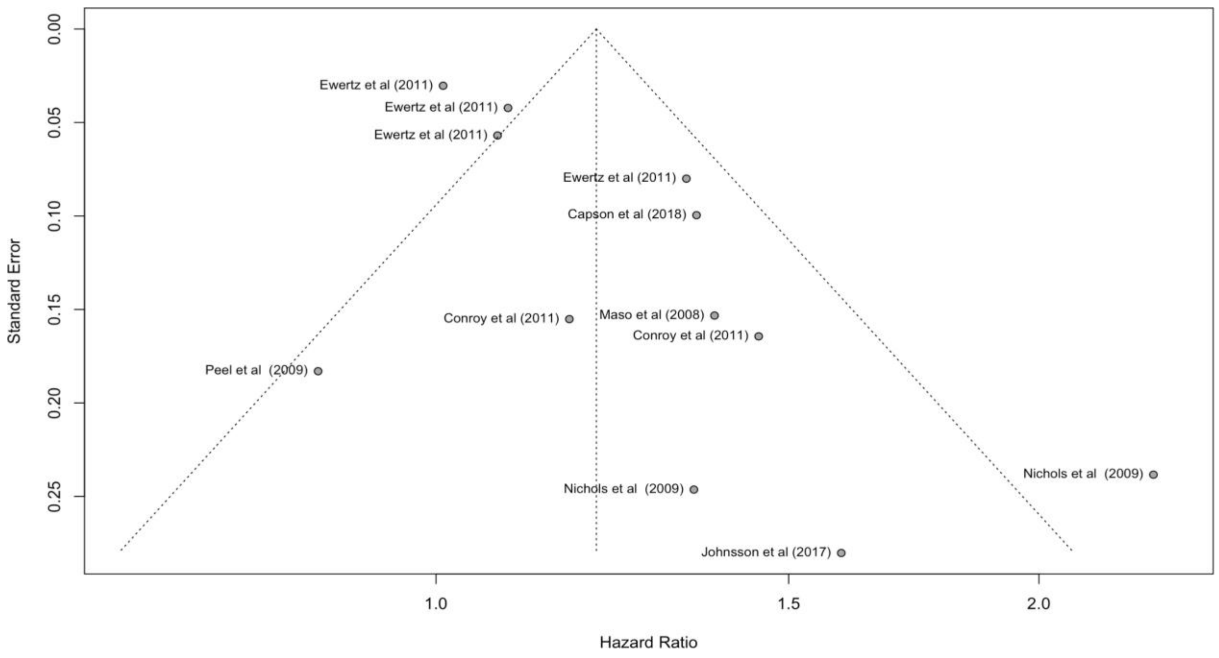

Funnel plot (BRCA 1)

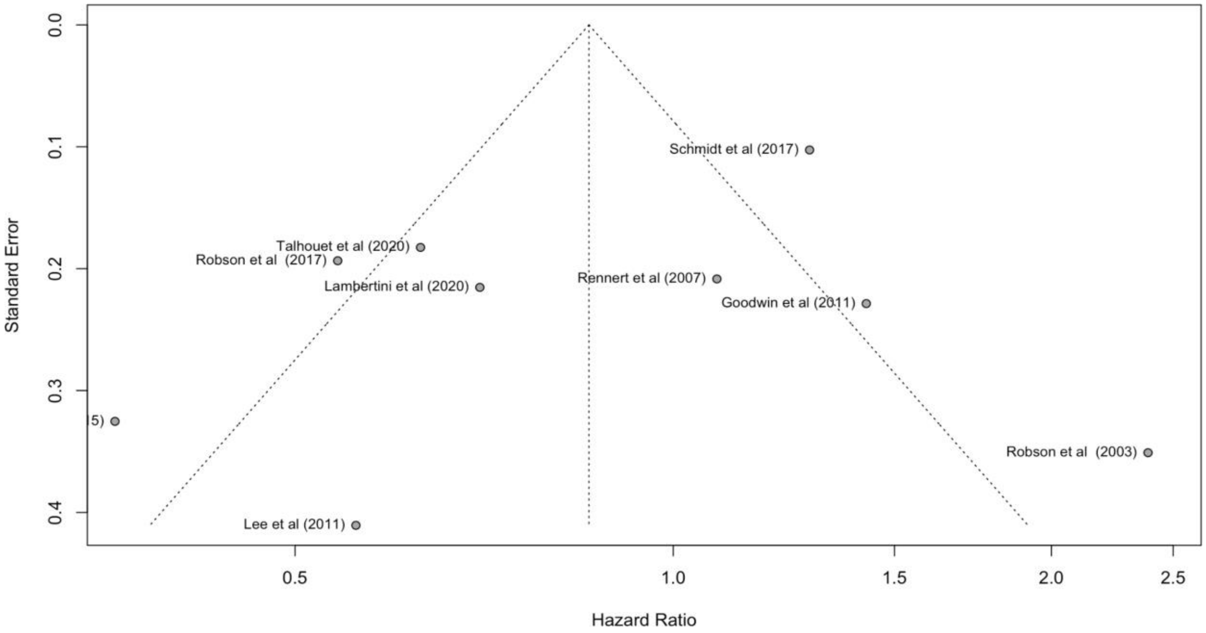

Funnel plot (BRCA 2)

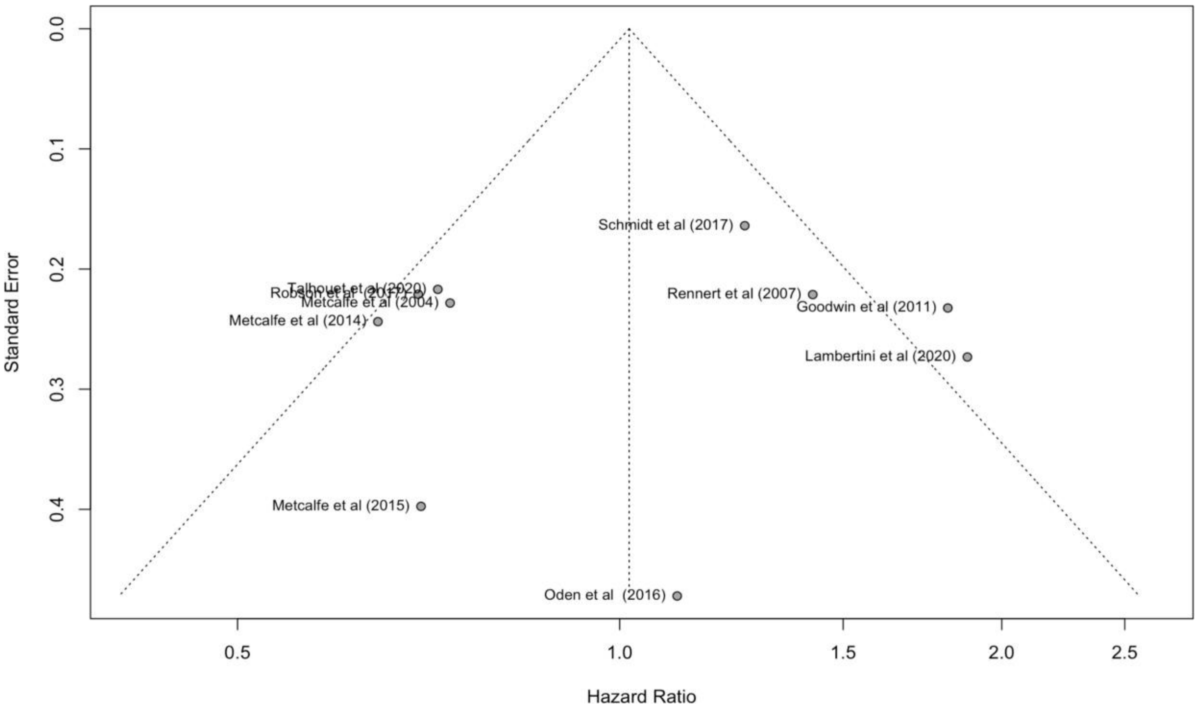

Funnel plot (Oral contracep(ve use)

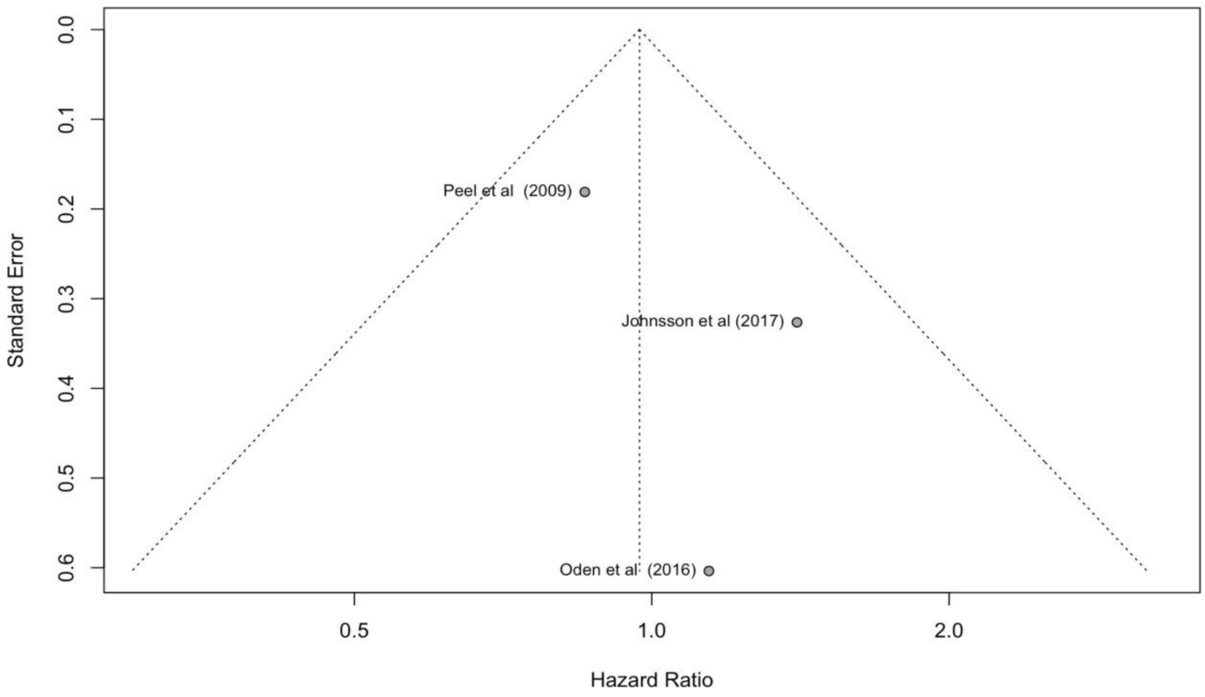

Funnel plot (comorbidity index – 1 to 2)

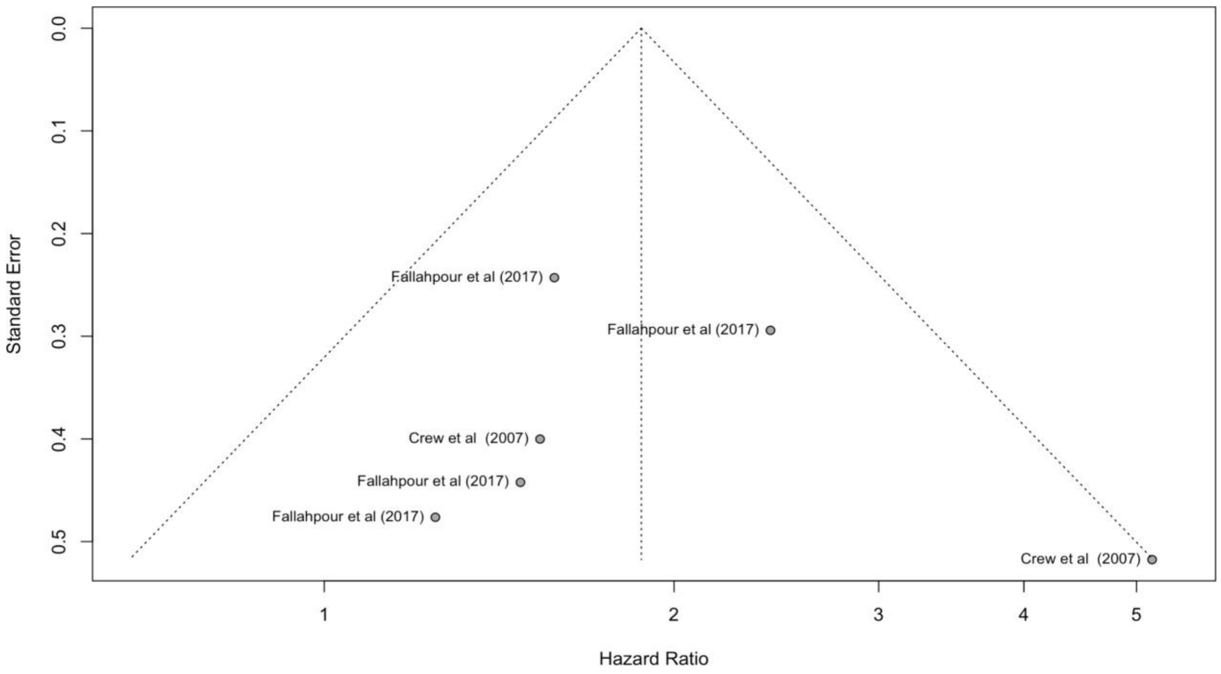

Funnel plot (comorbidity index ≥ 3)

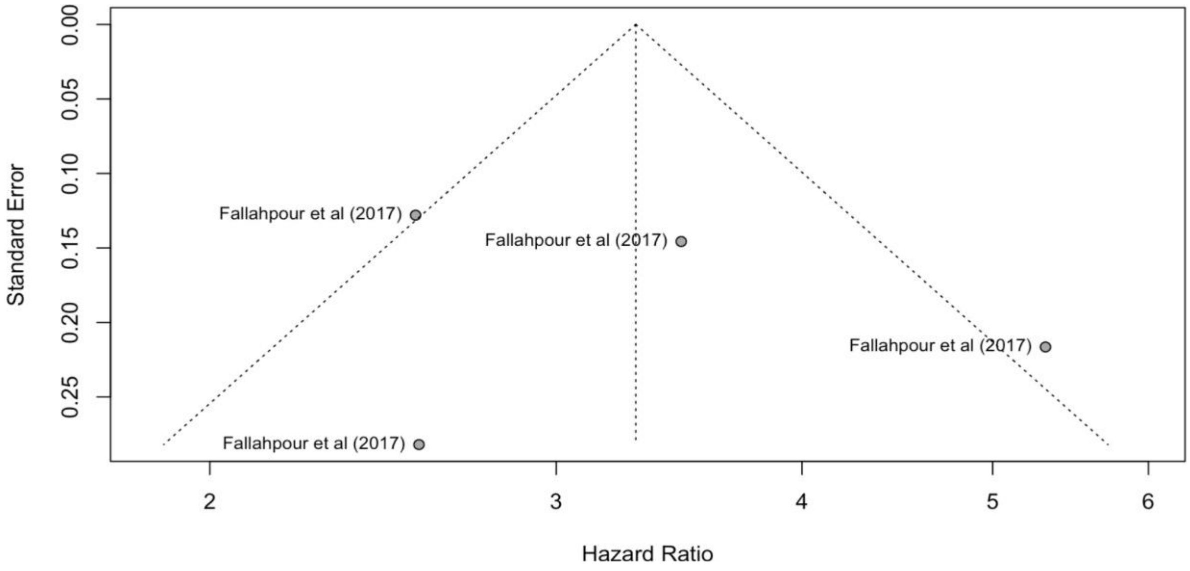

Supplement: Supplementary file 1 [file diseases-12-00111-s001.zip › diseases-2980345-Supplementary File S2.pdf]
