# Supplementary material for: Prognostic Factors Associated with Breast Cancer-Specific Survival from 1995 to 2022: A Systematic Review and Meta-Analysis of 1,386,663 Cases from 30 Countries
Source: Diseases. 2024 May 23;12(6):111. doi: 10.3390/diseases12060111 (PMC11203054; doi:10.3390/diseases12060111)

Description of Supplementary file 1

This file contain visualizations of Forest plots generated for each factor other than the Top-5 factors that worsen and improve breast-cancer specific survival, which is reported in the main text.

Figure S1: Forest plot for Random-effects Hazard Ratio Model of Age (Below 35)

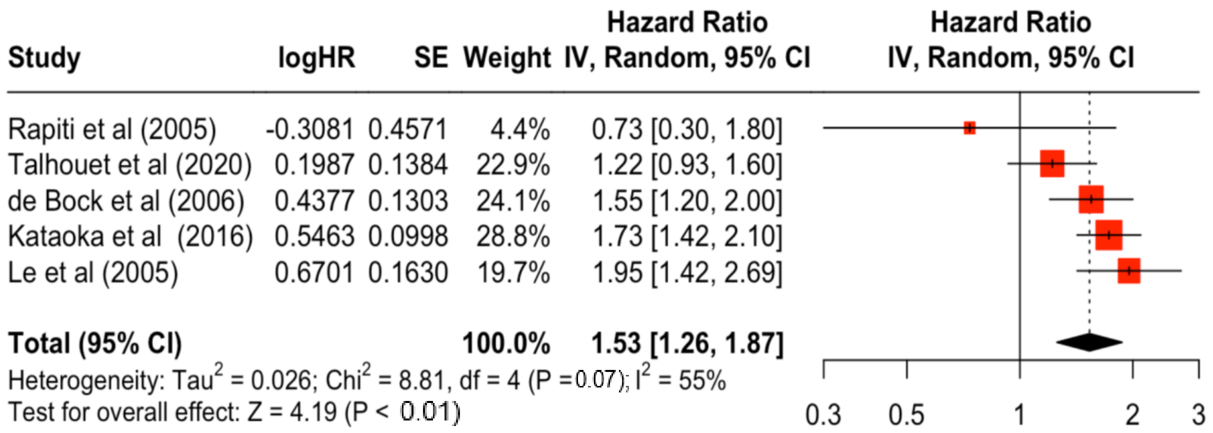

Figure S2: Forest plot for Random-effects Hazard Ratio Model of Age (35 to 60)

Study	logHR	SE	Weight	Hazard Ratio IV, Random, 95% CI
Metcalfe et al (2015)	-0.7543	0.2646	1.5%	0.47 [0.28, 0.79]
Ali et al (2014)	-0.4323	0.0938	2.7%	0.65 [0.54, 0.78]
Robson et al (2017)	-0.4206	0.1494	2.3%	0.66 [0.49, 0.88]
Movahedi et al (2012)	-0.3966	0.0756	2.8%	0.67 [0.58, 0.78]
Partridge et al (2016)	-0.3466	0.1768	2.1%	0.71 [0.50, 1.00]
Fallahpour et al (2017)	-0.2554	0.2233	1.8%	0.77 [0.50, 1.20]
Peel et al (2009)	-0.2093	0.0752	2.8%	0.81 [0.70, 0.94]
Eng et al (2016)	-0.2061	0.0282	3.0%	0.81 [0.77, 0.86]
Eng et al (2016)	-0.1991	0.0187	3.1%	0.82 [0.79, 0.85]
Huzarski et al (2013)	-0.1756	0.0997	2.7%	0.84 [0.69, 1.02]
Hwang et al (2013)	-0.1408	0.0615	2.9%	0.87 [0.77, 0.98]
Kim et al (2022)	-0.1093	0.0455	3.0%	0.90 [0.82, 0.98]
Movahedi et al (2012)	-0.0594	0.0835	2.8%	0.94 [0.80, 1.11]
Ali et al (2014)	-0.0479	0.1089	2.6%	0.95 [0.77, 1.18]
Rapiti et al (2005)	-0.0472	0.1579	2.2%	0.95 [0.70, 1.30]
Vostakolaei et al (2012)	0.0363	0.2462	1.6%	1.04 [0.64, 1.68]
Vostakolaei et al (2012)	0.0363	0.3143	1.2%	1.04 [0.56, 1.92]
de Bock et al (2006)	0.0046	0.0734	2.9%	1.00 [0.87, 1.16]
Le et al (2005)	0.0157	0.0850	2.8%	1.02 [0.86, 1.20]
Hwang et al (2013)	0.0271	0.0619	2.9%	1.03 [0.91, 1.16]
Ali et al (2014)	0.0355	0.0833	2.8%	1.04 [0.88, 1.22]
Colzani et al (2011)	0.1682	0.0858	2.8%	1.18 [1.00, 1.40]
Partridge et al (2016)	0.0385	0.0734	2.9%	1.04 [0.90, 1.20]
Hussain et al (2008)	0.1190	0.0407	3.0%	1.13 [1.04, 1.22]
Kim et al (2022)	0.1272	0.0449	3.0%	1.14 [1.04, 1.24]
Ali et al (2014)	0.1852	0.0844	2.8%	1.20 [1.02, 1.42]
Colzani et al (2011)	0.1682	0.0858	2.8%	1.18 [1.00, 1.40]
Fallahpour et al (2017)	0.2122	0.4041	0.9%	1.24 [0.56, 2.73]
Maso et al (2008)	0.2208	0.1335	2.4%	1.25 [0.96, 1.62]
Maso et al (2008)	0.2373	0.1526	2.3%	1.27 [0.94, 1.71]
Le et al (2005)	0.2562	0.0962	2.7%	1.29 [1.07, 1.56]
Kim et al (2022)	0.2764	0.0924	2.7%	1.32 [1.10, 1.58]
Fallahpour et al (2017)	0.2844	0.2162	1.8%	1.33 [0.87, 2.03]
Kim et al (2022)	0.2947	0.0531	3.0%	1.34 [1.21, 1.49]
Hussain et al (2008)	0.3238	0.0314	3.0%	1.38 [1.30, 1.47]
Fallahpour et al (2017)	0.4372	0.3907	0.9%	1.55 [0.72, 3.33]
Fallahpour et al (2017)	0.5700	0.2064	1.9%	1.77 [1.18, 2.65]
Partridge et al (2016)	0.6515	0.0926	2.7%	1.92 [1.60, 2.30]
Wong et al (2018)	0.6897	0.2463	1.6%	1.99 [1.23, 3.23]
Fallahpour et al (2017)	1.4183	0.1965	1.9%	4.13 [2.81, 6.07]
Fallahpour et al (2017)	1.7306	0.3433	1.1%	5.64 [2.88, 11.06]
Fallahpour et al (2017)	1.8859	0.2975	1.3%	6.59 [3.68, 11.81]
**Total (95% CI)**			**100.0%**	**1.11 [1.01, 1.21]**

Heterogeneity: $Tau^2 = 0.065$; $Chi^2 = 629.27$, df = 41 ($P < 0.01$); $I^2 = 93\%$

Test for overall effect: Z = 2.27 (P = 0.02)

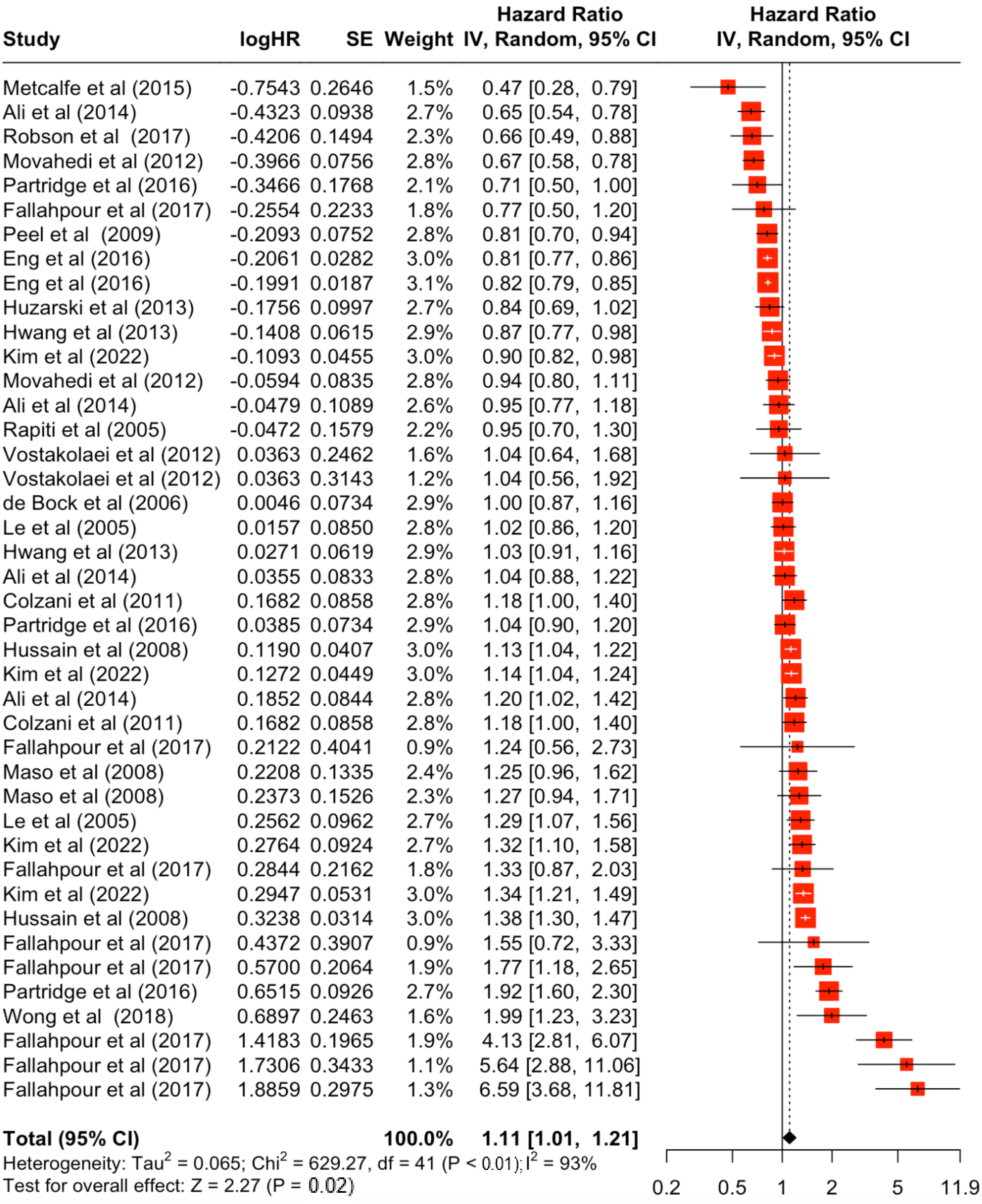

Figure S3: Forest plot for Random-effects Hazard Ratio Model of Age (Above 60)

Study	logHR	SE	Weight	Hazard Ratio IV, Random, 95% CI
Metcalfe et al (2015)	-0.6464	0.6381	1.3%	0.52 [0.15, 1.83]
Robson et al (2003)	-0.5960	0.2616	3.0%	0.55 [0.33, 0.92]
Partridge et al (2016)	-0.3081	0.1034	3.8%	0.73 [0.60, 0.90]
Peel et al (2009)	-0.3222	0.1893	3.4%	0.72 [0.50, 1.05]
Fallahpour et al (2017)	-0.3272	0.2293	3.1%	0.72 [0.46, 1.13]
Kim et al (2022)	-0.0179	0.1585	3.5%	0.98 [0.72, 1.34]
Conroy et al (2011)	-0.0262	0.1472	3.6%	0.97 [0.73, 1.30]
Fallahpour et al (2017)	0.0465	0.4887	1.8%	1.05 [0.40, 2.73]
Hwang et al (2013)	0.1323	0.0624	3.9%	1.14 [1.01, 1.29]
Conroy et al (2011)	0.1352	0.1579	3.5%	1.14 [0.84, 1.56]
Kim et al (2022)	0.1818	0.0679	3.9%	1.20 [1.05, 1.37]
Hwang et al (2013)	0.2320	0.0605	3.9%	1.26 [1.12, 1.42]
Kim et al (2022)	0.2399	0.0601	3.9%	1.27 [1.13, 1.43]
Movahedi et al (2012)	0.2726	0.0812	3.8%	1.31 [1.12, 1.54]
Hwang et al (2013)	0.2963	0.0624	3.9%	1.34 [1.19, 1.52]
Colzani et al (2011)	0.3565	0.0889	3.8%	1.43 [1.20, 1.70]
Maso et al (2008)	0.3835	0.1424	3.6%	1.47 [1.11, 1.94]
Hwang et al (2013)	0.5029	0.0600	3.9%	1.65 [1.47, 1.86]
Fallahpour et al (2017)	0.5488	0.2268	3.2%	1.73 [1.11, 2.70]
Kim et al (2022)	0.5939	0.0506	3.9%	1.81 [1.64, 2.00]
Fallahpour et al (2017)	0.5882	0.2029	3.3%	1.80 [1.21, 2.68]
Fallahpour et al (2017)	0.5995	0.3924	2.3%	1.82 [0.84, 3.93]
Fallahpour et al (2017)	0.5341	0.3320	2.6%	1.71 [0.89, 3.27]
Fallahpour et al (2017)	0.6113	0.4452	2.0%	1.84 [0.77, 4.41]
Crew et al (2007)	0.8045	0.6148	1.4%	2.24 [0.67, 7.46]
Fallahpour et al (2017)	0.8292	0.2028	3.3%	2.29 [1.54, 3.41]
Crew et al (2007)	0.8786	0.6159	1.4%	2.41 [0.72, 8.05]
Kim et al (2022)	0.8950	0.1056	3.7%	2.45 [1.99, 3.01]
Kim et al (2022)	1.0058	0.0457	3.9%	2.73 [2.50, 2.99]
Kim et al (2022)	1.3216	0.0954	3.8%	3.75 [3.11, 4.52]
Kim et al (2022)	1.3312	0.0518	3.9%	3.79 [3.42, 4.19]
**Total (95% CI)**			**100.0%**	**1.45 [1.21, 1.72]**

Heterogeneity: $Tau^2 = 0.204$; $Chi^2 = 731.92$, df = 30 ($P < 0.01$); $I^2 = 96\%$
Test for overall effect: $Z = 4.11$ ($P < 0.01$)

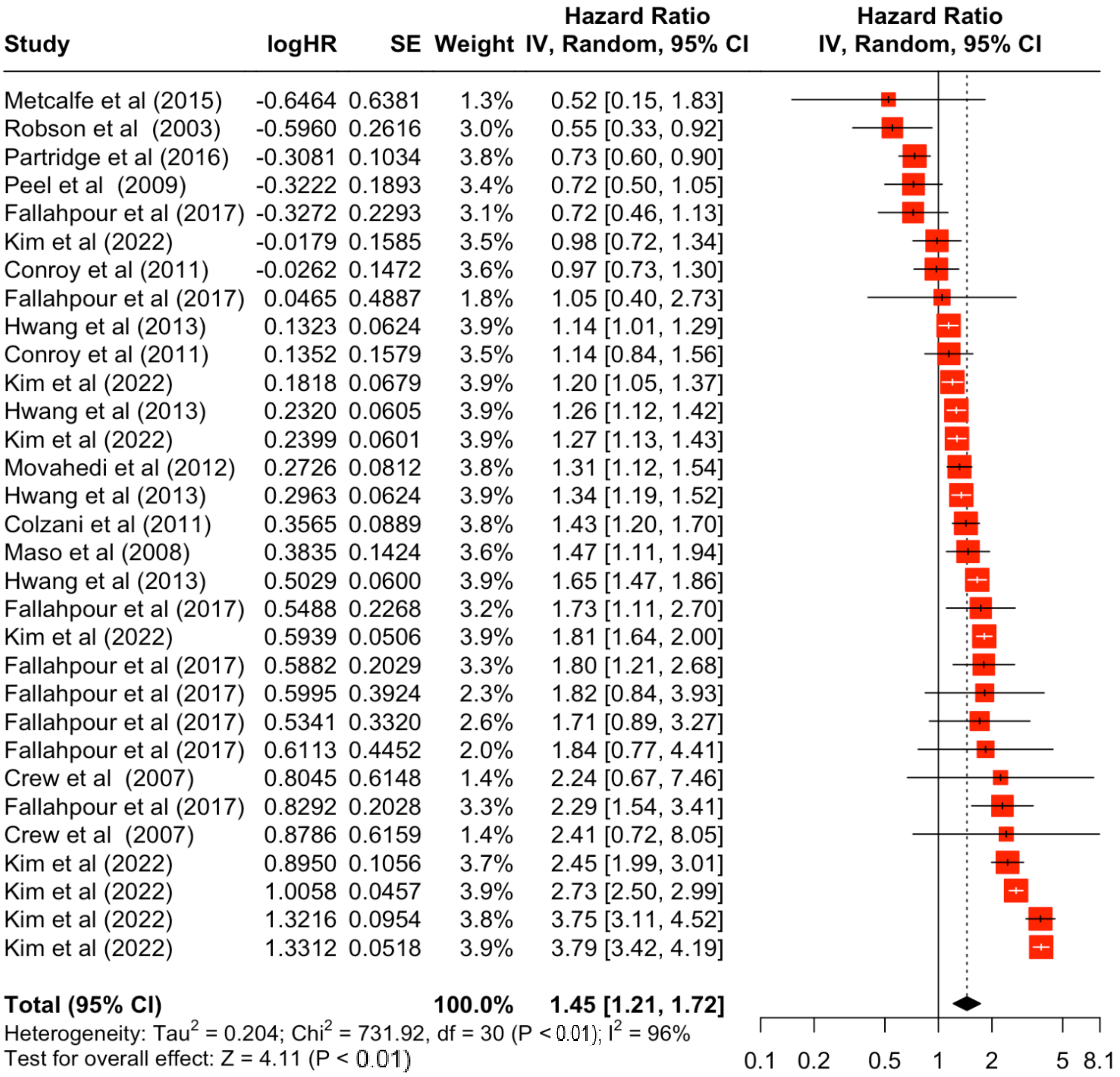

Figure S4: Forest plot for Random-effects Hazard Ratio Model of Race (black)

Study	logHR	SE	Weight	Hazard Ratio IV, Random, 95% CI
Le et al (2005)	-0.1077	0.2230	1.0%	0.90 [0.58, 1.39]
Hwang et al (2013)	0.1707	0.0622	9.3%	1.19 [1.05, 1.34]
Sohn et al (2008)	0.2998	0.0720	7.6%	1.35 [1.17, 1.55]
Keegan et al (2013)	0.2979	0.1728	1.6%	1.35 [0.96, 1.89]
Eng et al (2016)	0.3321	0.0201	24.8%	1.39 [1.34, 1.45]
Field et al (2005)	0.3295	0.0422	15.0%	1.39 [1.28, 1.51]
Vinh-Hung et al (2002)	0.3486	0.0324	19.0%	1.42 [1.33, 1.51]
Conroy et al (2011)	0.3652	0.1518	2.1%	1.44 [1.07, 1.94]
Capson et al (2018)	0.3976	0.1683	1.7%	1.49 [1.07, 2.07]
Chagpar et al (2017)	0.4053	0.0425	14.9%	1.50 [1.38, 1.63]
Wray et al (2013)	0.4310	0.1312	2.7%	1.54 [1.19, 1.99]
Crew et al (2007)	1.1919	0.5595	0.2%	3.29 [1.10, 9.86]
**Total (95% CI)**			**100.0%**	**1.39 [1.33, 1.45]**

Heterogeneity: $Tau^2 = 0.002$; $Chi^2 = 17.21$, df = 11 (P = 0.10); $I^2 = 36\%$

Test for overall effect: Z = 14.45 (P < 0.01)

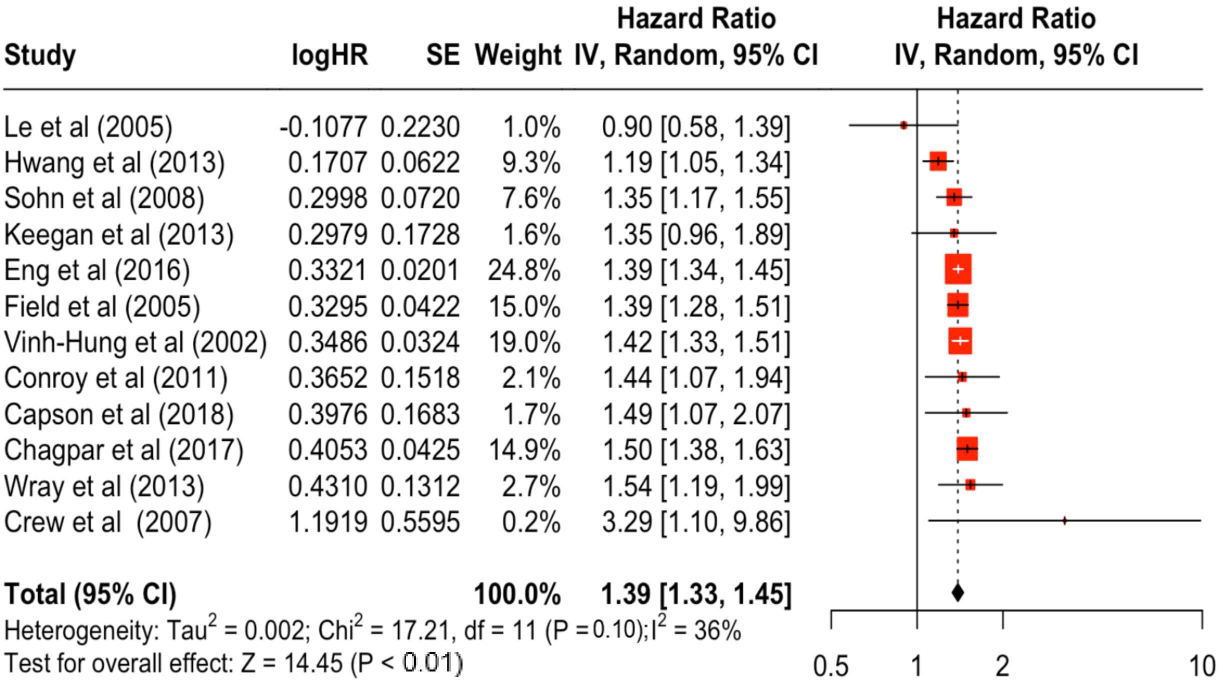

Figure S5: Forest plot for Random-effects Hazard Ratio Model of Race (Asian)

Study	logHR	SE	Weight	Hazard Ratio IV, Random, 95% CI
Hwang et al (2013)	-0.3464	0.0755	23.3%	0.71 [0.61, 0.82]
Conroy et al (2011)	-0.2514	0.1675	8.2%	0.78 [0.56, 1.08]
Sohn et al (2008)	-0.1562	0.0972	17.8%	0.86 [0.71, 1.03]
Le et al (2005)	-0.1460	0.2123	5.5%	0.86 [0.57, 1.31]
Eng et al (2016)	-0.1136	0.0371	36.1%	0.89 [0.83, 0.96]
Wray et al (2013)	0.0422	0.2492	4.1%	1.04 [0.64, 1.70]
Capson et al (2018)	0.0870	0.2264	4.9%	1.09 [0.70, 1.70]
**Total (95% CI)**			**100.0%**	**0.84 [0.76, 0.93]**

Heterogeneity: $Tau^2 = 0.006$; $Chi^2 = 9.78$, df = 6 (P = 0.13); $I^2 = 39\%$

Test for overall effect: Z = -3.23 (P < 0.01)

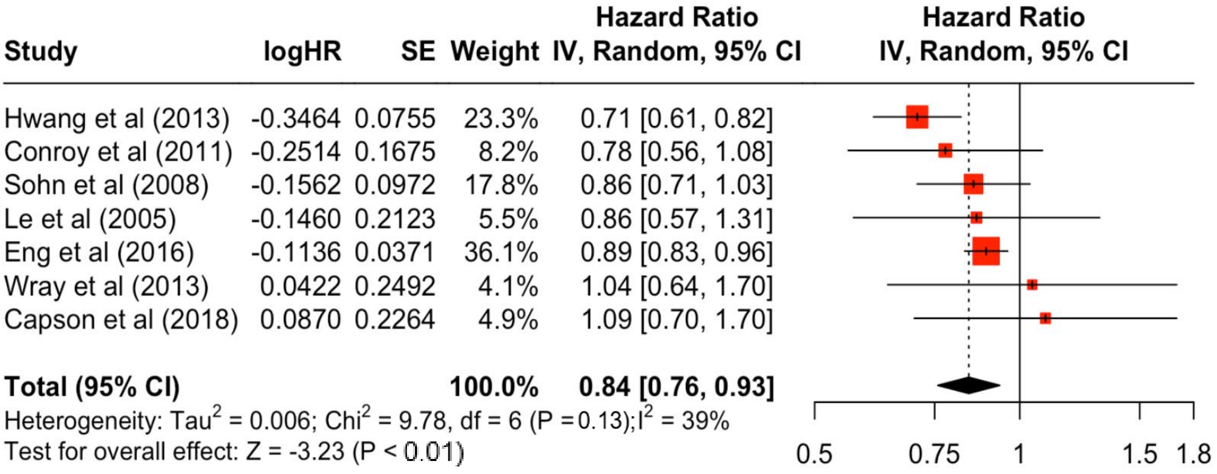

Figure S6: Forest plot for Random-effects Hazard Ratio Model of Race (Hispanic)

Study	logHR	SE	Weight	Hazard Ratio IV, Random, 95% CI
Sohn et al (2008)	-0.0678	0.1373	14.7%	0.93 [0.71, 1.22]
Hwang et al (2013)	-0.0050	0.0512	22.4%	0.99 [0.90, 1.10]
Chagpar et al (2017)	0.0529	0.0531	22.3%	1.05 [0.95, 1.17]
Keegan et al (2013)	0.1138	0.1350	14.9%	1.12 [0.86, 1.46]
Le et al (2005)	0.3202	0.2004	10.2%	1.38 [0.93, 2.04]
Wray et al (2013)	0.6180	0.1292	15.4%	1.86 [1.44, 2.39]
**Total (95% CI)**			**100.0%**	**1.16 [0.98, 1.36]**

Heterogeneity: $Tau^2 = 0.029$; $Chi^2 = 22.90$, df = 5 (P < 0.01); $I^2 = 78\%$
Test for overall effect: Z = 1.74 (P = 0.08)

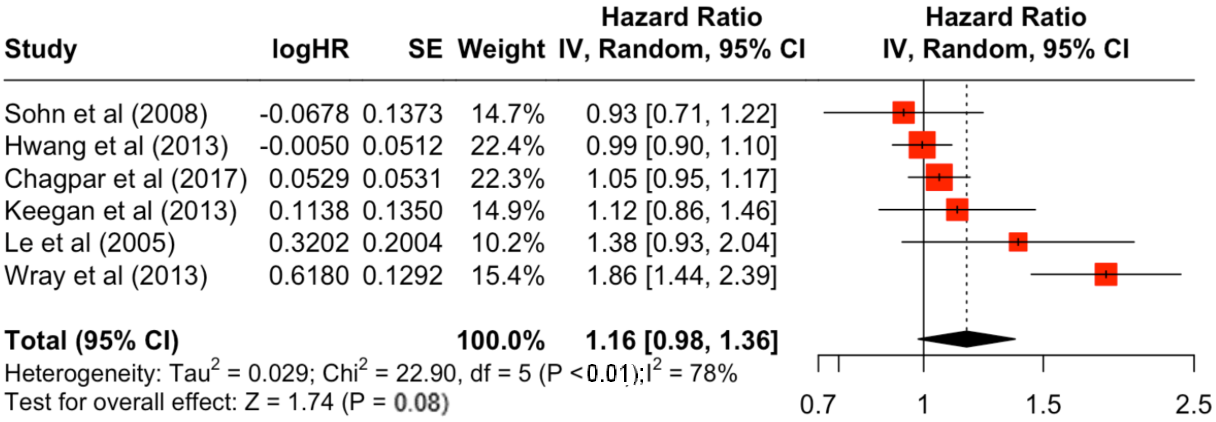

Figure S7: Forest plot for Random-effects Hazard Ratio Model of Grade 2

Study	logHR	SE	Weight	Hazard Ratio IV, Random, 95% CI
Ali et al (2014)	-0.1477	0.1768	8.2%	0.86 [0.61, 1.22]
Giordano (2004)	0.0589	0.0838	10.4%	1.06 [0.90, 1.25]
Ali et al (2014)	0.0767	0.0986	10.1%	1.08 [0.89, 1.31]
Schmidt et al (2017)	0.2954	0.0663	10.7%	1.34 [1.18, 1.53]
Sohn et al (2008)	0.2936	0.0598	10.8%	1.34 [1.19, 1.51]
Talhouet et al (2020)	0.3594	0.3234	5.0%	1.43 [0.76, 2.70]
Saadatmand et al (2015)	0.3552	0.0357	11.1%	1.43 [1.33, 1.53]
Chagpar et al (2017)	0.7855	0.0395	11.1%	2.19 [2.03, 2.37]
Vostakolaei et al (2012)	0.7869	0.6135	2.1%	2.20 [0.66, 7.31]
Hwang et al (2013)	0.8313	0.1048	10.0%	2.30 [1.87, 2.82]
Capson et al (2018)	0.9316	0.3110	5.2%	2.54 [1.38, 4.67]
Kaffashian et al (2003)	1.4053	0.3055	5.3%	4.08 [2.24, 7.42]
**Total (95% CI)**			**100.0%**	**1.54 [1.27, 1.87]**

Heterogeneity: $Tau^2 = 0.084$; $Chi^2 = 164.76$, df = 11 (P <0.01); $I^2 = 93\%$
Test for overall effect: Z = 4.44 (P < 0.01)

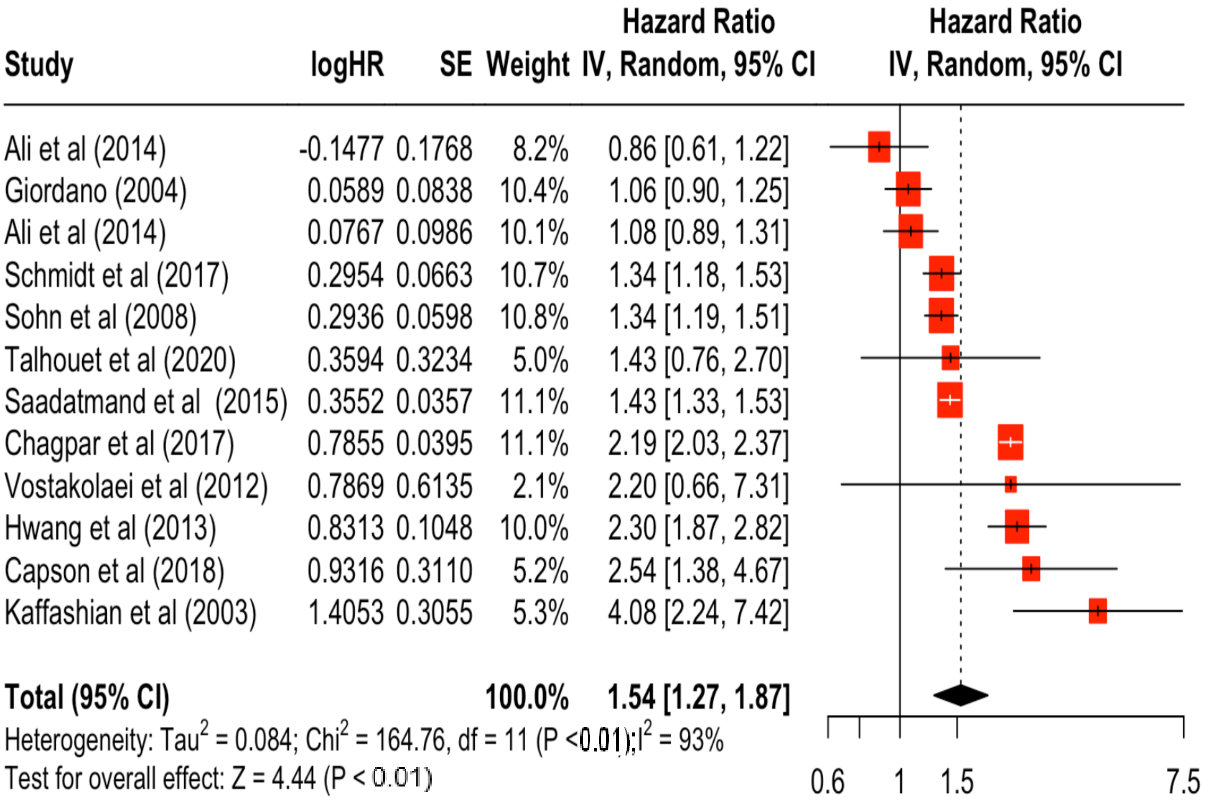

Figure S8: Forest plot for Random-effects Hazard Ratio Model of Grade 3

Study	logHR	SE	Weight	Hazard Ratio IV, Random, 95% CI
Ali et al (2014)	-0.3839	0.0820	8.4%	0.68 [0.58, 0.80]
Ali et al (2014)	-0.0532	0.0804	8.4%	0.95 [0.81, 1.11]
Giordano (2004)	0.1547	0.0349	8.5%	1.17 [1.09, 1.25]
Talhouet et al (2020)	0.1711	0.3150	6.9%	1.19 [0.64, 2.20]
Vollebergh et al (2014)	0.2889	0.2549	7.4%	1.33 [0.81, 2.20]
Kim EK et al (2011)	0.3845	0.0864	8.4%	1.47 [1.24, 1.74]
Sohn et al (2008)	0.5241	0.0757	8.4%	1.69 [1.46, 1.96]
Schmidt et al (2017)	0.5203	0.0620	8.4%	1.68 [1.49, 1.90]
Saadatmand et al (2015)	0.9140	0.0347	8.5%	2.49 [2.33, 2.67]
Capson et al (2018)	1.2052	0.3094	6.9%	3.34 [1.82, 6.12]
Chagpar et al (2017)	1.5831	0.0403	8.5%	4.87 [4.50, 5.27]
Vostakolaei et al (2012)	1.9409	0.6117	4.5%	6.96 [2.10, 23.10]
Kaffashian et al (2003)	1.9837	0.2997	7.0%	7.27 [4.04, 13.08]
**Total (95% CI)**			**100.0%**	**1.92 [1.33, 2.76]**

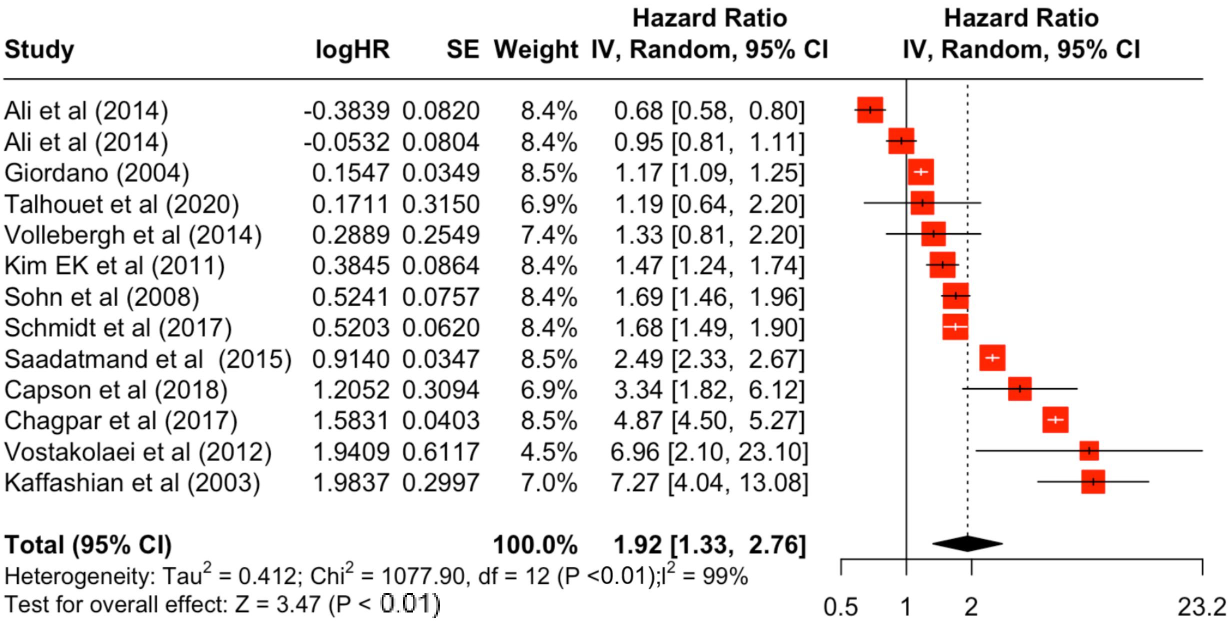

Heterogeneity: $Tau^2 = 0.412$; $Chi^2 = 1077.90$, df = 12 (P <0.01);$I^2 = 99\%$
Test for overall effect: Z = 3.47 (P < 0.01)

Figure S9: Forest plot for Random-effects Hazard Ratio Model of Stage 2

Study	logHR	SE	Weight	Hazard Ratio IV, Random, 95% CI
Metcalfe et al (2015)	-0.6892	0.2979	6.7%	0.50 [0.28, 0.90]
Lan et al (2013)	0.2173	0.1160	9.3%	1.24 [0.99, 1.56]
Gnerlich et al (2009)	0.2178	0.0267	10.0%	1.24 [1.18, 1.31]
Sohn et al (2008)	0.5217	0.0559	9.8%	1.68 [1.51, 1.88]
Vostakolaei et al (2012)	0.6956	0.7086	2.7%	2.00 [0.50, 8.04]
Maso et al (2008)	0.7045	0.1261	9.2%	2.02 [1.58, 2.59]
Kataoka et al (2016)	-0.0096	0.0489	9.9%	0.99 [0.90, 1.09]
Kaffashian et al (2003)	0.8750	0.0752	9.7%	2.40 [2.07, 2.78]
Maso et al (2008)	0.9231	0.1489	8.9%	2.52 [1.88, 3.37]
Rapiti et al (2005)	1.0061	0.4647	4.6%	2.73 [1.10, 6.80]
Saadatmand et al (2015)	1.2663	0.0738	9.7%	3.55 [3.07, 4.10]
Rapiti et al (2005)	1.8829	0.4539	4.7%	6.57 [2.70, 16.00]
Crew et al (2007)	1.9835	0.5983	3.4%	7.27 [2.25, 23.48]
Johnsson et al (2017)	2.4029	1.0191	1.5%	11.06 [1.50, 81.48]
**Total (95% CI)**			**100.0%**	**1.93 [1.48, 2.51]**

Heterogeneity: $Tau^2 = 0.183$; $Chi^2 = 352.18$, df = 13 (P <0.01); $I^2 = 96\%$
Test for overall effect: Z = 4.86 (P < 0.01)

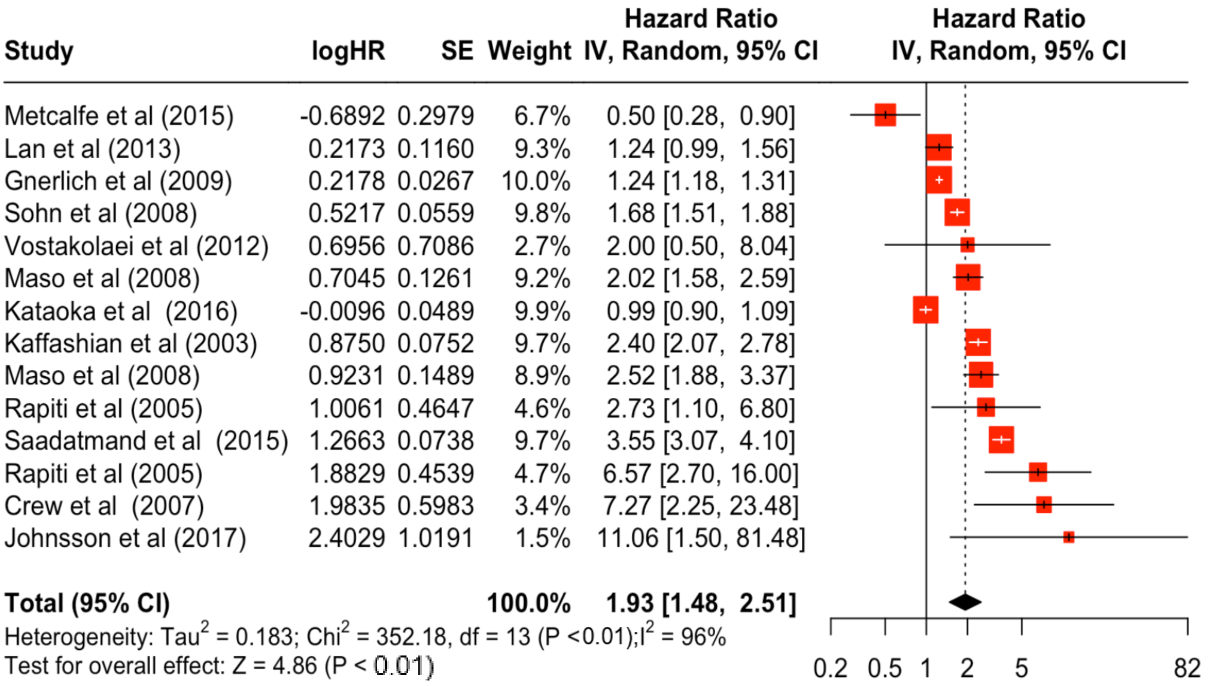

Figure S10: Forest plot for Random-effects Hazard Ratio Model of Differentiation (Moderate)

Study	logHR	SE	Weight	Hazard Ratio IV, Random, 95% CI
Field et al (2005)	0.1303	0.0514	38.5%	1.14 [1.03, 1.26]
Vinh-Hung et al (2002)	0.2173	0.0471	38.9%	1.24 [1.13, 1.36]
Le et al (2005)	0.7702	0.3306	11.5%	2.16 [1.13, 4.13]
Rapiti et al (2005)	1.5785	0.3379	11.1%	4.85 [2.50, 9.40]
**Total (95% CI)**			**100.0%**	**1.49 [1.15, 1.93]**

Heterogeneity: $Tau^2 = 0.042$; $Chi^2 = 21.59$, df = 3 (P <0.01); $I^2 = 86\%$

Test for overall effect: Z = 3.02 (P < 0.01)

Hazard Ratio
IV, Random, 95% CI

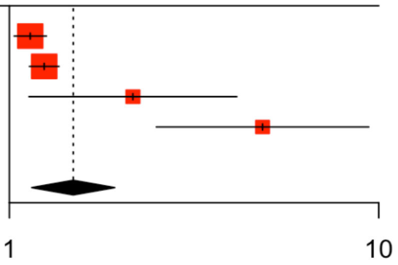

Figure S11: Forest plot for Random-effects Hazard Ratio Model of Positive Nodes

Study	logHR	SE	Weight	Hazard Ratio IV, Random, 95% CI
Ali et al (2014)	-0.4192	0.1823	2.9%	0.66 [0.46, 0.94]
Ali et al (2014)	-0.3123	0.1842	2.9%	0.73 [0.51, 1.05]
Ali et al (2014)	-0.3081	0.1572	3.0%	0.73 [0.54, 1.00]
de Bock et al (2006)	-0.1379	0.2347	2.7%	0.87 [0.55, 1.38]
Ali et al (2014)	-0.1093	0.2311	2.8%	0.90 [0.57, 1.41]
Ali et al (2014)	-0.0806	0.1866	2.9%	0.92 [0.64, 1.33]
Saadatmand et al (2015)	0.0087	0.0253	3.3%	1.01 [0.96, 1.06]
Ali et al (2014)	0.0281	0.1156	3.2%	1.03 [0.82, 1.29]
Ali et al (2014)	0.0208	0.1574	3.0%	1.02 [0.75, 1.39]
Eng et al (2016)	0.0227	0.0324	3.3%	1.02 [0.96, 1.09]
Ali et al (2014)	0.0645	0.1532	3.0%	1.07 [0.79, 1.44]
Keegan et al (2013)	0.2182	0.1594	3.0%	1.24 [0.91, 1.70]
Eng et al (2016)	0.2913	0.0267	3.3%	1.34 [1.27, 1.41]
Giordano (2004)	0.3490	0.0521	3.3%	1.42 [1.28, 1.57]
Talhouet et al (2020)	0.3686	0.1394	3.1%	1.45 [1.10, 1.90]
Saadatmand et al (2015)	0.5106	0.0337	3.3%	1.67 [1.56, 1.78]
Valentini et al (2013)	0.5139	0.6057	1.4%	1.67 [0.51, 5.48]
Metcalfe et al (2014)	0.5304	0.2266	2.8%	1.70 [1.09, 2.65]
Schmidt et al (2017)	0.5470	0.0457	3.3%	1.73 [1.58, 1.89]
Robson et al (2003)	0.5764	0.2596	2.6%	1.78 [1.07, 2.96]
Hwang et al (2013)	0.6111	0.0594	3.3%	1.84 [1.64, 2.07]
de Bock et al (2006)	0.7488	0.0793	3.2%	2.11 [1.81, 2.47]
Colzani et al (2011)	0.8789	0.0948	3.2%	2.41 [2.00, 2.90]
Hwang et al (2013)	0.8968	0.0530	3.3%	2.45 [2.21, 2.72]
Schmidt et al (2017)	0.9139	0.0663	3.3%	2.49 [2.19, 2.84]
Kataoka et al (2016)	0.7991	0.0515	3.3%	2.22 [2.01, 2.46]
Saadatmand et al (2015)	1.1576	0.0352	3.3%	3.18 [2.97, 3.41]
Huzarski et al (2013)	1.2518	0.1008	3.2%	3.50 [2.87, 4.26]
Kim EK et al (2011)	1.2931	0.0809	3.2%	3.64 [3.11, 4.27]
Schmidt et al (2017)	1.3464	0.0596	3.3%	3.84 [3.42, 4.32]
Hwang et al (2013)	1.4536	0.0447	3.3%	4.28 [3.92, 4.67]
Colzani et al (2011)	1.7747	0.0946	3.2%	5.90 [4.90, 7.10]
Lee et al (2011)	1.9420	0.5033	1.7%	6.97 [2.60, 18.70]
**Total (95% CI)**			**100.0%**	**1.71 [1.42, 2.05]**

Heterogeneity: Tau^2 = 0.259; Chi^2 = 2295.30, df = 32 (P = 0); I^2 = 99%
Test for overall effect: Z = 5.77 (P < 0.01)

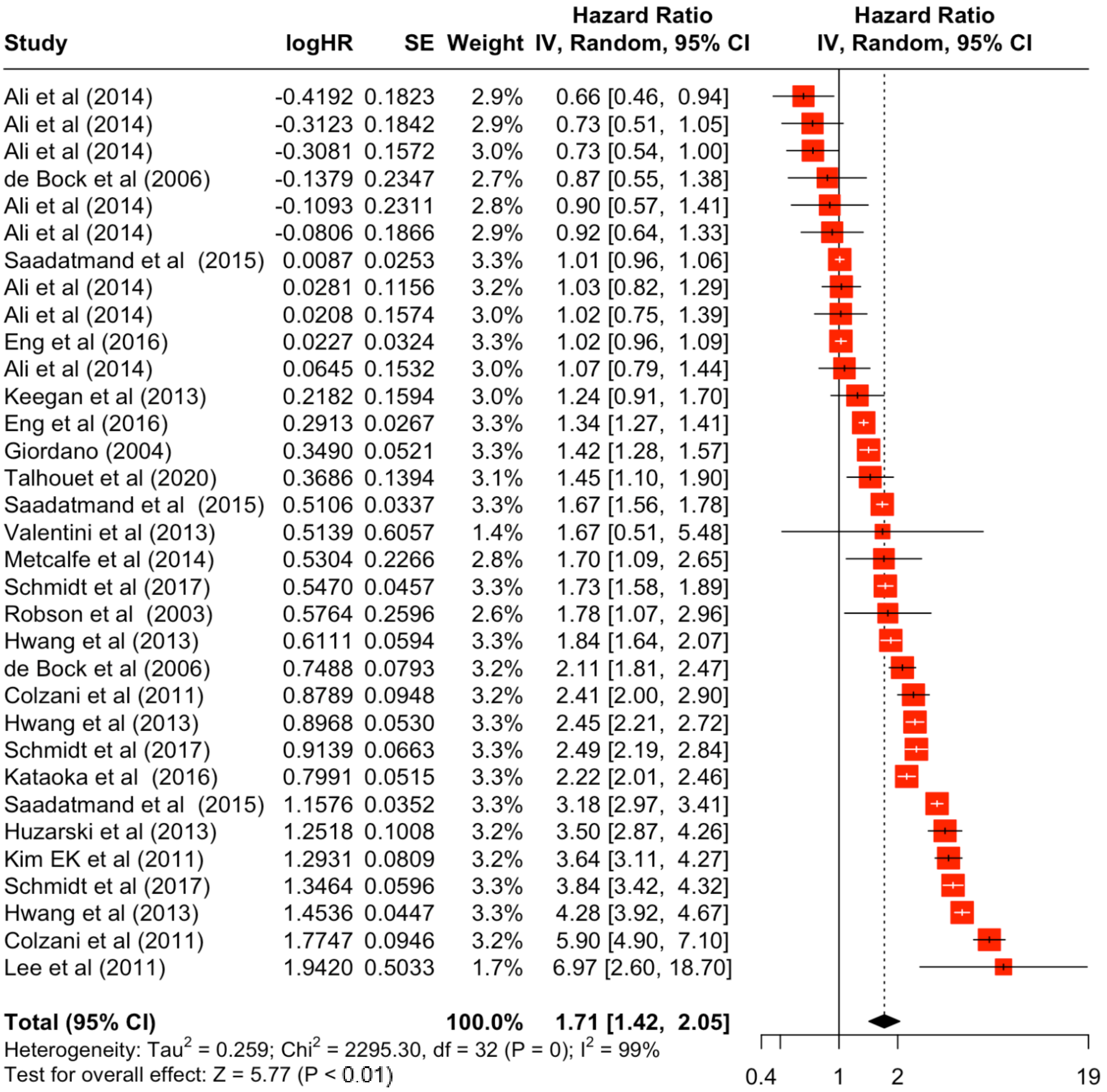

Figure S12: Forest plot for Random-effects Hazard Ratio Model of Tumour size (≥ 2 cm)

Study	logHR	SE	Weight	Hazard Ratio IV, Random, 95% CI
Chagpar et al (2017)	0.0065	0.0003	14.1%	1.01 [1.01, 1.01]
Vinh-Hung et al (2002)	0.0276	0.0010	14.1%	1.03 [1.03, 1.03]
Eng et al (2016)	0.0847	0.0281	8.6%	1.09 [1.03, 1.15]
Giordano (2004)	0.2285	0.0587	3.7%	1.26 [1.12, 1.41]
Lee et al (2011)	0.2159	0.0615	3.5%	1.24 [1.10, 1.40]
Giordano (2004)	0.2980	0.0548	4.1%	1.35 [1.21, 1.50]
Eng et al (2016)	0.3561	0.0286	8.5%	1.43 [1.35, 1.51]
Hwang et al (2013)	0.3676	0.0159	11.7%	1.44 [1.40, 1.49]
Field et al (2005)	0.4064	0.0357	7.0%	1.50 [1.40, 1.61]
de Bock et al (2006)	0.4439	0.0734	2.6%	1.56 [1.35, 1.80]
Metcalfe et al (2014)	0.5041	0.2323	0.3%	1.66 [1.05, 2.61]
Schmidt et al (2017)	0.5267	0.0451	5.3%	1.69 [1.55, 1.85]
Olafsdottir et al (2020)	0.5516	0.1758	0.5%	1.74 [1.23, 2.45]
Valentini et al (2013)	0.5860	0.6425	0.0%	1.80 [0.51, 6.33]
Huzarski et al (2013)	0.6122	0.1407	0.8%	1.84 [1.40, 2.43]
Colzani et al (2011)	0.6595	0.0658	3.1%	1.93 [1.70, 2.20]
Kim EK et al (2011)	0.6845	0.0845	2.1%	1.98 [1.68, 2.34]
Powe et al (2010)	0.6859	0.2369	0.3%	1.99 [1.25, 3.16]
Robson et al (2003)	0.7813	0.2648	0.2%	2.18 [1.30, 3.67]
Field et al (2005)	0.9122	0.0562	4.0%	2.49 [2.23, 2.78]
Schmidt et al (2017)	0.9786	0.0612	3.5%	2.66 [2.36, 3.00]
Olafsdottir et al (2020)	1.0228	0.3184	0.2%	2.78 [1.49, 5.19]
Huzarski et al (2013)	1.5166	0.1851	0.5%	4.56 [3.17, 6.55]
Schmidt et al (2017)	1.7368	0.1337	0.9%	5.68 [4.37, 7.38]
Valentini et al (2013)	1.0789	0.5354	0.1%	2.94 [1.03, 8.40]
**Total (95% CI)**			**100.0%**	**1.39 [1.35, 1.42]**

Heterogeneity: $Tau^2 = 0.001$; $Chi^2 = 2409.95$, df = 24 (P = 0); $I^2 = 99\%$
Test for overall effect: Z = 24.75 (P < 0.01)

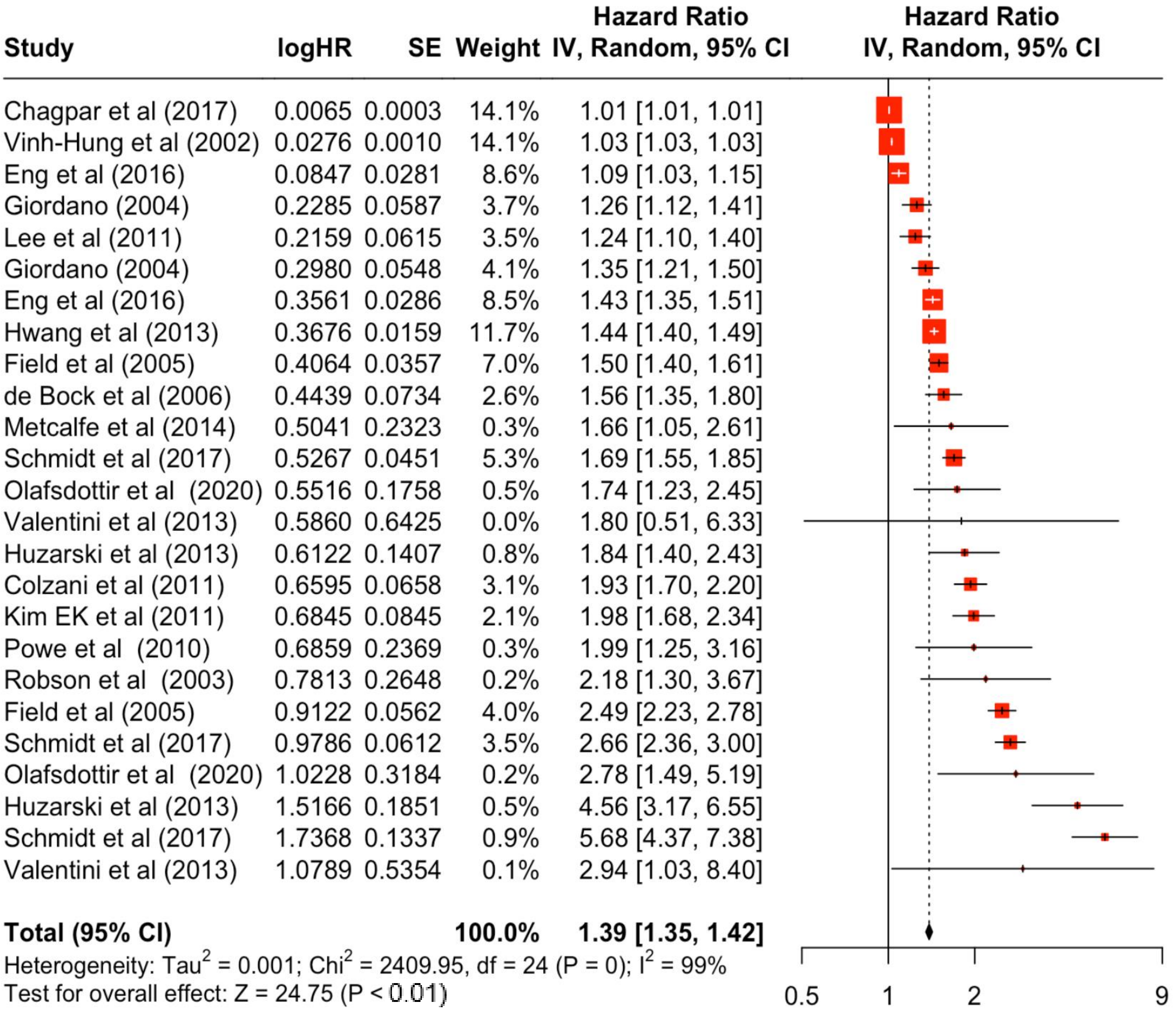

Figure S13: Forest plot for Random-effects Hazard Ratio Model of Hormone Therapy (Yes)

Study	logHR	SE	Weight	Hazard Ratio IV, Random, 95% CI
Lan et al (2013)	-1.4306	0.3110	7.2%	0.24 [0.13, 0.44]
Wray et al (2013)	-0.8052	0.0964	10.5%	0.45 [0.37, 0.54]
Kaplan et al (2015)	-0.7458	0.2338	8.5%	0.47 [0.30, 0.75]
Ali et al (2014)	-0.0662	0.1268	10.2%	0.94 [0.73, 1.20]
Ali et al (2014)	0.1041	0.0847	10.7%	1.11 [0.94, 1.31]
Saadatmand et al (2015)	0.1941	0.0189	11.1%	1.21 [1.17, 1.26]
Bradshaw et al (2016)	0.2576	0.1685	9.6%	1.29 [0.93, 1.80]
Goodwin et al (2011)	0.3980	0.4228	5.5%	1.49 [0.65, 3.41]
Abadi et al (2014)	0.4072	0.1780	9.4%	1.50 [1.06, 2.13]
Goodwin et al (2011)	0.7156	0.3306	6.9%	2.05 [1.07, 3.91]
Abadi et al (2014)	0.6461	0.0931	10.6%	1.91 [1.59, 2.29]
**Total (95% CI)**			**100.0%**	**0.98 [0.74, 1.29]**

Heterogeneity: $Tau^2 = 0.176$; $Chi^2 = 180.85$, df = 10 ($P < 0.01$); $I^2 = 94\%$
Test for overall effect: Z = -0.16 (P = 0.87)

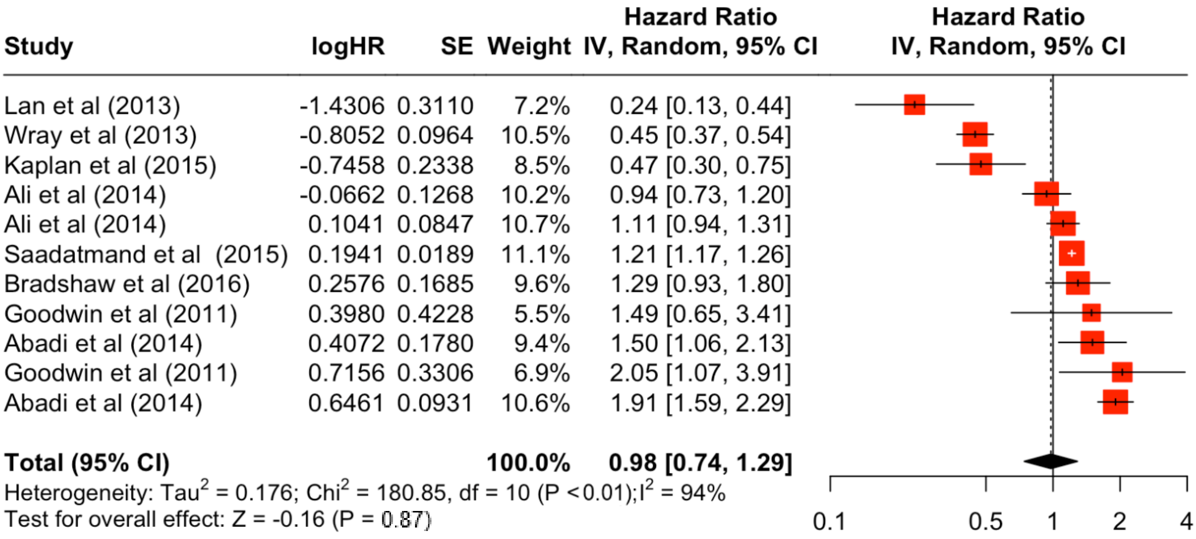

Figure S14: Forest plot for Random-effects Hazard Ratio Model of Chemotherapy (Yes)

Study	logHR	SE	Weight	Hazard Ratio IV, Random, 95% CI
Abadi et al (2014)	-1.7434	0.5459	2.2%	0.17 [0.06, 0.51]
Metcalfe et al (2015)	-0.9587	0.3071	3.2%	0.38 [0.21, 0.70]
Saadatmand et al (2015)	-0.6547	0.0196	4.1%	0.52 [0.50, 0.54]
Valentini et al (2013)	-0.6297	0.4512	2.6%	0.53 [0.22, 1.29]
Ali et al (2014)	-0.4296	0.0858	4.1%	0.65 [0.55, 0.77]
Goodwin et al (2011)	-0.4197	0.3672	2.9%	0.66 [0.32, 1.35]
Robson et al (2017)	-0.4247	0.1686	3.8%	0.65 [0.47, 0.91]
Olafsdottir et al (2020)	-0.2837	0.1696	3.8%	0.75 [0.54, 1.05]
Crew et al (2007)	-0.2318	0.4321	2.6%	0.79 [0.34, 1.85]
Abadi et al (2014)	-0.1282	0.1094	4.0%	0.88 [0.71, 1.09]
de Bock et al (2006)	-0.0607	0.0703	4.1%	0.94 [0.82, 1.08]
Goodwin et al (2011)	-0.0577	0.2849	3.3%	0.94 [0.54, 1.65]
Wray et al (2013)	-0.0381	0.0818	4.1%	0.96 [0.82, 1.13]
Goodwin et al (2011)	-0.0373	0.3245	3.1%	0.96 [0.51, 1.82]
Goodwin et al (2011)	-0.0461	0.2633	3.4%	0.95 [0.57, 1.60]
Abadi et al (2014)	-0.0110	0.1987	3.7%	0.99 [0.67, 1.46]
Ali et al (2014)	0.0173	0.0799	4.1%	1.02 [0.87, 1.19]
Abadi et al (2014)	0.0165	0.1485	3.9%	1.02 [0.76, 1.36]
Capson et al (2018)	0.0240	0.0892	4.1%	1.02 [0.86, 1.22]
Metcalfe et al (2014)	0.1226	0.2373	3.5%	1.13 [0.71, 1.80]
Conroy et al (2011)	0.1577	0.1230	4.0%	1.17 [0.92, 1.49]
Kaplan et al (2015)	0.3329	0.3235	3.1%	1.40 [0.74, 2.63]
Schmidt et al (2017)	0.4506	0.0438	4.1%	1.57 [1.44, 1.71]
Bradshaw et al (2016)	0.5254	0.2194	3.6%	1.69 [1.10, 2.60]
Abadi et al (2014)	0.7242	0.1558	3.9%	2.06 [1.52, 2.80]
Kriege et al (2014)	0.8461	0.3017	3.2%	2.33 [1.29, 4.21]
Abadi et al (2014)	1.0986	0.1378	3.9%	3.00 [2.29, 3.93]
Huzarski et al (2013)	1.3923	0.2470	3.5%	4.02 [2.48, 6.53]
**Total (95% CI)**			**100.0%**	**1.02 [0.81, 1.28]**

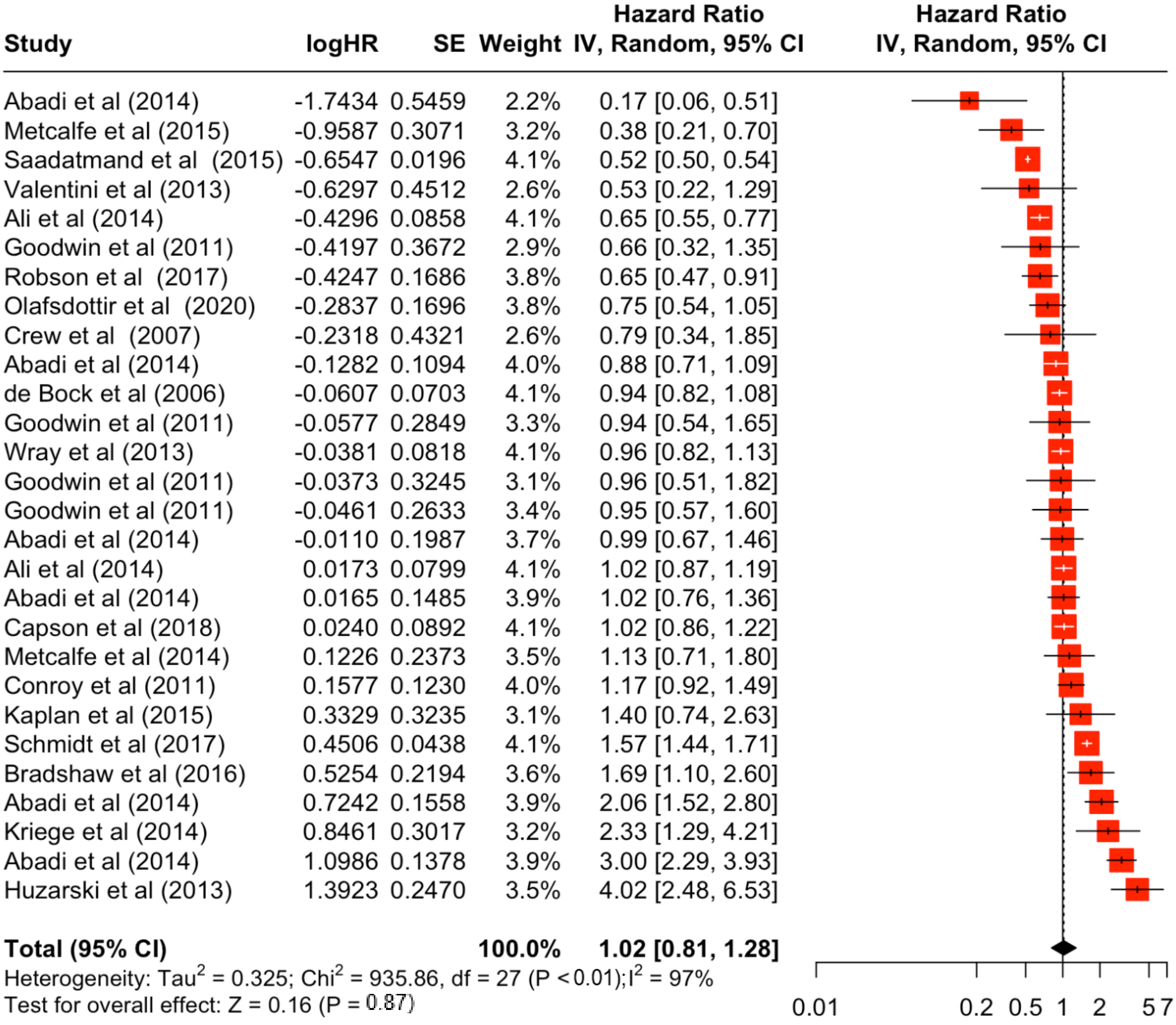

Heterogeneity: $Tau^2 = 0.325$; $Chi^2 = 935.86$, df = 27 ($P < 0.01$); $I^2 = 97\%$
Test for overall effect: Z = 0.16 (P = 0.87)

Figure S15: Forest plot for Random-effects Hazard Ratio Model of Radiotherapy (Yes)

Study	logHR	SE	Weight	Hazard Ratio IV, Random, 95% CI
Saadatmand et al (2015)	-1.0406	0.0195	6.4%	0.35 [0.34, 0.37]
Wray et al (2013)	-0.7327	0.0688	6.4%	0.48 [0.42, 0.55]
Chagpar et al (2017)	-0.3944	0.0265	6.4%	0.67 [0.64, 0.71]
Conroy et al (2011)	-0.2471	0.1261	6.2%	0.78 [0.61, 1.00]
Kaplan et al (2015)	-0.1979	0.2426	5.6%	0.82 [0.51, 1.32]
Crew et al (2007)	-0.1242	0.4041	4.6%	0.88 [0.40, 1.95]
de Bock et al (2006)	0.0015	0.0837	6.3%	1.00 [0.85, 1.18]
Olafsdottir et al (2020)	0.0215	0.1786	6.0%	1.02 [0.72, 1.45]
Bradshaw et al (2016)	0.1661	0.1860	5.9%	1.18 [0.82, 1.70]
Abadi et al (2014)	0.2041	0.1636	6.0%	1.23 [0.89, 1.69]
Abadi et al (2014)	0.2935	0.1058	6.3%	1.34 [1.09, 1.65]
Le et al (2005)	0.3582	0.1114	6.2%	1.43 [1.15, 1.78]
Metcalfe et al (2014)	0.4820	0.2721	5.5%	1.62 [0.95, 2.76]
Abadi et al (2014)	0.7610	0.4712	4.2%	2.14 [0.85, 5.39]
Abadi et al (2014)	0.7697	0.0788	6.3%	2.16 [1.85, 2.52]
Abadi et al (2014)	1.1046	0.1476	6.1%	3.02 [2.26, 4.03]
Abadi et al (2014)	1.1461	0.2709	5.5%	3.15 [1.85, 5.35]
**Total (95% CI)**			**100.0%**	**1.14 [0.83, 1.57]**

Heterogeneity: $Tau^2 = 0.412$; $Chi^2 = 1329.58$, df = 16 ($P < 0.01$); $I^2 = 99\%$
Test for overall effect: $Z = 0.80$ ($P = 0.42$)

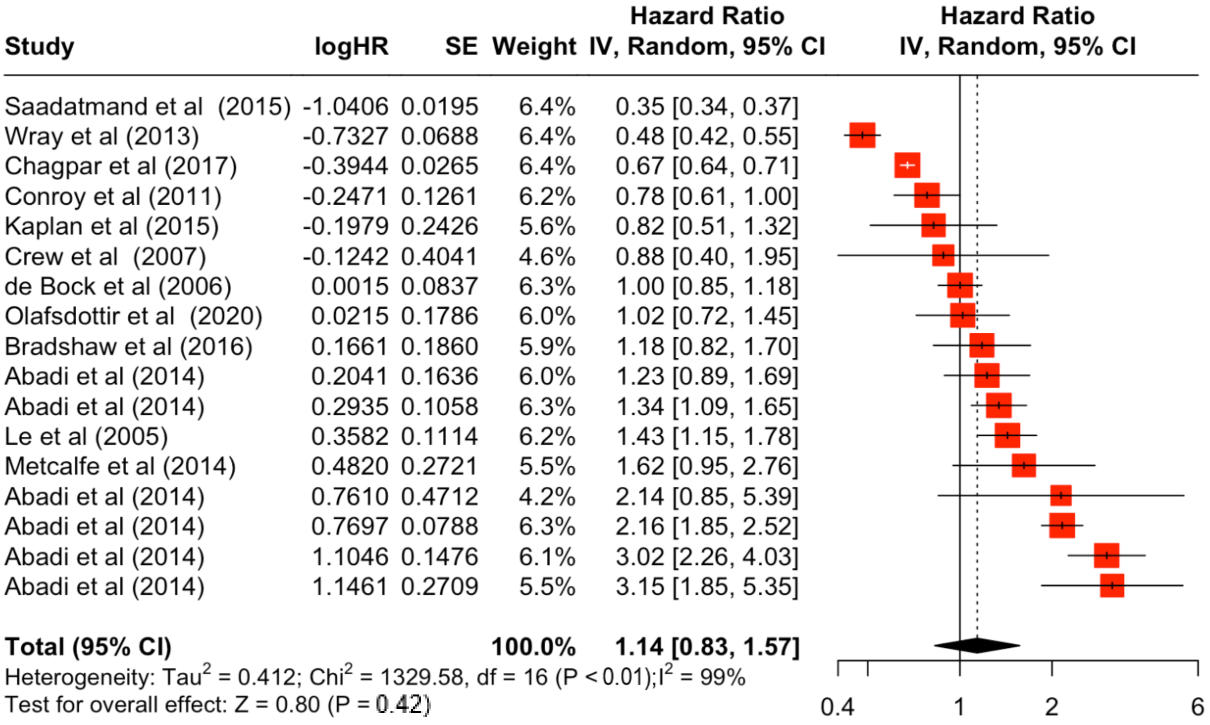

Figure S16: Forest plot for Random-effects Hazard Ratio Model of Tamoxifen

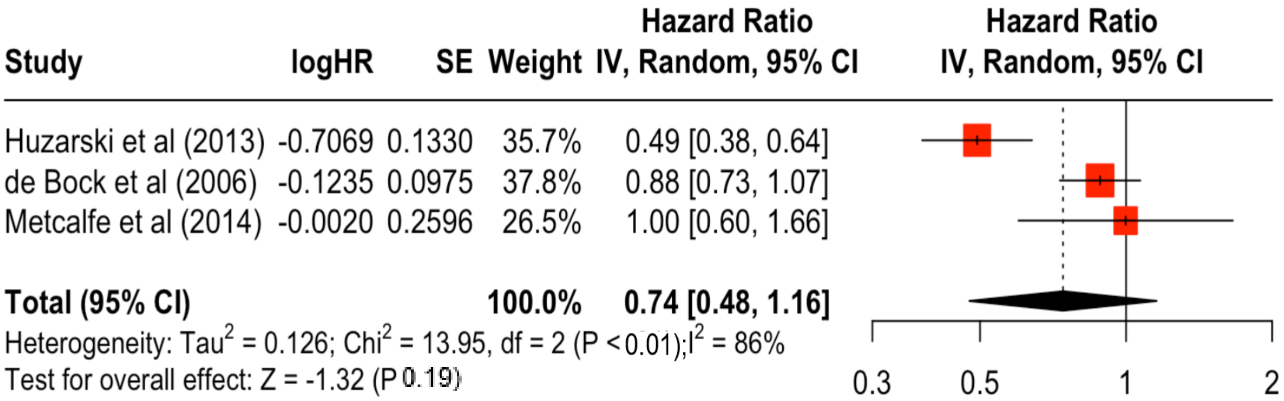

Figure S17: Forest plot for Random-effects Hazard Ratio Model of Histology (Lobular)

Study	logHR	SE	Weight	Hazard Ratio IV, Random, 95% CI
Fallahpour et al (2017)	-0.7873	0.4456	3.9%	0.46 [0.19, 1.09]
Vinh-Hung et al (2002)	-0.3110	0.0403	11.5%	0.73 [0.68, 0.79]
Fallahpour et al (2017)	-0.1879	0.1399	9.8%	0.83 [0.63, 1.09]
Le et al (2005)	0.0831	0.1436	9.7%	1.09 [0.82, 1.44]
Kaffashian et al (2003)	0.1094	0.0820	11.0%	1.12 [0.95, 1.31]
Rapiti et al (2005)	0.1823	0.2069	8.2%	1.20 [0.80, 1.80]
Saadatmand et al (2015)	0.1806	0.0298	11.6%	1.20 [1.13, 1.27]
Ali et al (2014)	0.1870	0.3822	4.7%	1.21 [0.57, 2.55]
Ali et al (2014)	0.1986	0.1724	9.0%	1.22 [0.87, 1.71]
Fallahpour et al (2017)	0.0437	0.5727	2.7%	1.04 [0.34, 3.21]
Hussain et al (2008)	0.5036	0.0569	11.4%	1.65 [1.48, 1.85]
Fallahpour et al (2017)	0.4493	0.2718	6.7%	1.57 [0.92, 2.67]
**Total (95% CI)**			**100.0%**	**1.09 [0.88, 1.34]**

Heterogeneity: $Tau^2 = 0.098$; $Chi^2 = 170.94$, df = 11 (P <0.01); $I^2 = 94\%$
Test for overall effect: Z = 0.81 (P = 0.42)

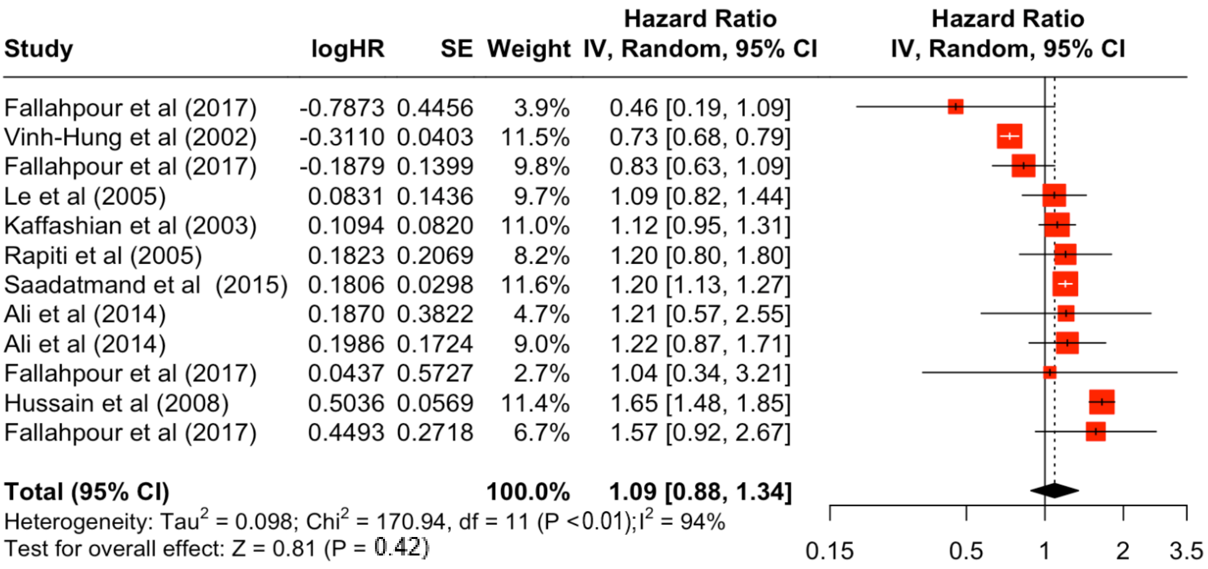

Figure S18: Forest plot for Random-effects Hazard Ratio Model of Histology (Others)

Study	logHR	SE	Weight	Hazard Ratio IV, Random, 95% CI
Fallahpour et al (2017)	-1.5029	0.2011	7.3%	0.22 [0.15, 0.33]
Fallahpour et al (2017)	-0.8500	0.3626	5.1%	0.43 [0.21, 0.87]
Fallahpour et al (2017)	-0.5114	0.3707	5.0%	0.60 [0.29, 1.24]
Fallahpour et al (2017)	-0.3927	0.1848	7.5%	0.68 [0.47, 0.97]
Le et al (2005)	-0.3393	0.1137	8.4%	0.71 [0.57, 0.89]
Ali et al (2014)	-0.2614	0.2306	6.9%	0.77 [0.49, 1.21]
Ali et al (2014)	-0.2604	0.0791	8.7%	0.77 [0.66, 0.90]
Vinh-Hung et al (2002)	-0.0430	0.0295	9.0%	0.96 [0.90, 1.01]
Kaffashian et al (2003)	0.0806	0.0727	8.7%	1.08 [0.94, 1.25]
Ali et al (2014)	0.0779	0.0659	8.8%	1.08 [0.95, 1.23]
Ali et al (2014)	0.0851	0.1702	7.7%	1.09 [0.78, 1.52]
Saadatmand et al (2015)	0.4932	0.0280	9.0%	1.64 [1.55, 1.73]
Rapiti et al (2005)	1.4605	0.1517	7.9%	4.31 [3.20, 5.80]
**Total (95% CI)**			**100.0%**	**0.90 [0.71, 1.15]**

Heterogeneity: $Tau^2 = 0.171$; $Chi^2 = 403.92$, df = 12 (P < 0.01); $I^2 = 97\%$
Test for overall effect: Z = -0.81 (P = 0.42)

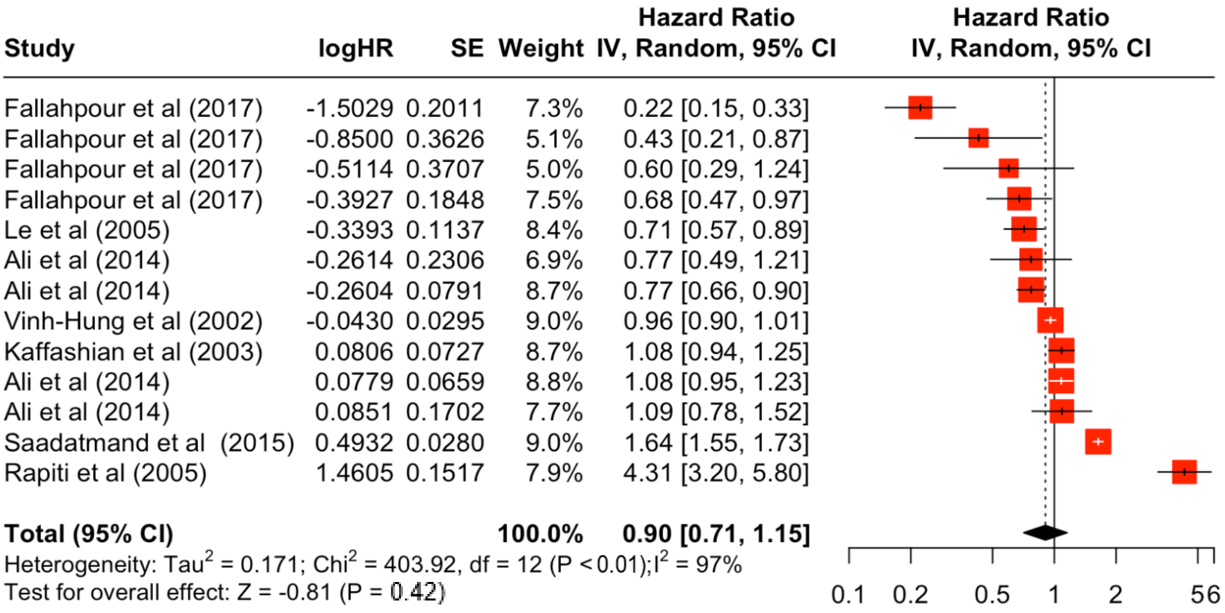

Figure S19: Forest plot for Random-effects Hazard Ratio Model of Progesterone Receptor (Positive)

Study	logHR	SE	Weight	Hazard Ratio IV, Random, 95% CI
Crew et al (2007)	-1.0163	0.6563	2.1%	0.36 [0.10, 1.31]
Wray et al (2013)	-0.8437	0.0768	9.2%	0.43 [0.37, 0.50]
Huzarski et al (2013)	-0.8475	0.0889	9.1%	0.43 [0.36, 0.51]
Saadatmand et al (2015)	-0.5453	0.0176	9.6%	0.58 [0.56, 0.60]
Eng et al (2016)	-0.5034	0.0211	9.6%	0.60 [0.58, 0.63]
Vostakolaei et al (2012)	-0.1755	0.2155	7.0%	0.84 [0.55, 1.28]
Robson et al (2017)	-0.1834	0.2115	7.0%	0.83 [0.55, 1.26]
Giordano (2004)	-0.1262	0.0893	9.1%	0.88 [0.74, 1.05]
Ali et al (2014)	0.0930	0.2080	7.1%	1.10 [0.73, 1.65]
Ali et al (2014)	0.1854	0.0697	9.3%	1.20 [1.05, 1.38]
Maso et al (2008)	0.2703	0.2713	6.0%	1.31 [0.77, 2.23]
Lambertini et al (2020)	0.3194	0.2642	6.1%	1.38 [0.82, 2.31]
Kim EK et al (2011)	1.2896	0.1060	8.8%	3.63 [2.95, 4.47]
**Total (95% CI)**			**100.0%**	**0.87 [0.70, 1.07]**

Heterogeneity: $Tau^2 = 0.120$; $Chi^2 = 468.94$, df = 12 (P <0.01); $I^2 = 97\%$
Test for overall effect: Z = -1.30 (P = 0.19)

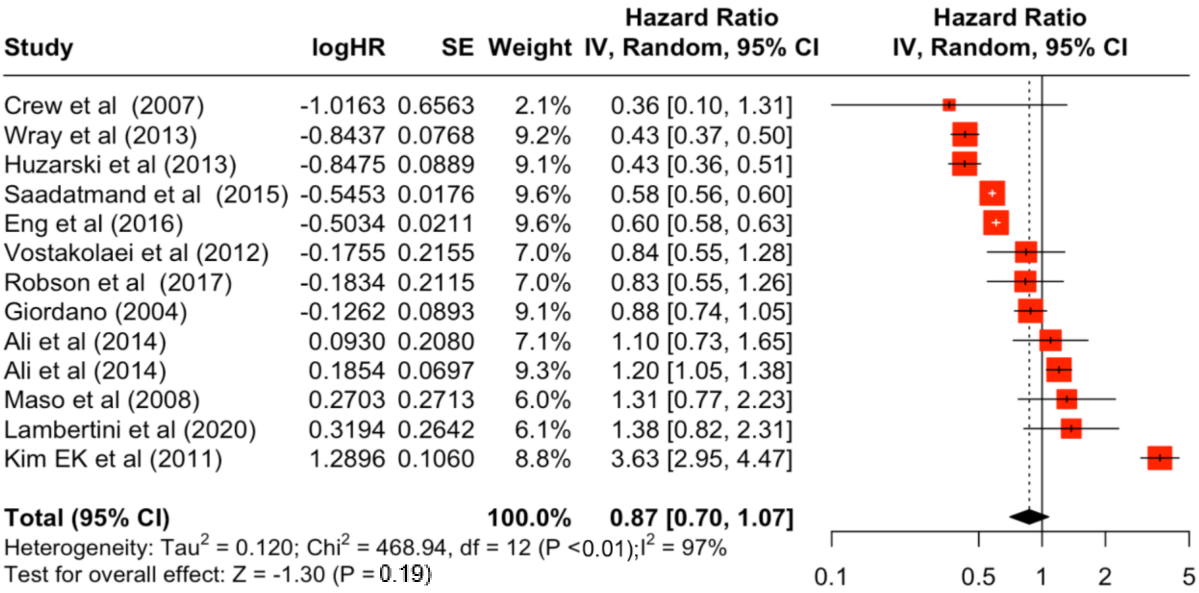

Figure S20: Forest plot for Random-effects Hazard Ratio Model of Progesterone Receptor (Negative)

Study	logHR	SE	Weight	Hazard Ratio IV, Random, 95% CI
Ali et al (2014)	-0.3464	0.0755	14.6%	0.71 [0.61, 0.82]
Lambertini et al (2020)	-0.2139	0.2148	13.0%	0.81 [0.53, 1.23]
Ali et al (2014)	-0.0373	0.1078	14.3%	0.96 [0.78, 1.19]
Field et al (2005)	0.0934	0.0580	14.7%	1.10 [0.98, 1.23]
Vinh-Hung et al (2002)	0.1938	0.0334	14.8%	1.21 [1.14, 1.30]
Maso et al (2008)	0.5049	0.1561	13.8%	1.66 [1.22, 2.25]
Hwang et al (2013)	1.1075	0.0379	14.8%	3.03 [2.81, 3.26]
**Total (95% CI)**			**100.0%**	**1.21 [0.78, 1.88]**

Heterogeneity: $Tau^2 = 0.337$; $Chi^2 = 536.84$, df = 6 (P <0.01); $I^2 = 99\%$

Test for overall effect: Z = 0.86 (P = 0.39)

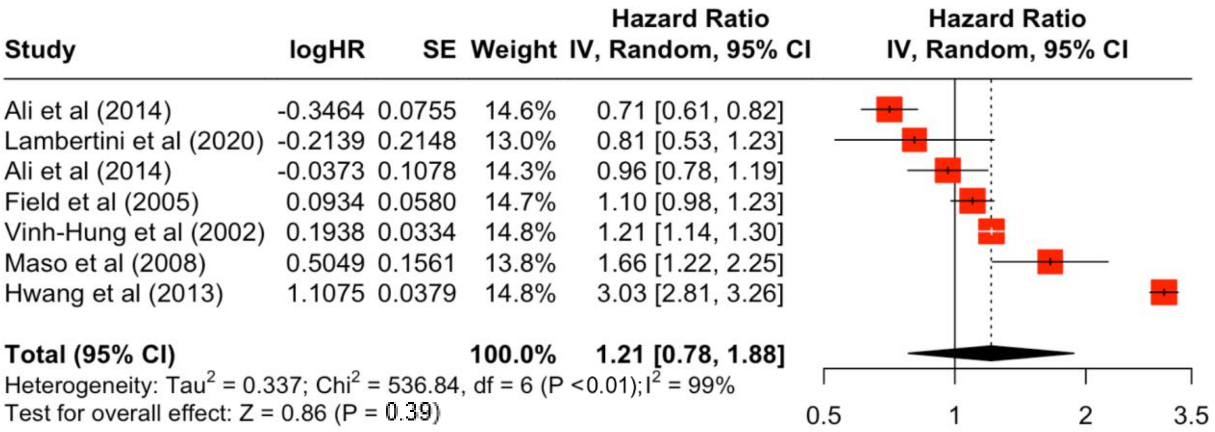

Figure S21: Forest plot for Random-effects Hazard Ratio Model of Estrogen receptor (Negative)

Study	logHR	SE	Weight	Hazard Ratio IV, Random, 95% CI
Metcalfe et al (2015)	-2.7484	0.9474	2.5%	0.06 [0.01, 0.41]
Lambertini et al (2020)	-0.2139	0.2148	13.4%	0.81 [0.53, 1.23]
Field et al (2005)	0.3913	0.0465	17.3%	1.48 [1.35, 1.62]
Vinh-Hung et al (2002)	0.4349	0.0355	17.4%	1.54 [1.44, 1.66]
Maso et al (2008)	0.5049	0.1561	15.1%	1.66 [1.22, 2.25]
Colzani et al (2011)	0.7316	0.0734	16.9%	2.08 [1.80, 2.40]
Hwang et al (2013)	1.1075	0.0379	17.4%	3.03 [2.81, 3.26]
**Total (95% CI)**			**100.0%**	**1.55 [1.13, 2.13]**

Heterogeneity: $Tau^2 = 0.151$; $Chi^2 = 244.50$, df = 6 (P < 0.01); $I^2 = 98\%$

Test for overall effect: Z = 2.69 (P < 0.01)

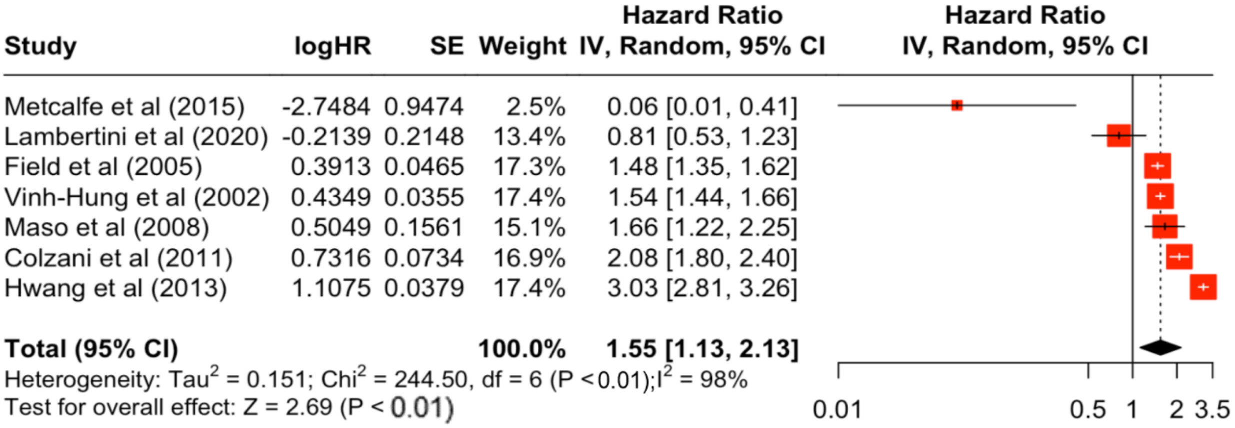

Figure S22: Forest plot for Random-effects Hazard Ratio Model of HER2 receptor (positive)

Study	logHR	SE	Weight	Hazard Ratio IV, Random, 95% CI
Wray et al (2013)	-0.8451	0.5368	4.9%	0.43 [0.15, 1.23]
Ali et al (2014)	-0.3720	0.1246	9.5%	0.69 [0.54, 0.88]
Ali et al (2014)	-0.2724	0.1390	9.4%	0.76 [0.58, 1.00]
Keegan et al (2013)	-0.2211	0.2016	8.7%	0.80 [0.54, 1.19]
Saadatmand et al (2015)	0.0567	0.0289	10.0%	1.06 [1.00, 1.12]
Capson et al (2018)	0.0707	0.0899	9.7%	1.07 [0.90, 1.28]
Kataoka et al (2016)	1.0347	0.0444	9.9%	2.81 [2.58, 3.07]
Keegan et al (2013)	0.4860	0.1901	8.9%	1.63 [1.12, 2.36]
Kataoka et al (2016)	0.6237	0.0627	9.9%	1.87 [1.65, 2.11]
Huzarski et al (2013)	0.7856	0.1153	9.6%	2.19 [1.75, 2.75]
Kim EK et al (2011)	0.8548	0.1141	9.6%	2.35 [1.88, 2.94]
**Total (95% CI)**			**100.0%**	**1.29 [0.93, 1.79]**

Heterogeneity: $Tau^2 = 0.277$; $Chi^2 = 470.86$, df = 10 (P <0.01); $I^2 = 98\%$
Test for overall effect: Z = 1.53 (P = 0.13)

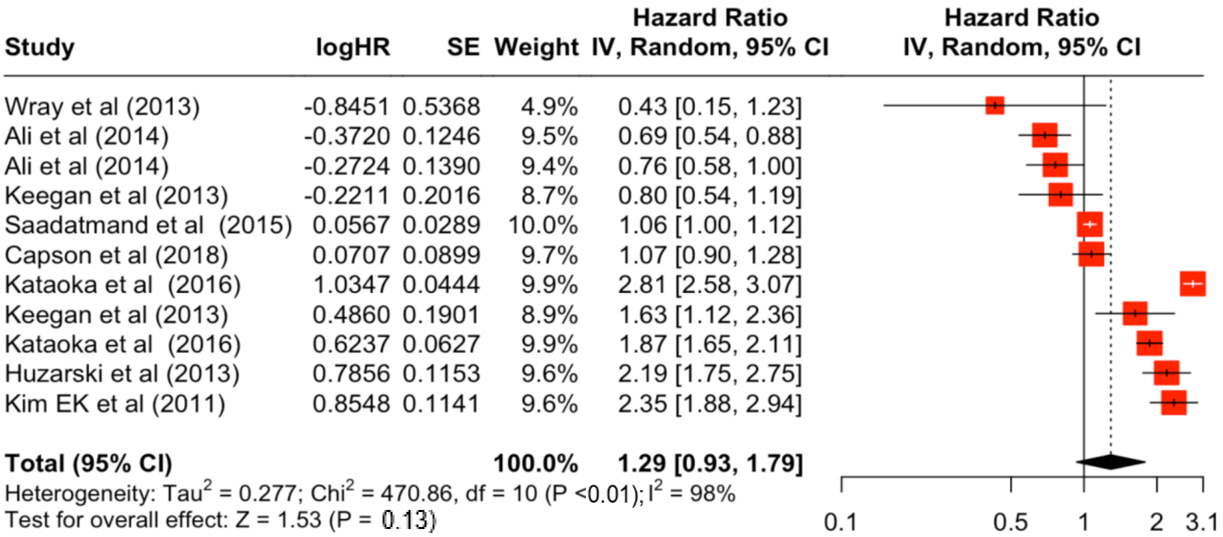

Figure S23: Forest plot for Random-effects Hazard Ratio Model of HER2 receptor (negative)

Study	logHR	SE	Weight	Hazard Ratio IV, Random, 95% CI
Vostakolaei et al (2012)	0.6352	0.1902	27.9%	1.89 [1.30, 2.74]
Ali et al (2014)	-0.2365	0.0837	35.6%	0.79 [0.67, 0.93]
Ali et al (2014)	0.1487	0.0658	36.5%	1.16 [1.02, 1.32]
**Total (95% CI)**			**100.0%**	**1.16 [0.79, 1.70]**

Heterogeneity: $Tau^2 = 0.099$; $Chi^2 = 23.44$, df = 2 (P <0.01); $I^2 = 91\%$

Test for overall effect: Z = 0.76 (P = 0.45)

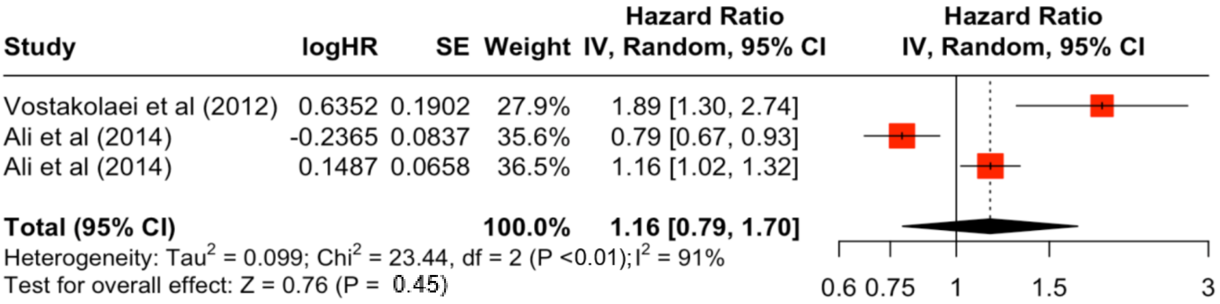

Figure S24: Forest plot for Random-effects Hazard Ratio Model of Physical Activity (light to moderate)

Study	logHR	SE	Weight	Hazard Ratio IV, Random, 95% CI
Rohan et al (1995)	-0.6844	0.3789	7.5%	0.50 [0.24, 1.06]
de Glas et al (2014)	-0.3519	0.7245	2.1%	0.70 [0.17, 2.91]
de Glas et al (2014)	-0.0357	0.5174	4.0%	0.96 [0.35, 2.66]
Maso et al (2008)	0.0396	0.1215	73.4%	1.04 [0.82, 1.32]
Rohan et al (1995)	0.0711	0.2885	13.0%	1.07 [0.61, 1.89]
**Total (95% CI)**			**100.0%**	**0.98 [0.80, 1.20]**

Heterogeneity: $Tau^2 = 0$; $Chi^2 = 3.63$, df = 4 (P =0.46); $I^2 = 0\%$
Test for overall effect: Z = -0.21 (P = 0.83)

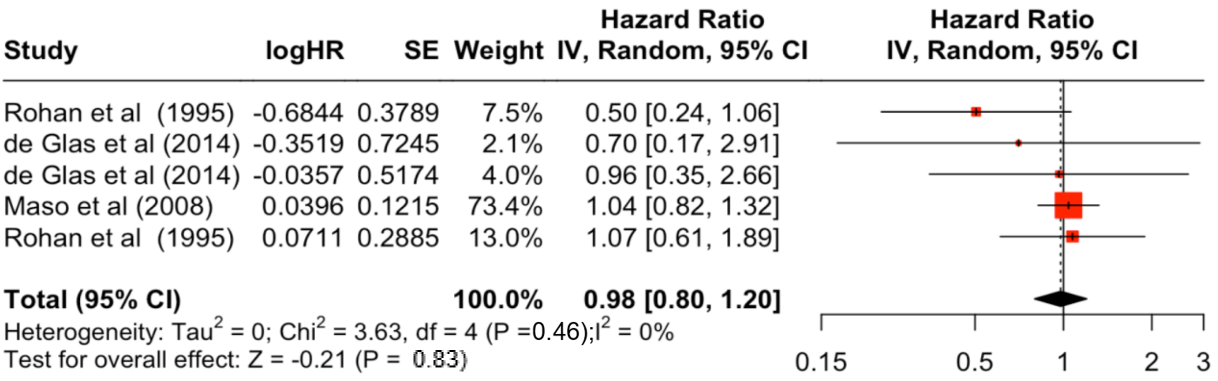

Figure S25: Forest plot for Random-effects Hazard Ratio Model of Physical Activity (high to vigorous)

Study	logHR	SE	Weight	Hazard Ratio IV, Random, 95% CI
de Glas et al (2014)	-1.4017	0.7203	6.6%	0.25 [0.06, 1.01]
de Glas et al (2014)	-0.4788	0.6030	9.1%	0.62 [0.19, 2.02]
de Glas et al (2014)	-0.0922	0.6210	8.6%	0.91 [0.27, 3.08]
de Glas et al (2014)	0.0272	0.5796	9.8%	1.03 [0.33, 3.20]
Maso et al (2008)	0.1040	0.1606	52.4%	1.11 [0.81, 1.52]
Rohan et al (1995)	0.5559	0.4804	13.5%	1.74 [0.68, 4.47]
**Total (95% CI)**			**100.0%**	**0.99 [0.68, 1.44]**

Hazard Ratio IV, Random, 95% CI

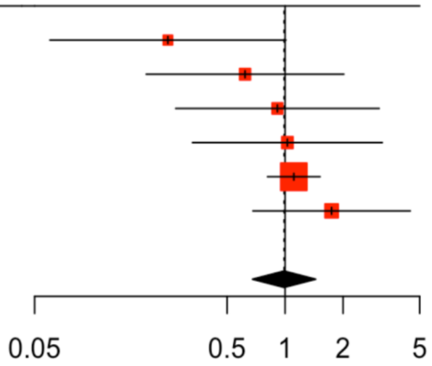

Heterogeneity: $Tau^2 = 0.045$; $Chi^2 = 6.10$, df = 5 (P =0.30); $I^2 = 18\%$
Test for overall effect: Z = -0.06 (P = 0.95)

Figure S26: Forest plot for Random-effects Hazard Ratio Model of Body Mass Index (Overweight to Obese)

Study	logHR	SE	Weight	Hazard Ratio IV, Random, 95% CI
Peel et al (2009)	-0.1356	0.1830	5.2%	0.87 [0.61, 1.25]
Ewertz et al (2011)	0.0082	0.0303	15.8%	1.01 [0.95, 1.07]
Ewertz et al (2011)	0.0707	0.0569	13.8%	1.07 [0.96, 1.20]
Ewertz et al (2011)	0.0828	0.0422	15.0%	1.09 [1.00, 1.18]
Conroy et al (2011)	0.1533	0.1552	6.4%	1.17 [0.86, 1.58]
Ewertz et al (2011)	0.2879	0.0800	11.7%	1.33 [1.14, 1.56]
Nichols et al (2009)	0.2965	0.2463	3.3%	1.35 [0.83, 2.18]
Capson et al (2018)	0.2995	0.0996	10.1%	1.35 [1.11, 1.64]
Maso et al (2008)	0.3202	0.1533	6.5%	1.38 [1.02, 1.86]
Conroy et al (2011)	0.3710	0.1644	6.0%	1.45 [1.05, 2.00]
Johnsson et al (2017)	0.4659	0.2803	2.7%	1.59 [0.92, 2.76]
Nichols et al (2009)	0.8248	0.2383	3.5%	2.28 [1.43, 3.64]
**Total (95% CI)**			**100.0%**	**1.20 [1.09, 1.33]**

Heterogeneity: $Tau^2 = 0.015$; $Chi^2 = 36.79$, df = 11 (P <0.01); $I^2 = 70\%$

Test for overall effect: Z = 3.69 (P < 0.01)

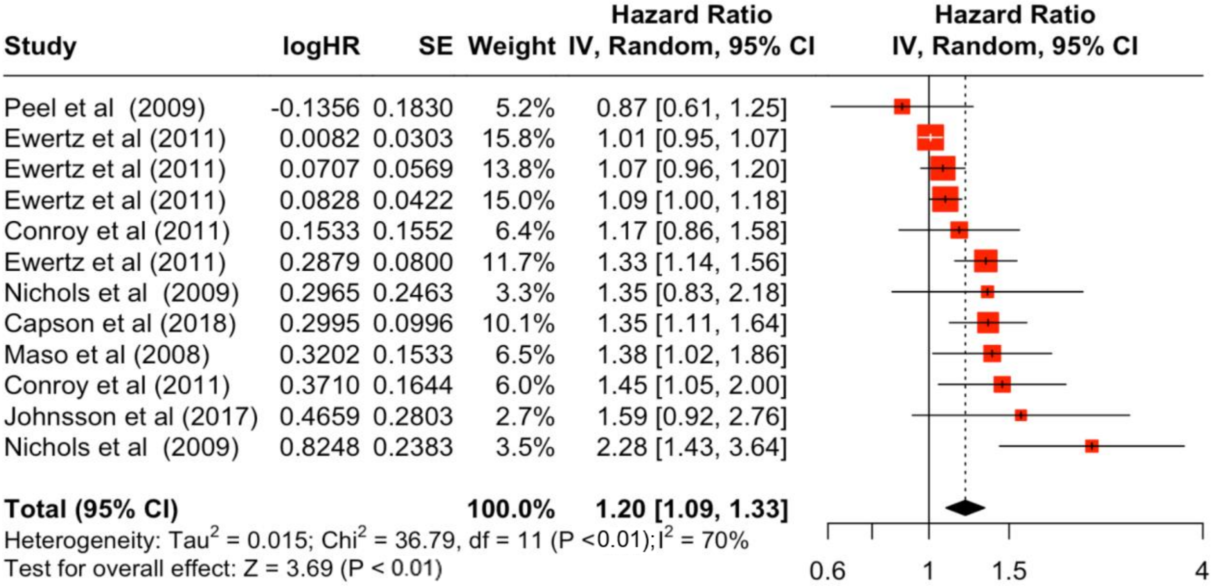

Figure S27: Forest plot for Random-effects Hazard Ratio Model of BRCA 1

Study	logHR	SE	Weight	Hazard Ratio IV, Random, 95% CI
Metcalfe et al (2015)	-1.0232	0.3253	9.5%	0.36 [0.19, 0.68]
Robson et al (2017)	-0.6150	0.1935	12.2%	0.54 [0.37, 0.79]
Lee et al (2011)	-0.5816	0.4106	7.9%	0.56 [0.25, 1.25]
Talhouet et al (2020)	-0.4632	0.1826	12.4%	0.63 [0.44, 0.90]
Lambertini et al (2020)	-0.3544	0.2154	11.8%	0.70 [0.46, 1.07]
Rennert et al (2007)	0.0800	0.2084	11.9%	1.08 [0.72, 1.63]
Schmidt et al (2017)	0.2499	0.1026	13.8%	1.28 [1.05, 1.57]
Goodwin et al (2011)	0.3538	0.2287	11.5%	1.42 [0.91, 2.23]
Robson et al (2003)	0.8702	0.3510	9.0%	2.39 [1.20, 4.75]
**Total (95% CI)**			**100.0%**	**0.86 [0.61, 1.19]**

Heterogeneity: $Tau^2 = 0.198$; $Chi^2 = 45.20$. df = 8 (P <0.01); $I^2 = 82\%$
Test for overall effect: Z = -0.91 (P = 0.36)

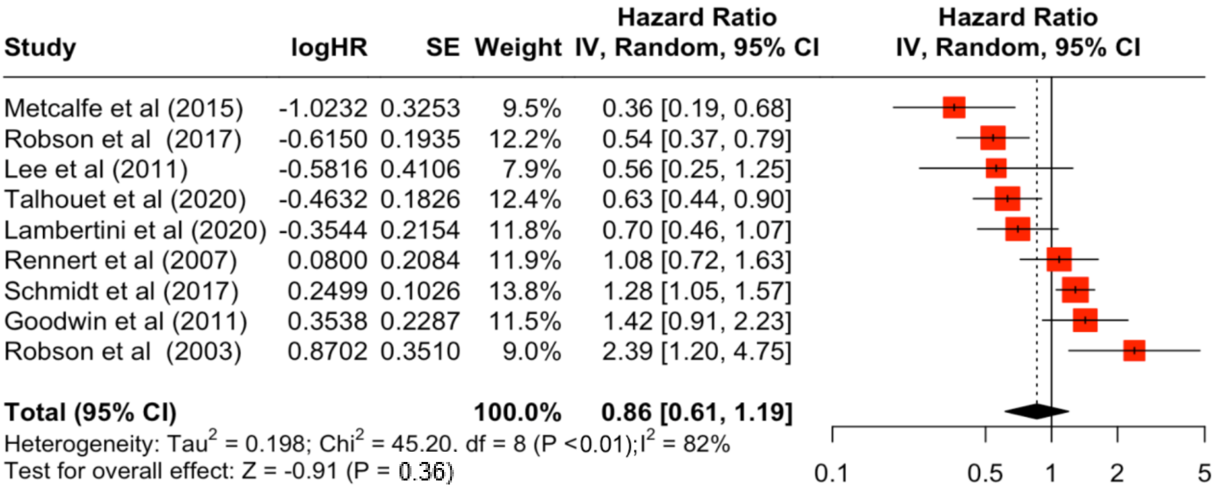

Figure S28: Forest plot for Random-effects Hazard Ratio Model of BRCA 2

Study	logHR	SE	Weight	Hazard Ratio IV, Random, 95% CI
Metcalfe et al (2014)	-0.4385	0.2438	10.4%	0.64 [0.40, 1.04]
Robson et al (2017)	-0.3654	0.2210	11.1%	0.69 [0.45, 1.07]
Metcalfe et al (2015)	-0.3604	0.3975	6.7%	0.70 [0.32, 1.52]
Talhouet et al (2020)	-0.3299	0.2169	11.2%	0.72 [0.47, 1.10]
Metcalfe et al (2004)	-0.3076	0.2283	10.9%	0.74 [0.47, 1.15]
Oden et al (2016)	0.1043	0.4721	5.4%	1.11 [0.44, 2.80]
Schmidt et al (2017)	0.2269	0.1639	12.8%	1.25 [0.91, 1.73]
Rennert et al (2007)	0.3503	0.2212	11.1%	1.42 [0.92, 2.19]
Goodwin et al (2011)	0.5953	0.2324	10.8%	1.81 [1.15, 2.86]
Lambertini et al (2020)	0.6308	0.2732	9.6%	1.88 [1.10, 3.21]
**Total (95% CI)**			**100.0%**	**1.02 [0.78, 1.33]**

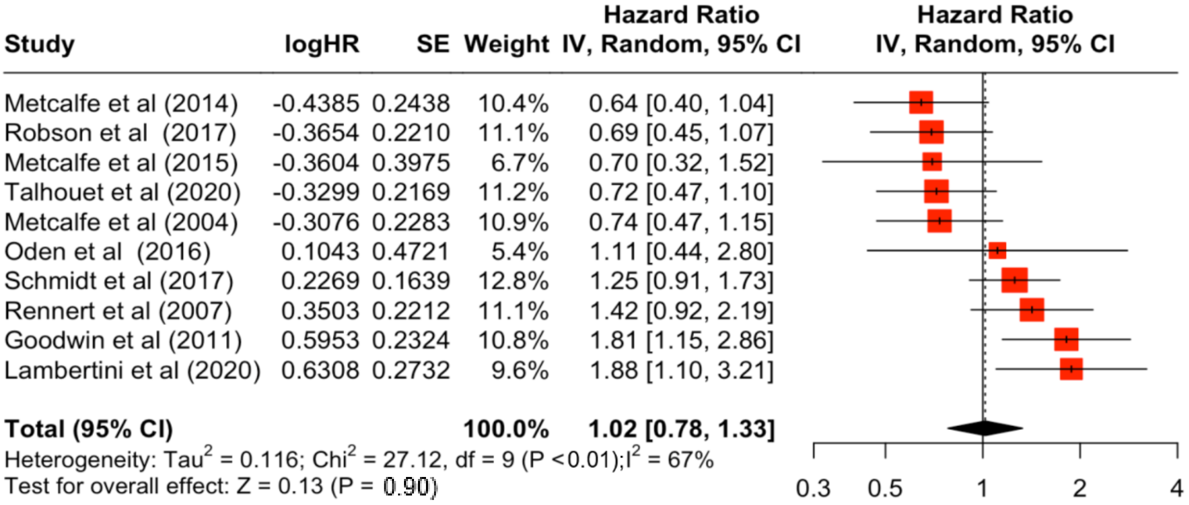

Heterogeneity: $Tau^2 = 0.116$; $Chi^2 = 27.12$, df = 9 ($P < 0.01$); $I^2 = 67\%$
Test for overall effect: Z = 0.13 (P = 0.90)

Figure S29: Forest plot for Random-effects Hazard Ratio Model of Oral Contraceptive Use

Study	logHR	SE	Weight	Hazard Ratio IV, Random, 95% CI
Peel et al (2009)	-0.1560	0.1810	71.6%	0.86 [0.60, 1.22]
Oden et al (2016)	0.1333	0.6036	6.4%	1.14 [0.35, 3.73]
Johnsson et al (2017)	0.3386	0.3264	22.0%	1.40 [0.74, 2.66]
**Total (95% CI)**			**100.0%**	**0.97 [0.72, 1.31]**

Heterogeneity: $Tau^2 = 0$; $Chi^2 = 1.83$, df = 2 (P = 0.40); $I^2 = 0\%$

Test for overall effect: Z = -0.19 (P = 0.85)

Hazard Ratio
IV, Random, 95% CI

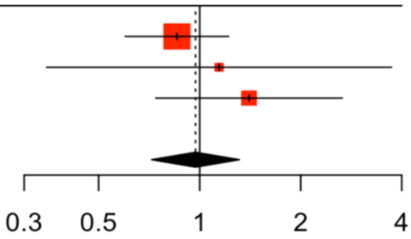

Figure S30: Forest plot for Random-effects Hazard Ratio Model of Comorbidity Index (1 to 2)

Study	logHR	SE	Weight	Hazard Ratio IV, Random, 95% CI
Fallahpour et al (2017)	0.2202	0.4763	10.8%	1.25 [0.49, 3.17]
Crew et al (2007)	0.4276	0.4002	14.5%	1.53 [0.70, 3.36]
Fallahpour et al (2017)	0.4560	0.2430	29.8%	1.58 [0.98, 2.54]
Fallahpour et al (2017)	0.3888	0.4423	12.3%	1.48 [0.62, 3.51]
Fallahpour et al (2017)	0.8842	0.2943	23.2%	2.42 [1.36, 4.31]
Crew et al (2007)	1.6403	0.5175	9.4%	5.16 [1.87, 14.22]
**Total (95% CI)**			**100.0%**	**1.87 [1.35, 2.61]**

Heterogeneity: $Tau^2 = 0.037$; $Chi^2 = 6.36$, df = 5 (P =0.27); $I^2 = 21\%$

Test for overall effect: Z = 3.72 (P < 0.01)

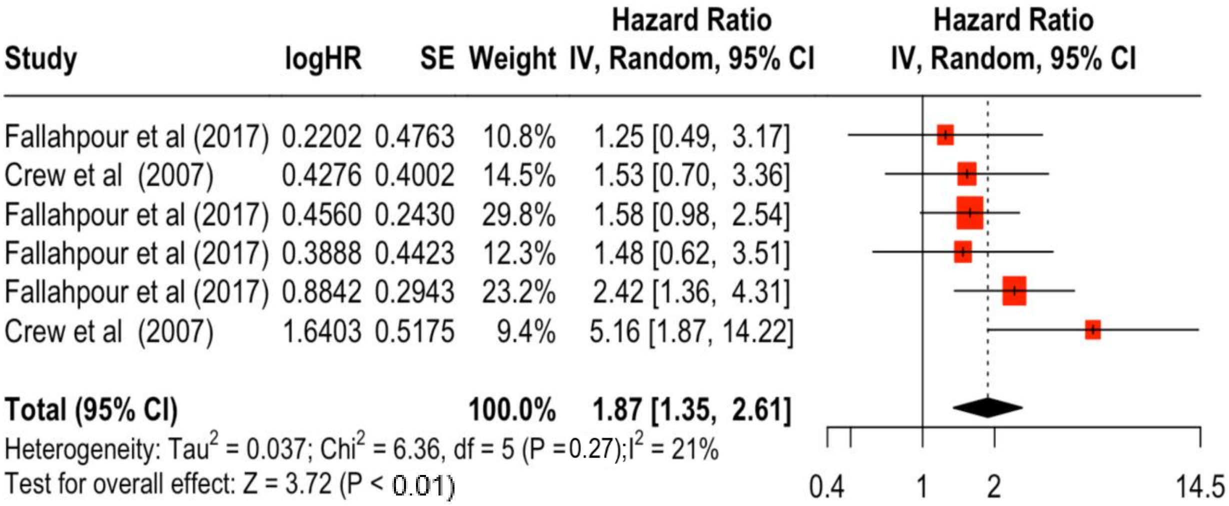

Supplement: Supplementary file 1 [file diseases-12-00111-s001.zip › diseases-2980345-Supplementary File S1.pdf]
